# Research landscape of experiments on global change effects on mycorrhizas

**DOI:** 10.1111/nph.70452

**Published:** 2025-08-08

**Authors:** Anika Lehmann, Bo Tang, Rebecca Rongstock, Alexa Sommerburg, Natalja Chramova, Kevser Ergül, Katharina Heydebreck, Nasrin Quiram, Stefanie Maaß, Eva F. Leifheit, Matthias C. Rillig

**Affiliations:** ^1^ Institute of Biology Freie Universität Berlin Berlin 14195 Germany; ^2^ Berlin‐Brandenburg Institute of Advanced Biodiversity Research (BBIB) Berlin 14195 Germany

**Keywords:** arbuscular mycorrhiza, climate change, global change, multiple factors, mycorrhiza, systematic mapping

## Disclaimer

The New Phytologist Foundation remains neutral with regard to jurisdictional claims in maps and in any institutional affiliations.

## Introduction

Understanding how global environmental change, the entirety of human influences on this planet, affects terrestrial ecosystems is a central research challenge in ecology. Studying the effects of such anthropogenically caused factors requires a formidable effort, because global change includes a large number of biological, chemical and physical factors (Pirotta *et al*., 2022; Orr *et al*., 2024). Importantly, these factors also co‐occur, giving rise to the possibility of complex interactions (Piggott *et al*., 2015). High‐dimensional threats to terrestrial ecosystems, meaning effects arising from the joint action of multiple factors, are only now beginning to be studied experimentally (Rillig *et al*., 2019; Zandalinas *et al*., 2021; Speißer *et al*., 2022; Bi *et al*., 2024).

Another key challenge is the complexity of terrestrial ecosystems themselves, and especially of the soil biota; the biological community of soil is high in biodiversity and harbors divergent functional groups (Fitter *et al*., 2005; Bardgett & van der Putten, 2014; Anthony *et al*., 2023). One of the best‐researched groups is mycorrhizal fungi, forming symbiotic associations with roots of the vast majority of plants (Brundrett & Tedersoo, 2018). These plant–fungal associations at the interface of the root and the soil have pervasive effects on ecosystems because they can influence plant performance, plant communities and soils. It is thus impossible to understand the effects of factors of global change on plants and terrestrial ecosystems without a solid grasp of how mycorrhizal associations react to such factors and how they might modify plant or soil responses to them.

Given the frequently fragmented nature of the vast field of global change biology, research on mycorrhizas is similarly spread across a very large range of study parameters, and almost inevitably spread too thin in many cases. This limits our ability to reach clear conclusions about effects for combinations of global change factors, geographical regions, mycorrhizal types and study conditions. Where are our largest gaps, and, conversely, where do we perhaps already know a sufficient amount? Available syntheses tend to focus on certain global change factors (e.g. invasive species, drought or warming) (Kivlin *et al*., 2013; Augé *et al*., 2014; Tang *et al*., 2023), not offering a higher level picture of the entirety of global change. In order to gauge our level of understanding of mycorrhizal responses to the full range of global change factors, it is important to survey several key parameters: how well the different types of mycorrhizal fungi have been covered (Kivlin *et al*., 2013; Zhou *et al*., 2022); the geographic representation of experimental evidence (Mohan *et al*., 2014; Tedersoo & Bahram, 2019); the degree to which the whole range of global change factors has been addressed in experiments, including their interactions (Bueno *et al*., 2022); and the nature of the evidence, that is from field or lab experimentation. Unfortunately, such a summary has not been available, leaving the extent of knowledge about the responses of this important symbiosis to global change quite unclear.

Here, we provide a systematic overview of the literature on experiments covering global change effects on mycorrhizal fungi. In doing so, we identify several important gaps in the research field. To drive progress in global change biology, it is vital that such gaps are addressed in future research efforts, highlighting the need for more intense collaboration between global change biology and mycorrhizal ecologists. Our detailed map of the research on this topic is intended to serve as a guide for such a research agenda. The assessment we offer here is based on a detailed analysis of the number of studies that have addressed different parameters of this research topic, surpassing previous efforts in scope, detail and depth. We restrict our analysis to experiments, since experiments offer the clearest path to causal understanding of effects, and this is especially important in global change biology (including climate change).

## Materials and Methods

### Systematic mapping

Here, we conducted a systematic literature analysis (systematic mapping). Systematic maps are overviews of a defined evidence base and are used to collate, categorize and describe the research evidence pertaining to a specific subject matter. The evidence data are collected as metadata for each included study. The resultant database is used to identify knowledge clusters (e.g. well‐represented evidence suitable for detailed analyses via systematic reviews or meta‐analyses) or to detect knowledge gaps (i.e. underrepresented evidence suitable for targeted primary research). Similar to other systematic synthesis methodologies, there are systematic map guidelines aiming at enhancing objectivity and transparency while mitigating reviewer selection and publication biases. Our systematic mapping complies with the RepOrting standards for Systematic Evidence Synthesis (ROSES) guideline for systematic maps (Haddaway *et al*., 2018).

### Search and database

For this analysis, we generated 22 search strings covering 15 different global change factors (Supporting Information Methods S1; Tables S1–S6). We ran the article search in Web of Science Core Collection (WoS‐CC) including Science Citation and Emerging Sources Citation Index in January 2021 and extracted the document‐type ‘articles’ with no exclusion for language or publication year; due to English language search strings, the resulting article outcomes have at least an English language title and/or abstract. We extracted 6484 articles published in the years 1969 to 2021. The year 2021 was not complete at that time point. Thus, in a second search run in January 2022, we collected all articles with the publication year 2021 to complete the publication year 2021 in our database. For this second run, we extracted 623 new and unique articles. The articles from both extractions were screened and analyzed by the same team following the same procedure and rules.

To build our database, we included the bibliometric data for author, title, journal and publication year for each article. The articles were assigned random numbers, sorted and subsequently assigned to seven subsets of equal article numbers. We evaluate the performance of global change factor‐specific search strings at the end of the screening procedure (Table S7).

### Screening and coding

The screening was carried out by six screeners and two cross‐checkers (cross‐checking rate: 37.6%). The article subsets were randomly assigned to screeners. Cross‐checkers trained the screeners by coscreening for 100 articles. For questions which appeared during coding, cross‐checker support was requested until a consensus was reached.

The extracted articles were evaluated to determine whether they matched our eligibility criteria. Articles had to present results from an experiment with at least one global change factor covered by our search terms (Table S5). The global change factors and a respective control (e.g. ambient condition) had to be applied by the experimenters (excluding e.g. observational studies, space for time substitutions, natural gradients and slopes). Mycorrhizal fungi had to be part of the study as either a treatment (e.g. inoculum added vs not added) or a response variable (e.g. root colonization or community metric measured).

Articles matching the eligibility criteria were further screened as follows. First, the global change factor treatment was evaluated. We noted only the presence, not the treatment levels (e.g. four temperature levels were counted as one warming treatment). For each global change factor, eligibility criteria were specified (Table S5). We noted the number of global change factors tested and whether these factors were applied in combination or investigated in separate experiments. Second, we collected information on the type of mycorrhizal fungi (e.g. arbuscular mycorrhiza (AM) fungi) and whether the mycorrhizal fungi were manipulated as treatment. Third, specifically for AM fungi, we noted genus and species names and measured AM fungal parameters and community metric indices. Fourth, we noted general system parameters: setting (lab or field study), sterility (sterile vs nonsterile growth substrate) and study location (decimal degrees of field study location or for lab studies the first author affiliation location) (Table S6).

Articles that could not be accessed were requested from authors and the library of the Botanical Garden and Botanical Museum of Berlin, Germany.

### Analysis

The final data table underwent a quality assurance by the cross‐checkers to eliminate typos and coding mistakes. During this step, AM fungal names were harmonized (Schüßler, 2024). The analysis and figure production were performed in R (v.4.2.2) with the packages ggplot2 and ggpubr (Wickham, 2016; R Core Team, 2021; Kassambara, 2023), igraph for the network plots (Csardi & Nepusz, 2005) and sf for the world maps (Pebesma, 2018). We applied the global change factor classification scheme by Rillig *et al*. (2021) (Table S5) to meaningfully sort the global change factors in our display items.

To determine the proportion of our retrieved articles on ‘mycorrhizal fungi in the context of global change’ in the general mycorrhiza research, we conducted an additional search in the WoS‐CC with default settings in January 2023. We ran a topic search with the search string TS = (“mycorrhiza*”) to acquire article output per year. We collected data until the year 2021, which is the final year of our database. No further Web of Science categories were excluded. With the extracted data, we were able to estimate the contribution of ‘global change and mycorrhizal fungi’ research in the broader field of ‘mycorrhizal’ research.

To evaluate any shifts in publication patterns in the years since 2021, we repeated the original searches and the one on general mycorrhiza research with the previously described settings. We compared the search outcomes for the time period until 2021 (the original search) and since 2021. For the topics ‘microplastic’ and ‘multiple factors’, we evaluated whether they fulfilled our first two eligibility criteria about the tested global change factor treatment and mycorrhizal fungi. With this additional analysis, we tested for changes in the research landscape in the last 3 years.

## Results

### The mycorrhiza evidence base

**Fig. 1:**
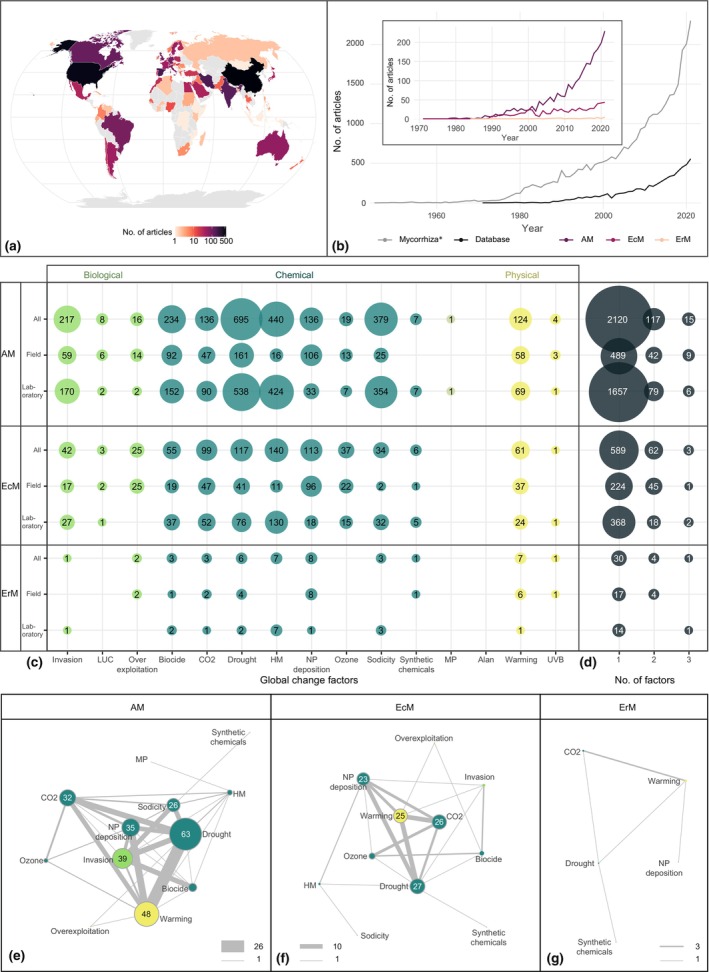
Overview of key data of the ‘Mycorrhiza’ database. (a) Spatial distribution of collected data across the globe. Color gradient represents the number of database entries located in each country, based on study location data for field studies and first author affiliation for lab or unspecified studies. (b) Temporal trends for publication years of general ‘mycorrhiza*’ literature (in gray) compared with mycorrhiza literature in the context of global change (in black) as covered here in our database. In the inset plot, temporal trends for the top three mycorrhiza types (arbuscular mycorrhizal (AM) fungi, ectomycorrhizal (EcM) fungi and ericoid mycorrhizal (ErM) fungi) are shown. (c) Balloon plot showing the number of occurrences of experiments for different mycorrhizal fungi types (AM, EcM and ErM) for all studies combined and separate for lab and field studies for different global change factors tested. The factors were grouped into biological (species invasion, land use change and overexploitation), chemical (biocide, elevated atmospheric carbon dioxide, drought, heavy metals, nitrogen and phosphorus deposition, elevated tropospheric ozone, sodicity and synthetic chemicals), chemical–physical (microplastic) and physical (artificial light at night, warming and ultraviolet B radiation). (d) As an expansion to the number of occurrences of individual factors, the number of cases for factors tested in combination are presented. (e–g) Network plots depicting which factors were tested simultaneously in AM, EcM or ErM fungal experiments. Color coding follows panel (c), node size and edge thickness represent cases of occurrences (see Supporting Information Table S9, for exact values). For all data, only unique cases per article were counted (e.g. an article reporting on lab and field studies provides only one data entry for the category ‘all’).

Of the overall 7107 articles, 2884 matched our eligibility criteria by reporting on experiments with global change factors as treatment and mycorrhizal fungal species as organisms of interest (either tested as treatment themselves or measured as response variable) (Fig. S1). Six different mycorrhizal fungal types were covered by our database: with 76.5% of the database entries providing experiments on AM fungi, 22.1% on ectomycorrhizal (EcM) fungi, 1.2% on ericoid mycorrhizal (ErM) fungi, 0.2% on orchid mycorrhizal fungi, 0.03% on arbutoid mycorrhizal fungi and 0.03% on ectendomycorrhizal fungi (Fig. S2). The reported research was conducted on all five continents (either the location of the field experiment or the affiliation of the first author; Fig. 1a): 3.9% of the database entries came from Africa, 34.8% from Asia, 2.3% from Australia, 31.0% from Europe, 20.9% from North America and 7.1% from South America. The database comprises the publication years 1971 to 2021, while the general research on mycorrhizal fungi ranges back to the earliest publication year available in WoS‐CC. As research on mycorrhizal fungi generally increased exponentially over the last century, articles presenting data on experiments covering mycorrhizal fungi in the contxt of global change represent 24.3% of the general mycorrhizal literature in the year 2021 (Fig. 1b) and 39.6% in the past 3 years (Table S8). The database entries for AM fungal studies surpass those for EcM and ErM fungi (Fig. 1b inlet).

The 15 global change factors varied in their coverage in our database (Figs 1c, S2). For AM fungi, the research focus is on drought (28.8% of the respective database entries), heavy metals (18.2%) and sodicity (15.7%). For EcM fungi, drought (16.0%), heavy metals (19.1%) and N and P deposition (15.4%) and, for ErM fungi, heavy metals (16.7%), N and P deposition (19.0%) and warming (16.7%) were the most commonly tested factors. In general, land use change (0.2%), UV‐B radiation and microplastic (0.03%) were the least covered factors. For artificial light at night, no experiment including mycorrhizal fungi could be found. For most global change factors, more data were available for lab studies, except for land use change, overexploitation, N and P deposition and ozone (Figs 1c, S2). The global change factors were primarily tested as single factors in 93.5% of all included database entries (Fig. S2), with similar patterns for AM, EcM and ErM fungi (Fig. 1d; AM: 94.1%, EcM: 90.1%, ErM: 85.7%). Only 6.5% of the database entries covered experiments with multiple global change factors and their effects on mycorrhizas: with two‐factor combinations in 5.9% and three‐factor combinations in 0.6% of the database entries (Fig. S2; Table S9). For EcM fungi, higher order factor interactions were relatively more often tested under field than lab conditions than AM or ErM fungi (Fig. 1d; Table S10). For AM fungi, elevated atmospheric carbon dioxide, drought, species invasion, N and P deposition and warming were tested most often in combination (Fig. 1e), with the highest number of occurrences found for drought and warming interaction treatments (16.0%) (Table S9). The pattern of the factor interaction network is driven by lab studies focusing on global change factors interacting with species invasion (Fig. S3). For EcM fungi, elevated atmospheric carbon dioxide, drought, N and P deposition and warming but not species invasion were detected most often. Elevated atmospheric carbon dioxide and warming were the most commonly tested interaction treatments (14.1%). For ErM fungi, the patterns were less pronounced due to the overall lower number of factor combinations reported. Repeating the original search in 2025 showed that the major trends are still robust. The majority of articles still report on the prominent topics, for example drought, heavy metals and sodicity. Underrepresented topics such as UV‐B radiation or artificial light at night were rarely covered in recent years (Table S11). An exception is the topic of microplastic. In the last 3 years, the publication numbers increased from 2 to 29, with 86% of the articles fulfilling the eligibility criteria. For the topic of ‘multiple factors’, 167 new articles could be retrieved with 35% of the articles fulfilling the eligibility criteria. Only one article testing a combination of three factors was found (Table S12).

### The arbuscular mycorrhiza evidence base

Of the 2884 articles matching our eligibility criteria, 2267 articles reported research on AM fungi.

**Fig. 2:**
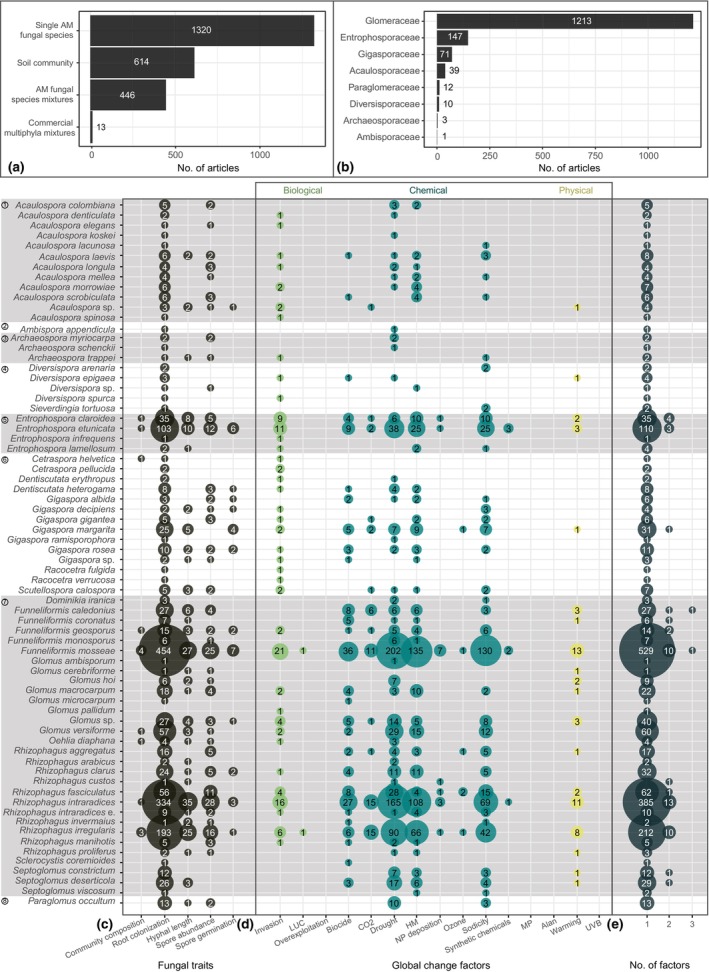
Overview of key data of the ‘arbuscular mycorrhiza’ database. (a) Bar plot on the number of occurrences of different inoculum types used: single arbuscular mycorrhizal (AM) fungal strains, whole soil communities, defined AM fungal species assemblages, commercially available multiphyla mixtures. (b) Bar plot on the number of occurrences of AM fungal families covered for single species inocula. (c) Bubble plot on the number of occurrences of measured AM fungal parameters (AM fungal community composition metrics, intraradical root colonization, extraradical hyphal length, spore abundance and spore germination capability) in the database. These data are only available for single species inocula. The AM fungal species are sorted alphabetically within their corresponding families (1: Acaulosporaceae, 2: Ambisporaceae, 3: Archaeosporaceae, 4: Diversisporaceae, 5: Entrophosporaceae, 6: Gigasporaceae, 7: Glomeraceae, 8: Paraglomeraceae). Rhizophagus intraradices e. means Rhizophagus intraradices emendation. (d) Balloon plot showing the number of occurrences of experiments for different global change factors tested. The factors were grouped into biological (species invasion, land use change and overexploitation), chemical (biocide, elevated atmospheric carbon dioxide, drought, heavy metals, nitrogen and phosphorus deposition, elevated tropospheric ozone, sodicity and synthetic chemicals), chemical–physical (microplastic) and physical (artificial light at night, warming and ultraviolet B radiation). (e) The number of cases for factors tested in combination is presented. For all data, only unique cases per article were counted (e.g. an article reporting on multiple single Glomeraceae strains provides only one data entry for the family‐level analysis).

The inoculum source used in the reported experiments fell into four categories (Fig. 2a): single AM fungal species (55.2% of the respective database entries), AM fungal species mixtures (18.6%), commercially available mixtures of species of different phyla including AM fungi (0.6%) and soil communities including AM fungi (25.7%). Field studies were dominated by soil community inocula (with 63.0% of respective field study entries) and lab studies by single species inocula (65% of respective lab study entries) (Table S13).

For single species inocula, species identifiers were reported. Those species could be assigned to eight families (Fig. 2b): Acaulosporaceae with 2.6%, Ambisporaceae with 0.07%, Archaeosporaceae with 0.2%, Diversisporaceae with 0.7%, Entrophosporaceae with 9.8%, Gigasporaceae with 4.7%, Glomeraceae with 81.1% and Paraglomeraceae with 0.8% of the respective literature. In total, 70 different AM fungal species were counted in the database (Fig. 2c). *Funneliformis mosseae* contributed to 29.1% of all related entries, *Rhizophagus intraradices* to 21.4% and *Rhizophagus irregularis* to 12.0%.

Among the many parameters measured to describe AM fungal performance, we focused here on abundance of intra‐ and extraradical mycelium and spores, spore germination capability and AM fungal community metrics, as these parameters are measured frequently and constantly through the research timeline. For single AM fungal species, intra‐ and extraradical mycelium measurements were the most common (82.8% and 8.2% of the respective database entries, respectively), while spore abundance (7.1%) and spore germination capability (1.4%) and AM fungal community metrics (0.6%) were measured the least across the different AM fungal species (Fig. 2c; Table S14). The most commonly tested global change factors were drought (34.4% of the respective database entries), heavy metals (25.4%) and sodicity (20.5%). In 97.3% of all entries, single factors were tested, while 2.6% reported two‐factor interactions and 0.08% reported three‐factor interactions (Fig. 2d; Table S15). There were no data for single species inocula on microplastics, artificial light at night or UV‐B radiation. For species belonging to the Entrophosporaceae, Gigasporaceae and Glomeraceae, data on many global change factors were reported, while for members of the Acaulosporaceae, Ambisporaceae, Archaeosporaceae and Diversisporaceae, only limited data were available (Fig. 2d). Higher order factor combinations were mainly found for Glomeraceae species (Fig. 2e).

## Discussion

Research on global change factor effects on mycorrhizal fungi is heterogeneously structured across the investigated parameters, leading to multiple knowledge clusters and gaps. Articles reporting on experiments on the topic of global change represent a small fraction of overall mycorrhizal research. However, this fraction increased in the past years, meaning the gap between the number of papers on mycorrhizal topics in general and the ones dealing with experimental evidence on global change is wide but narrowing in recent years. This could be a signature of shifting research interests, funding priorities or both. Learning about the responses of a key symbiosis to factors of global change is an important topic and should be further pursued by researchers and funders.

### Opportunities for data synthesis

Beyond this general trend, we found that global change themed experiments including mycorrhizas focus primarily on single factors (Mohan *et al*., 2014; Rillig *et al*., 2019), with only a few factors providing the majority of database entries, for example drought and heavy metals. The choice of factors is often dictated by logistics (e.g. expensive treatment facilities) or research interest and funding priorities (e.g. agricultural relevance). We identify promising knowledge clusters for future in‐depth analyses. For example, the research focus on drought or heavy metals effects on AM fungi provides sufficient data for meta‐analyses with narrow mechanism‐driven topics, or even umbrella reviews (Belbasis *et al*., 2022) for high‐level overviews of broader research questions. The focus on research in the past three years on the topic of microplastic opened a new opportunity for meta‐analysis on microplastic effects on plant and AM fungi.

### Knowledge gaps

Except for these factors, the evidence is rather sparse, especially for newly emerging factors of global change, for example microplastic (Leifheit *et al*., 2021). As a consequence of this single‐factor focus, we identified a knowledge gap on multiple factor interaction effects on mycorrhizas, irrespective of the mycorrhizal type. When factor interaction treatments were tested, data were clustered around specific factors, for example drought, warming, elevated atmospheric carbon dioxide and N and P deposition (Rillig *et al*., 2019). These constraints limit any attempt at quantitative data synthesis to pairwise factor interaction effects for a few global change factors. No data on more than three combined GC factors were detected, leaving a knowledge gap wide open for higher order factor interaction effects.

Additionally, these data are dominated by AM fungal studies (likely because herbaceous plants are often used in experiments), and hence, EcM and ErM fungi are strongly underrepresented (Read, 1991; Lekberg & Helgason, 2018). But even for AM fungi, data come from only a few Glomeraceae species (Koricheva *et al*., 2009; Augé *et al*., 2015; Lehmann & Rillig, 2025). Thus, the mycorrhizal evidence base is imbalanced toward AM fungal‐dominated ecosystems (Lekberg & Helgason, 2018; Soudzilovskaia *et al*., 2019) and response variables derived from a small pool of species. Such limitations will reduce our inference capability for phylogenetically informed analysis.

Additionally, the evidence base comprises primarily lab studies (Lekberg & Helgason, 2018), limiting the generalizability of future in‐depth analyses when it comes to real‐world conditions.

Although publications from all five continents are available, we identified clear geographical research hotspots (Collen *et al*., 2008; Mohan *et al*., 2014). Major contributions were provided by the United States, Europe and China, with countries from Africa, South America and Australia lagging behind. The dominance of specific geographical regions is a well‐known issue in ecology with clear consequences (Martin *et al*., 2012) for the generalizability of findings.

### Research recommendations

In order to fill the identified knowledge gaps, future research could consider: testing multiple global change factors with new experimental designs (Rillig *et al*., 2019; Bi *et al*., 2024) to evaluate the effects of factor‐level richness or dissimilarity on mycorrhizas; new emergent global change factors for risk assessment for mycorrhizal fungal species (Rillig *et al*., 2024; Wang *et al*., 2024); specifically EcM and ErM fungal species for their traits and stress alleviation capabilities to reduce the mycorrhizal type imbalance in the overall literature; and AM fungal species beyond the popular Glomeraceae strains to increase phylogenetic coverage of trait data.

These research opportunities will provide urgently needed new insights into global change effects on mycorrhiza and consequences for plant hosts.

## Competing interests

None declared.

## Author contributions

AL initiated the work and wrote the first draft. AL conducted the literature search. AL, RR, AS, KE, KH, NC and NQ screened the articles. AL and EFL were the cross‐checkers and trainers of the screeners. AL and SM built and finalized the data table. AL conducted the data analysis. AL designed the conceptual figures. AL and MCR contributed to the writing of the manuscript, with additional contributions from BT.

## Supporting information


**Fig. S1** RepOrting standards for Systematic Evidence Synthesis flow diagram.
**Fig. S2** Number of occurrences for specific database parameters.
**Fig. S3** Global change factor combinations tested in experiments focusing on arbuscular mycorrhiza, ectomycorrhiza and ericoid mycorrhiza in field and lab studies.
**Methods S1** Supplementary methods.
**Notes S1** Reference list of database articles.
**Table S1** Search strings for targeting reviews used to collect global change factor and mycorrhiza terms for preliminary and final search strings.
**Table S2** Top 10 research areas, their number of hits, their percent contribution to the overall amount of hits and number.
**Table S3** Global change factor‐specific preliminary search strings.
**Table S4** Final search strings for topic searches.
**Table S5** Eligibility criteria.
**Table S6** Coding of general system information.
**Table S7** Quality assessment.
**Table S8** Search outcomes for search results retrieved until 2021 and since 2021.
**Table S9** Most common factor combinations for mycorrhiza types.
**Table S10** Number of occurrences for mycorrhiza fungi and factor combinations.
**Table S11** Comparison of ‘till 2021’ and ‘since 2021’ searches.
**Table S12** Comparison of ‘till 2021’ and ‘since 2021’ searches for global change factors.
**Table S13** Cases of occurrence for inoculum types.
**Table S14** Cases of occurrence for traits measured in Arbuscular mycorrhizal fungi.
**Table S15** Cases of occurrence for global change factors and factor combinations.
Please note: Wiley is not responsible for the content or functionality of any Supporting Information supplied by the authors. Any queries (other than missing material) should be directed to the *New Phytologist* Central Office.

## Data Availability

The data that support the findings of this study are available in the Supporting Information (Notes S1) of this article.

## References

[nph70452-bib-0001] Anthony MA , Bender SF , van der Heijden MGA . 2023. Enumerating soil biodiversity. Proceedings of the National Academy of Sciences, USA 120: e2304663120. 10.1073/pnas.2304663120 PMC10437432 37549278

[nph70452-bib-0002] Augé RM , Toler HD , Saxton AM . 2014. Arbuscular mycorrhizal symbiosis and osmotic adjustment in response to NaCl stress: a meta‐analysis. Frontiers in Plant Science 5: 562. 25368626 10.3389/fpls.2014.00562 PMC4201091

[nph70452-bib-0003] Augé RM , Toler HD , Saxton AM . 2015. Arbuscular mycorrhizal symbiosis alters stomatal conductance of host plants more under drought than under amply watered conditions: a meta‐analysis. Mycorrhiza 25: 13–24. 24831020 10.1007/s00572-014-0585-4

[nph70452-bib-0004] Bardgett RD , van der Putten WH . 2014. Belowground biodiversity and ecosystem functioning. Nature 515: 505–511. 25428498 10.1038/nature13855

[nph70452-bib-0005] Belbasis L , Bellou V , Ioannidis JPA . 2022. Conducting umbrella reviews. BMJ Medicine 1: e000071. 36936579 10.1136/bmjmed-2021-000071 PMC9951359

[nph70452-bib-0006] Bi M , Li H , Meidl P , Zhu Y , Ryo M , Rillig MC . 2024. Number and dissimilarity of global change factors influences soil properties and functions. Nature Communications 15: 8188. 10.1038/s41467-024-52511-2 PMC11410830 39294171

[nph70452-bib-0007] Brundrett MC , Tedersoo L . 2018. Evolutionary history of mycorrhizal symbioses and global host plant diversity. New Phytologist 220: 1108–1115. 29355963 10.1111/nph.14976

[nph70452-bib-0008] Bueno CG , Meng Y , Neuenkamp L . 2022. How can mycorrhizal symbiosis mediate multiple abiotic stresses in woody plants? Flora 295: 152146.

[nph70452-bib-0009] Collen B , Ram M , Zamin T , McRae L . 2008. The tropical biodiversity data gap: addressing disparity in global monitoring. Tropical Conservation Science 1: 75–88.

[nph70452-bib-0010] Csardi G , Nepusz T . 2005. The igraph software package for complex network research. InterJournal Complex Systems 1695: 458.

[nph70452-bib-0011] Fitter AH , Gilligan CA , Hollingworth K , Kleczkowski A , Twyman RM , Pitchford JW , programme TmotNsb . 2005. Biodiversity and ecosystem function in soil. Functional Ecology 19: 369–377.

[nph70452-bib-0012] Haddaway NR , Macura B , Whaley P , Pullin AS . 2018. ROSES RepOrting standards for Systematic Evidence Syntheses: pro forma, flow‐diagram and descriptive summary of the plan and conduct of environmental systematic reviews and systematic maps. Environmental Evidence 7: 1–7.

[nph70452-bib-0013] Kassambara A . 2023. ggpubr: ‘ggplot2’ based publication ready plots. [WWW document] URL https://github.com/kassambara/ggpubr

[nph70452-bib-0014] Kivlin SN , Emery SM , Rudgers JA . 2013. Fungal symbionts alter plant responses to global change. American Journal of Botany 100: 1445–1457. 23757444 10.3732/ajb.1200558

[nph70452-bib-0015] Koricheva J , Gange AC , Jones T . 2009. Effects of mycorrhizal fungi on insect herbivores: a meta‐analysis. Ecology 90: 2088–2097. 19739371 10.1890/08-1555.1

[nph70452-bib-0016] Lehmann A , Rillig MC . 2025. Systematic mapping of experimental approaches to studying common mycorrhizal networks in arbuscular mycorrhiza. Plants, People, Planet 24: 458.

[nph70452-bib-0017] Leifheit EF , Lehmann A , Rillig MC . 2021. Potential effects of microplastic on Arbuscular mycorrhizal fungi. Frontiers in Plant Science 12: 626709. 33597964 10.3389/fpls.2021.626709 PMC7882630

[nph70452-bib-0018] Lekberg Y , Helgason T . 2018. In situ mycorrhizal function – knowledge gaps and future directions. New Phytologist 220: 957–962. 29436724 10.1111/nph.15064

[nph70452-bib-0019] Martin LJ , Blossey B , Ellis E . 2012. Mapping where ecologists work: biases in the global distribution of terrestrial ecological observations. Frontiers in Ecology and the Environment 10: 195–201.

[nph70452-bib-0020] Mohan JE , Cowden CC , Baas P , Dawadi A , Frankson PT , Helmick K , Hughes E , Khan S , Lang A , Machmuller M *et al*. 2014. Mycorrhizal fungi mediation of terrestrial ecosystem responses to global change: mini‐review. Fungal Ecology 10: 3–19.

[nph70452-bib-0021] Orr JA , Macaulay SJ , Mordente A , Burgess B , Albini D , Hunn JG , Restrepo‐Sulez K , Wilson R , Schechner A , Robertson AM *et al*. 2024. Studying interactions among anthropogenic stressors in freshwater ecosystems: a systematic review of 2396 multiple‐stressor experiments. Ecology Letters 27: e14463. 38924275 10.1111/ele.14463

[nph70452-bib-0022] Pebesma E . 2018. Simple features for R: standardized support for spatial vector data. The R Journal 10: 439–446.

[nph70452-bib-0023] Piggott JJ , Townsend CR , Matthaei CD . 2015. Reconceptualizing synergism and antagonism among multiple stressors. Ecology and Evolution 5: 1538–1547. 25897392 10.1002/ece3.1465 PMC4395182

[nph70452-bib-0024] Pirotta E , Thomas L , Costa DP , Hall AJ , Harris CM , Harwood J , Kraus SD , Miller PJO , Moore MJ , Photopoulou T *et al*. 2022. Understanding the combined effects of multiple stressors: a new perspective on a longstanding challenge. Science of the Total Environment 821: 153322. 35074373 10.1016/j.scitotenv.2022.153322

[nph70452-bib-0025] R Core Team . 2021. R: A language and environment for statistical computing. Vienna, Austria: R Foundation for Statistical Computing.

[nph70452-bib-0026] Read DJ . 1991. Mycorrhizas in ecosystems. Experientia 47: 376–391.

[nph70452-bib-0027] Rillig MC , Li C , Jin LN , Kim SW . 2024. Understanding the soil plastisphere and its environmental impacts. One Earth 7: 2095–2098.

[nph70452-bib-0028] Rillig MC , Ryo M , Lehmann A . 2021. Classifying human influences on terrestrial ecosystems. Global Change Biology 27: 2273–2278. 33660892 10.1111/gcb.15577

[nph70452-bib-0029] Rillig MC , Ryo M , Lehmann A , Aguilar‐Trigueros CA , Buchert S , Wulf A , Iwasaki A , Roy J , Yang G . 2019. The role of multiple global change factors in driving soil functions and microbial biodiversity. Science 366: 886–890. 31727838 10.1126/science.aay2832 PMC6941939

[nph70452-bib-0030] Schüßler A . 2024. Glomeromycota: species list. [WWW document] URL http://schuessler.userweb.mwn.de/amphylo [accessed 7 July 2024].

[nph70452-bib-0031] Soudzilovskaia NA , van Bodegom PM , Terrer C , Zelfde M , McCallum I , McCormack LM , Fisher JB , Brundrett MC , de Sá NC , Tedersoo L . 2019. Global mycorrhizal plant distribution linked to terrestrial carbon stocks. Nature Communications 10: 5077. 10.1038/s41467-019-13019-2 PMC6838125 31700000

[nph70452-bib-0032] Speißer B , Wilschut RA , van Kleunen M . 2022. Number of simultaneously acting global change factors affects composition, diversity and productivity of grassland plant communities. Nature Communications 13: 7811. 10.1038/s41467-022-35473-1 PMC9763497 36535931

[nph70452-bib-0033] Tang B , Man J , Lehmann A , Rillig MC . 2023. Arbuscular mycorrhizal fungi benefit plants in response to major global change factors. Ecology Letters 26: 2087–2097. 37794719 10.1111/ele.14320

[nph70452-bib-0034] Tedersoo L , Bahram M . 2019. Mycorrhizal types differ in ecophysiology and alter plant nutrition and soil processes. Biological Reviews 94: 1857–1880. 31270944 10.1111/brv.12538

[nph70452-bib-0035] Wang F , Xiang L , Sze‐Yin Leung K , Elsner M , Zhang Y , Guo Y , Pan B , Sun H , An T , Ying G *et al*. 2024. Emerging contaminants: A one health perspective. The Innovation 5: 100612. 38756954 10.1016/j.xinn.2024.100612 PMC11096751

[nph70452-bib-0036] Wickham H . 2016. ggplot2: elegant graphics for data analysis. New York, NY, USA: Springer‐Verlag.

[nph70452-bib-0037] Zandalinas SI , Sengupta S , Fritschi FB , Azad RK , Nechushtai R , Mittler R . 2021. The impact of multifactorial stress combination on plant growth and survival. New Phytologist 230: 1034–1048. 33496342 10.1111/nph.17232 PMC8048544

[nph70452-bib-0038] Zhou L , Zhou X , He Y , Fu Y , Du Z , Lu M , Sun X , Li C , Lu C , Liu R *et al*. 2022. Global systematic review with meta‐analysis shows that warming effects on terrestrial plant biomass allocation are influenced by precipitation and mycorrhizal association. Nature Communications 13: 4914. 10.1038/s41467-022-32671-9 PMC9392739 35987902

