# Supplementary material for: Research landscape of experiments on global change effects on mycorrhizas
Source: New Phytol. 2025 Aug 8;248(4):1612–9. doi: 10.1111/nph.70452 (PMC12529039; doi:10.1111/nph.70452)
Supplement: Supplementary file 1 — Fig. S1 RepOrting standards for Systematic Evidence Synthesis flow diagram. Fig. S2 Number of occurrences for specific database parameters. Fig. S3 Global change factor combinations tested in experiments focusing on arbuscular mycorrhiza, ectomycorrhiza and ericoid mycorrhiza in field and lab studies. Methods S1 Supplementary methods. Notes S1 Reference list of database articles. Table S1 Search strings for targeting reviews used to collect global change factor and mycorrhiza terms for preliminary and final search strings. Table S2 Top 10 research areas, their number of hits, their percent contribution to the overall amount of hits and number. Table S3 Global change factor‐specific preliminary search strings. Table S4 Final search strings for topic searches. Table S5 Eligibility criteria. Table S6 Coding of general system information. Table S7 Quality assessment. Table S8 Search outcomes for search results retrieved until 2021 and since 2021. Table S9 Most common factor combinations for mycorrhiza types. Table S10 Number of occurrences for mycorrhiza fungi and factor combinations. Table S11 Comparison of ‘till 2021’ and ‘since 2021’ searches. Table S12 Comparison of ‘till 2021’ and ‘since 2021’ searches for global change factors. Table S13 Cases of occurrence for inoculum types. Table S14 Cases of occurrence for traits measured in Arbuscular mycorrhizal fungi. Table S15 Cases of occurrence for global change factors and factor combinations. Please note: Wiley is not responsible for the content or functionality of any Supporting Information supplied by the authors. Any queries (other than missing material) should be directed to the New Phytologist Central Office. [file NPH-248-1612-s001.pdf]

New Phytologist

***New Phytologist* Supporting Information**

Article title: Global research landscape of global change effects on mycorrhizas
Authors: Anika Lehmann; Bo Tang; Rebecca Rongstock; Alexa Sommerburg; Natalja Chramova; Kevser Ergül; Katharina Heydebreck; Nasrin Quiram; Stefanie Maaß; Eva F. Leifheit; Matthias C. Rillig
Article acceptance date: 17 July 2025

The following Supporting Information is available for this article:

Methods S1 Supplementary methods

Fig. S1 Roses flow diagram.

Fig. S2 Number of occurrences for specific database parameters

Fig. S3 Global change factor combinations tested in experiments focusing on arbuscular mycorrhiza (AM), ectomycorrhiza (EcM) and ericoid mycorrhiza (ErM) in field and lab studies.
Table S1 Search strings for targeting reviews used to collect global change factor and mycorrhiza terms for preliminary and final search strings

Table S2 Top 10 research areas, their number of hits, their percent contribution to the overall amount of hits and number

Table S3 Global change factors specific preliminary search strings

Table S4 The final search strings for topic searches

Table S5 Eligibility criteria

Table S6 Coding of general system information

Table S7 Quality assessment

Table S8 Search outcomes for search results retrieved until 2021 and since 2021

Table S9 Most common factor combinations for mycorrhiza types

Table S10 Number of occurrences for mycorrhiza fungi and factor combinations

Table S11 Comparison of “till 2021” and “since 2021” searches

Table S12 Comparison of “till 2021” and “since 2021” searches for global change factors

New Phytologist

Table S13 Cases of occurrence for inoculum types

Table S14 Cases of occurrence for traits measured in arbuscular mycorrhizal fungi

Table S15 Cases of occurrence for global change factors and factor combinations

Notes S1 Reference list of database articles

Methods S1 Supplementary methods

1. Search string development

    a. Global change factor terms

First, we build a list of target global change (GC) factors. For this, we run a topic search for relevant reviews in the Web of Science Core collection (WoS CC) database (in September 2020 with 26,799 hits; search string 1). We further fine-tuned our search outcome by focusing on the top 10 research areas (sorted by number of hits; see Table S1). From each research area we collected the top 100 articles sorted by a) times cited, b) usage count and c) relevance (search term ranking based) (WoS-help page1 https://images.webofknowledge.com/WOKRS533JR18/help/WOS/hs_sort_options.html). The bibliometric data of the resulting 1842 reviews (unique articles, no duplicates) were exported to bibliometrix (Aria & Cuccurullo, 2017) for an analysis of the most frequent words in a) titles, b) abstracts, c) author keywords and d) keywords plus (WoS-help page2 https://support.clarivate.com/ScientificandAcademicResearch/s/article/KeyWords-Plus-generation-creation-and-changes?language=en_US). The analysis extracted 34,004 unique terms. We screened terms with an occurrence greater than 2 for the category "title", "author keywords" and "keywords plus" and for "abstract" until occurrence greater than 10 because "abstract" provided up to four times more terms than any of the other categories. From the resulting term collection, we created a list of potential GC factors focusing on chemical, physical and biological factors (Rillig et al., 2021). We also collected additional words e.g., for term exclusions (e.g., "cooling") or condition specification for the GC factors (e.g., "elevated"); these supporter terms were specified for each GC factor. The list was evaluated by expert knowledge (i.e., lab members). The final list comprised the following 15 GC factors: biocides (e.g. antibiotics, fungicides, herbicides), elevated atmospheric carbon dioxide, warming (including heat waves or pulses), drought, artificial light at night, nitrogen and phosphorus deposition (also including eutrophication), heavy metal (focusing on zinc, copper, iron, manganese, cadmium, lead, chromium, mercury, and arsenic), sodicity (contamination by sodium ions), species invasion, elevated tropospheric ozone, land use change (including land degradation, conversion and fragmentation, not land restoration or conservation), ultraviolet B radiation,

synthetic chemicals (focusing on surfactants and per- and polyfluoroalkyl substances), microplastics, overexploitation (including overharvesting, overgrazing, clear-cutting).

The GC factors can be represented by different concepts; i.e., the words warming, increased temperature or heat pulse can be united under the umbrella of the same GC factor - namely global warming. To identify terms representing different concepts of the GC factors, we ran preliminary searches for each GC factor (preliminary search string 1 to 17). For several preliminary search strings, we yielded critically high hit numbers which can exacerbate the search term development. Word analysis can be misleading since the article collection could be unintendedly biased by a dominant or multiple mismatching topic cluster (e.g., metallurgy, surgery, architecture, physics also the interest is in e.g., ecology). To solve this issue, we investigated the WoS research areas of the articles obtained by the preliminary searches to identify mismatching areas for exclusion. For this we checked the 153 WoS research areas for the 15 GC factor searches. We did not evaluate WoS research areas with less than 300 hits. We checked the titles and abstracts of the top 10 articles sorted by relevance. Only when all 10 most relevant articles were not suitable (not an experiment including at least one of the targeted GC factors), the WoS research area was added to the GC factor-specific exclusion list. We combined the excluded WoS research areas by the NOT-boolean operator with our preliminary search strings. The resulting articles were sorted by a) relevance, b) times cited and c) usage count. The top 1000 articles of each sorting for the top 3 WoS research areas were exported to bibliometrix for word analysis. We checked the top 300 hits for titles, and author keywords and keywords plus and the top 500 hits for abstracts to collect terms representing different GC factor concepts and additional terms to further specify the concept (e.g., increased temperature for global warming, or nutrient enrichment for N and P deposition); these we call supporter terms.

The GC factor and their supporter terms were combined by the AND-boolean operator. The updated search strings were further fine-tuned by exchanging the AND- by the NEAR/x-boolean operator when possible. We evaluated if we excluded potential matching articles by this step by checking the top 10 most relevant articles of the excluded subset. If we identified potential matches in the exclusion subset, we undid that step to not lose promising articles.

b. Mycorrhiza terms

For the mycorrhiza terms, we followed a similar strategy as for the GC factors. We ran a topic search in WoS-CC for reviews (December 2020 with 29,717 hits; search string 2) and the retrieved articles were sorted by a) times cited, b) usage count and c) relevance. The top 1000 articles of each sorting were collected and the bibliometric data of all unique 2569 articles was exported to bibliometrix. The data were checked for potential "mycorrhiza" terms in a) titles, b) abstracts, c) author keywords and d) keywords plus for terms with an occurrence greater than 2. The resulting list was broadened by the mycorrhizal types covered in (Smith & Read, 2008) which were not or too rarely covered by reviews to appear in our term analysis. The final list comprised endo-, ecto- and ectendomycorrhizas (arbuscular, arbutoid, ericoid, monotropoid and orchid mycorrhiza).

c. Search string modules

The final search strings comprised four term modules: a) the global change factor terms, b) the mycorrhiza terms, c) the WoS research area exclusions and d) the review and meta-analysis exclusions. Each search string for the 15 global change factors (Table S3) was of the general syntax:

"GC factor terms AND mycorrhiza terms NOT research area terms NOT reviews and meta-analysis". Detailed information on search string development can be found in the supplementary materials. The "multiple" search string was dedicated to specifically target articles testing multiple GC factor treatments. These studies do not mention which GC factors they covered in title, abstract or keywords but state that they measured multiple factors.

2. Quality assessment of search strings

To evaluate the performance of global change factor specific search strings at the end of the screening procedure, we added a tag to each article denoting by which search string or search strings this article was found. We used these GC factor tags which we assigned to each article (see section "search and database") to check how many articles matching our eligibility criteria

could be retrieved by each search string. We also used the tagged articles to test how efficiently the respective search string could find articles for its specific GC factor; i.e., for example, how many matching articles targeting the GC factor warming could be found by the search strings covering the concept of warming (Table S4 and S5).

3. Detailed information on screening, coding and database construction

For the collected AM fungal parameters, we noted presence of measurements focusing on the intraradical structures colonizing the host roots (e.g., percent or specific root length colonized), extraradical structures present in the soil (e.g., hyphal length, hyphal density), and the abundance and germination capability of spores.

Of the overall 7107 screened articles 4107 were identified as not fulfilling our eligibility criteria and 15 articles were not accessible. Of the articles matching our eligibility criteria, 117 articles used biocides not as global change factor treatments but as tools to initiate a mycorrhizal treatment for field experiments. We excluded these studies when the biocide was the only applied global change factor from our list. Thus, we excluded 101 articles. Thus, our final database comprised 2884 articles providing 3006 data rows (Figure S1). One article can provide multiple database entries when reporting suitable data for different mycorrhizal types and our pre-defined general system parameters (setting, sterility). The global map and temporal trend analyses (Figure 1a and 1b) included only one database entry per article. Also, 0.5% of the articles did not specify the setting of their experiments. These unspecified cases were excluded for all remaining analyses.

It is noteworthy that some articles included in our database are derived from long-term ecological research programs or other long-term experiments resulting in multiple publications covering the same experiment.

We also want to highlight some limitations concerning the factor “N and/or P deposition”. We state the eligibility criteria in Table S5. To distinguish studies testing for the global change factor N and/or P deposition studies from those testing fertilizers composing N and/or P, we screened

the abstracts and introductions of articles to evaluate if the authors set their studies in the context of N and/or P deposition or eutrophication. However, as reported by Bebber (2021), N and/or P concentration applied in global change themed studies can exceed realistic N and/or P deposition concentrations. We could not consider the amount of deposited N and/or P in studies fulfilling our eligibility criteria for N and/or P deposition experiments. Thus, our database contains articles testing N and/or P concentrations beyond 10 mg/kg as N and/or P deposition treatments.

Fig. S1 Roses flow diagram

Roses flow diagram (https://estech.shinyapps.io/roses_flowchart/) for the two conducted searches done in January 2021 and 2022.

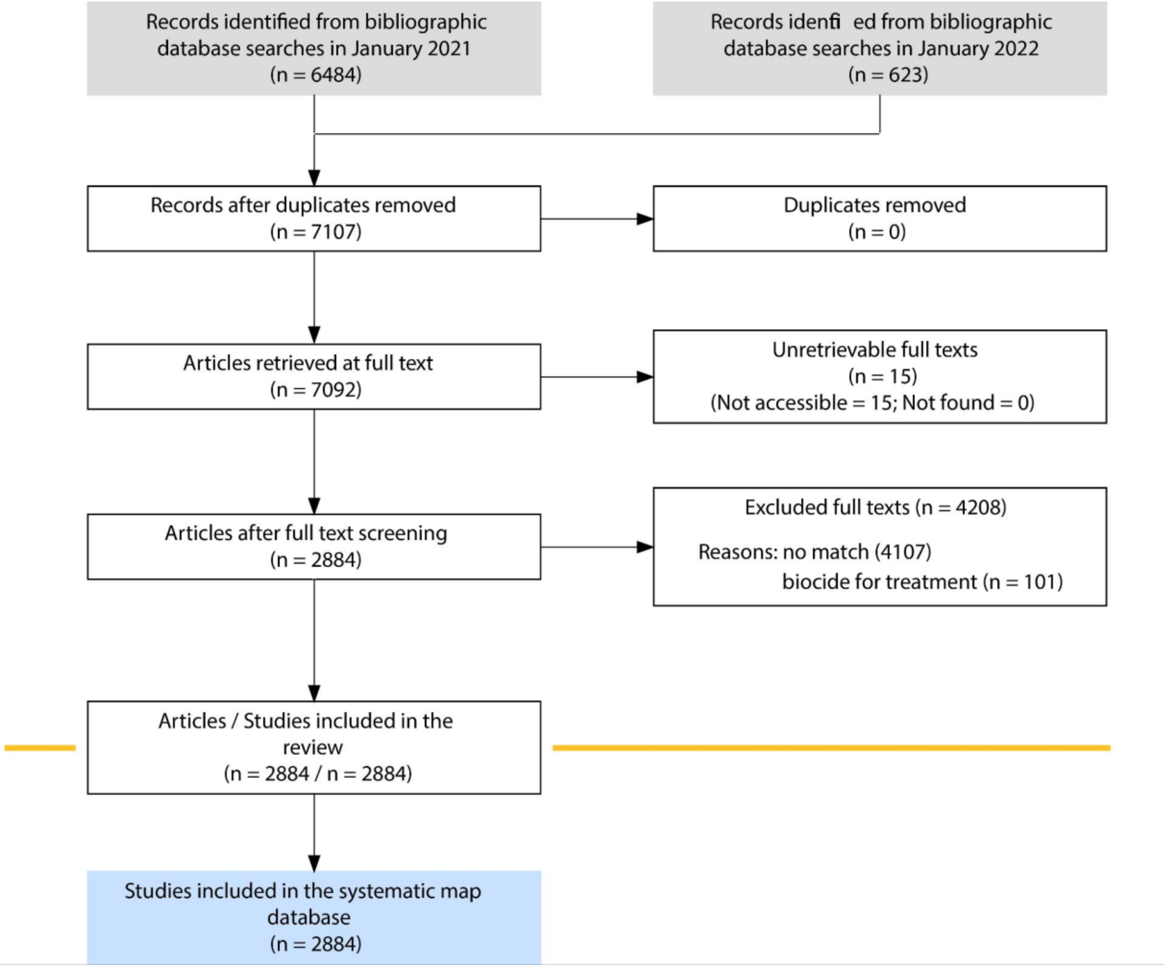

Fig. S2 Number of occurrences for specific database parameters.

(A) cases for the different mycorrhizal fungi (arbuscular mycorrhizal (AM) fungi, ectomycorrhizal (EcM) fungi, ericoid mycorrhizal (ErM) fungi, orchid mycorrhizal fungi (OrM), arbutoid mycorrhizal fungi (AbM), ectendomycorrhizal fungi (EeM)), (B) cases for three experimental design conditions (Was mycorrhiza tested as a treatment? What was the setting? Was the growth substrate sterilized?), (C) cases for the 15 global change factors investigated in this database (drought, heavy metals, sodicity, biocide, species invasion, elevated atmospheric carbon dioxide, nitrogen and phosphorus deposition, warming, elevated tropospheric ozone, overexploitation, synthetic chemicals, land use change, ultraviolet B radiation, microplastic and artificial light at night). (D) Cases for single, two-factor and three-factor combinations of global change factor treatments.
Data are sorted in decreasing order and include all mycorrhizal fungi types. Values on top of each bar represent the exact number of occurrences.

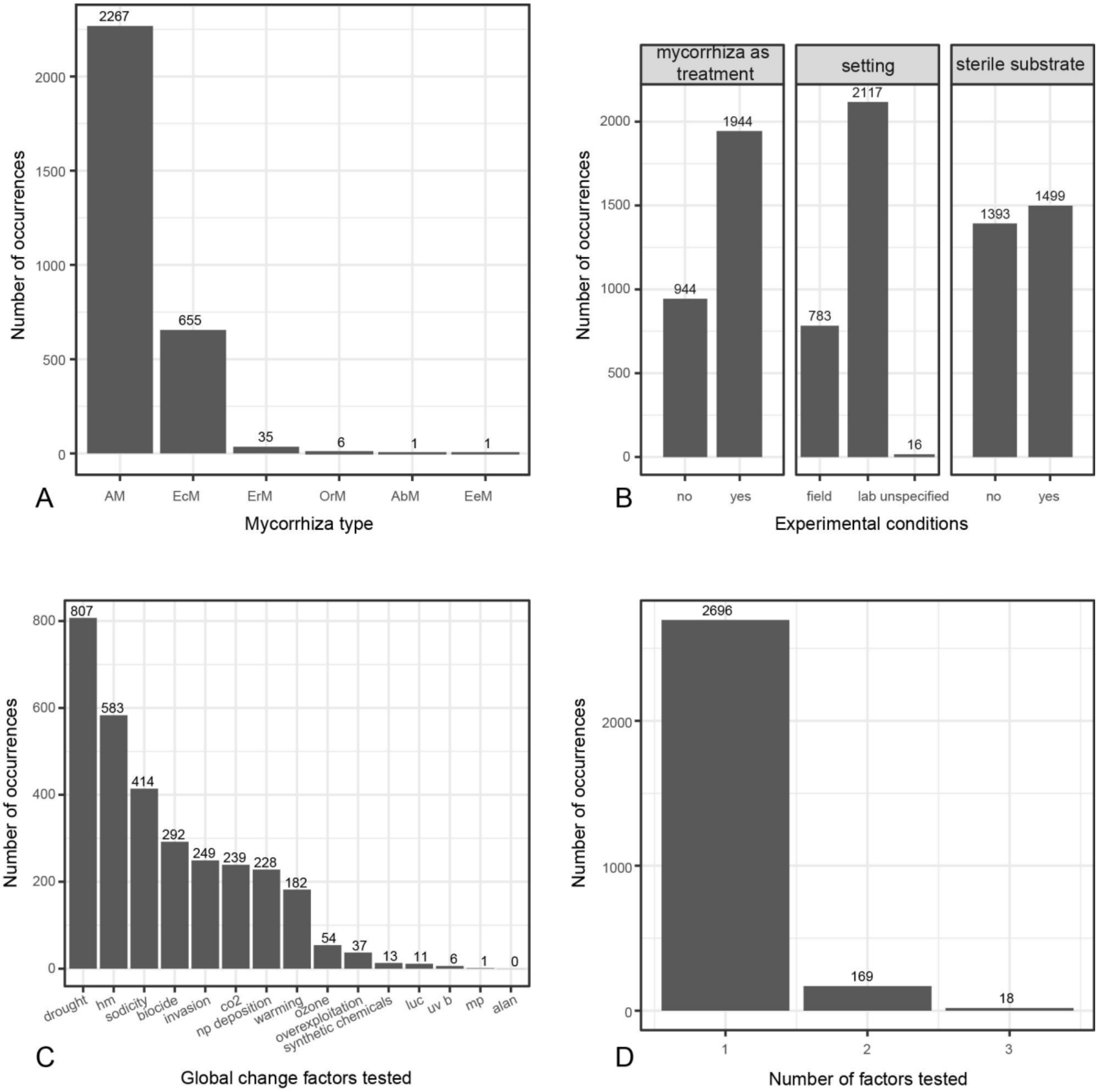

Fig. S3 Global change factor combinations tested in experiments focusing on arbuscular mycorrhiza (AM), ectomycorrhiza (EcM) and ericoid mycorrhiza (ErM) in field and lab studies. The global change factors are: species invasion, land use change, overexploitation, biocide, elevated atmospheric carbon dioxide, drought, heavy metals, N and P deposition, elevated tropospheric ozone, sodicity, synthetic chemicals, microplastic, artificial light at night, warming and ultraviolet B radiation.

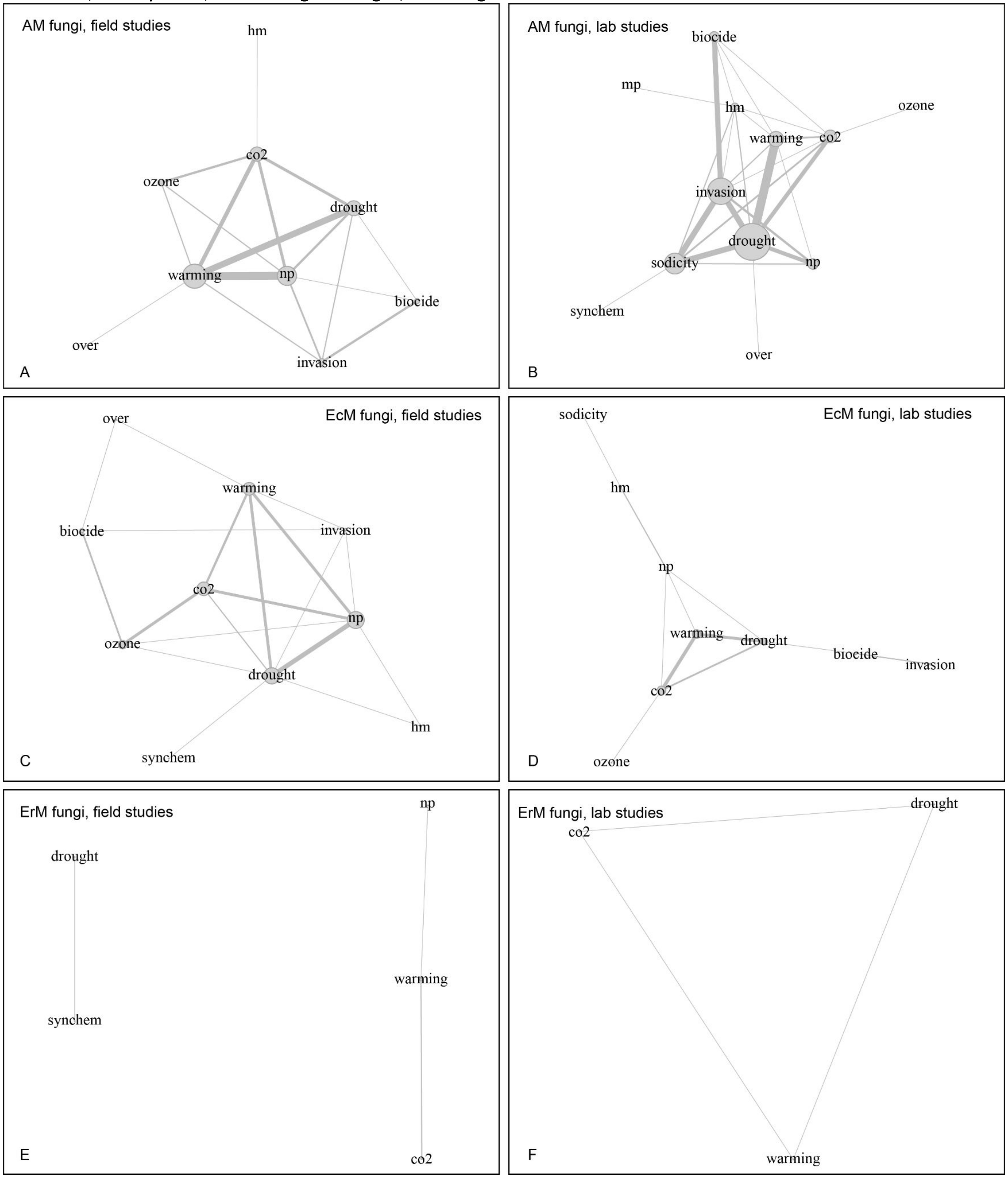

Table S1 Search strings for targeting reviews used to collect global change factor and mycorrhiza terms for preliminary and final search strings

See also Table S3 and Table S4. We used topic searches (TS=), excluded specific subject areas (SU=) and focused on the document type "Review".

search string number	search string
search string 1 - global change factor reviews	TS=((global change OR climate change OR environmental change) NOT SU=(chem* OR biochem* OR geochem* OR astrochem* OR engineering OR "material*" OR medic* OR neuro* OR health OR technolog* OR biotechnolog* OR physic* OR biophysic* OR geophysic* OR astrophysic* OR mechanic* OR spectroscopy OR optics)) ; document type = Review
search string 2 - mycorrhiza reviews	TS=(mycorrhiza*); document type = Review

Table S2 Top 10 research areas, their number of hits, their percent contribution to the overall amount of hits and number

Top 10 research areas, their number of hits, their percent contribution to the overall amount of hits and number of unique (duplicates got eliminated by the Web of Science platform automatically) articles per research area for global change factor and mycorrhiza review searches (Table S1).

Research Area	hits	% of overall 26799 hits	new articles to add to list; eliminated duplicates
Environmental Sciences Ecology	7674	28.64	242
Plant Sciences	2314	8.64	221
Agriculture	1654	6.17	232
Geology	1747	6.52	215
Meteorology Atmospheric Sciences	1331	4.97	192
Marine Freshwater Biology	1191	4.44	207
Biodiversity Conservation	1077	4.02	131
Microbiology	968	3.61	171
Genetics Heredity	927	3.46	161
Evolutionary Biology	926	3.46	70

Table S3 Global change factors specific preliminary search strings

Global change factors specific preliminary search strings (topic searchers, TS=) used in search string development. The global change factors are: species invasion, land use change, overexploitation, biocide, elevated atmospheric carbon dioxide, drought, heavy metals, N and P deposition, elevated tropospheric ozone, sodicity, synthetic chemicals, microplastic, artificial light at night, warming and ultraviolet B radiation.

**preliminary search string number**	**global change factor**	**search string**	**hits**
preliminary search string 1	warming	TS=("warming" OR "temperature" OR "temperatures" OR "heat")	3.9 Mio
preliminary search string 2	drought	TS=(water OR drought OR precipitation OR droughts OR drying OR "soil moisture" OR "drought stress" OR "water stress")	3.3 Mio
preliminary search string 3	LUC	TS=(("land use" OR "land-use" OR *land* OR patch* OR loss* OR fragment* OR degrad* OR connect* OR corridor$) AND (conver* OR transform* OR transi* OR change$ OR habitat* OR *land* OR forest$ OR sea$))	3.2 Mio
preliminary search string 4	ALAN	TS=((((light* OR bright* OR *glow* OR illumination OR LED) AND (night* OR nocturnal)) OR "light pollution$")))	34,000
preliminary search string 5	CO2	TS=("CO2" OR "atmospheric CO2" OR "carbon dioxide" OR "atmospheric carbon dioxide" OR "ocean acidification")	0.5 Mio
preliminary search string 6	MP	TS=(micro-plastic* OR microplastic*)	4,600
preliminary search string 7	invasion	TS=(exotic OR alien OR inva* OR *native OR introduced OR *indigenous)	3.0 Mio
preliminary search string 8	ozone	TS=("O-3" OR "O3" OR ozone)	152,00 0
preliminary search string 9	biocides	TS =((biocide$ OR insecticide$ OR bacteriocide$ OR fungicide$ OR herbicide$ OR acaricide$ OR nematocide$ OR pesticide$ OR antifungal OR antibacterial OR antimycotic$ OR antibiotic$ OR antimicrobial OR antifoul* OR anti-foul*)	0.7 Mio
preliminary search string 10	synChem1	TS=(polyfluor* OR perfluor* OR fluorosurfactant$ or "fluorinated surfactant$")	52,000
preliminary search string 11	synChem2	TS=(surfactant$ OR "surface active agent$" OR "surface active chemical$" OR "synthetic* detergent$" OR "soil conditioner$" OR "soil washing")	0.2 Mio
preliminary search string 12	HM	TS =("heavy metal$" OR "heavy-metal$" OR "Zn" OR zinc OR "Cu" OR copper OR iron OR "Fe" OR manganese OR "Mn" OR "Cd" OR cadmium OR "Pb" OR lead OR "Cr" OR chro mium OR chromium(vi) OR Cr(vi) OR "hexavalent chromium" OR "hexavalent Cr" OR "Hg" OR Mercury OR arsenic)	5.0 Mio
preliminary search string 13	overexploitatio n	TS=(overexploitation OR overexploited OR over-used OR overused OR overuse OR over-use OR overutilization OR overutilisation OR overutilized)	21,000
preliminary search string 14	NP depositon	TS=(fertilizer$ OR fertilization OR phosphorus OR phosphate OR phosphite OR nitrogen OR urea OR ammonium OR nutri* OR eutrophication)	1.8 Mio

preliminary search string 15	sodicity	TS=(salinity OR salinities OR saline OR sodic OR sodicity OR sodication OR salinized OR salinization OR salt OR salts OR NaCl OR sodium OR "ESP" OR "exchangeable sodium percentages" OR "exchangeable sodium percentage" OR "SAR" OR "sodium adsorption ratio" OR "sodium adsorption ratios" OR "electrical conductivity" OR "EC")	1.4 Mio
preliminary search string 16	UVB	TS=("UV radiation" OR "UV-radiation" OR "UV-B" OR "UVB" "UV B" OR "(UVB)" OR "(UV-B)" OR ultraviolet-B OR ultraviolet-radiation OR "ultraviolet radiation" OR midultraviolet OR "stratospheric ozone reduction" OR "Stratospheric ozone" OR "ozone depletion")	56,000
preliminary search string 17	"multiple" factors	TS=((("multiple" OR "multifactor*" OR "multi-driver$" OR "multi-factor*" OR "multidriver$" OR "mixture$" OR "combination$" OR combined OR "sequential" OR simultaneous*) AND ("change" OR "changes" OR "changing" OR stressor$ OR "driver$", OR "factor$" OR pressure$ OR risk$))	2.0 Mio

Table S4 The final search strings for topic searches

The final search strings for topic searches (TS=) in Web of Science - Core Collection which were used to detect research articles on experiments on mycorrhizal fungi and global change factor effects (hit numbers for January 2021 search). SU means "subject area". The global change factors are: species invasion, land use change, overexploitation, biocide, elevated atmospheric carbon dioxide, drought, heavy metals, N and P deposition, elevated tropospheric ozone, sodicity, synthetic chemicals, microplastic, artificial light at night, warming and ultraviolet B radiation.

GC factor search string		GCF search string		Mycorrhiza search string		SUs to exclude	exclude reviews and MAs	hits
Biocides	TS=(	("biocide" OR "biocides" OR "insecticide" OR "insecticides" OR "bactericide" OR "bactericides" OR "fungicide" OR "fungicides" OR "herbicide" OR "herbicides" OR "acaricide" OR "acaricides" OR "nematicide" OR "nematicides" OR "pesticide" OR "pesticides" OR "antifungal" OR "antibacterial" OR "antimycotic" OR "antimycotics" OR "antibiotic" OR "antibiotics" OR "antimicrobial" OR "antifouling" OR "anti-fouling" OR "tetracycline" OR "atrazine" OR "oxytetracycline" OR "tributyltin" OR "ciprofloxacin" OR "glyphosate" OR "neonicotinoid" OR "triclosan" OR "vancomycin" OR "amphotericin" OR "methicillin" OR "chlorpyrifos" OR "deltamethrin" OR "fluconazole" OR "fluconazoles" OR "imidacloprid" OR "sulfonamide" OR "sulfonamides" OR "sulfamethoxazole" OR "sulfamethoxazoles" OR "quinolone" OR "quinolones" OR "fipronil" OR "mancozeb" OR "thiamethoxam" OR "trimethoprim" OR "benomyl")	AND	((("arbuscular" OR "vesicular arbuscular" OR "VA" OR "arbutoid" OR "ericoid" OR "ericaceous" OR "monotropoid" OR "orchid" OR "orchidaceous") NEAR/1 ("mycorrhiza" OR "mycorrhizas" OR "mycorrhizal" OR "mycorrhizae" OR "mycorrhization")) OR “ectomycorrhiza” OR “ectomycorrhizas” OR “ectomycorrhizal” OR “ectomycorrhizae” OR “ectomycorrhization” OR “endomycorrhiza” OR “endomycorrhizas” OR “endomycorrhizal” OR “endomycorrhizae” OR “endomycorrhization” OR “ectendomycorrhiza” OR “ectendomycorrhizas” OR “ectendomycorrhizal” OR “ectendomycorrhizae” OR “ectendomycorrhization” OR "AM fungi" OR "AM fungus" OR "AM fungal" OR "VAM fungi" OR "VAM fungus" OR "VAM fungal" OR "EcM fungi" OR "EcM fungus" OR "EcM fungal" OR "ErM fungi" OR "ErM fungus" OR "ErM fungal" OR (("mycorrhiza" OR "mycorrhizas" OR "mycorrhizal" OR "mycorrhizae" OR "mycorrhization" OR "mycorrhized") AND ("inoculated" OR "inoculation" OR "fungal" OR "fungi" OR "fungus")))	)	NOT SU=("Acoustics" OR "Allergy" OR "Anesthesiology" OR "Anthropology" OR "Archaeology" OR "Architecture" OR "Area Studies" OR "Art" OR "Arts & Humanities-Other Topics" OR "Asian Studies" OR "Audiology & Speech-Language Pathology" OR "Automation & Control Systems" OR "Biomedical Social Sciences" OR "Business & Economics" OR "Cardiovascular System & Cardiology" OR "Classics" OR "Communication" OR "Computer Science" OR "Construction & Building Technology" OR "Criminology & Penology" OR "Critical Care Medicine" OR "Crystallography" OR "Cultural Studies" OR "Dance" OR "Demography" OR "Dentistry, Oral Surgery & Medicine" OR "Dermatology" OR "Development Studies" OR "Education & Educational Research" OR "Electrochemistry" OR "Emergency Medicine" OR "Ethnic Studies" OR "Family Studies" OR "Film, Radio & Television" OR "Gastroenterology & Hepatology" OR "General & Internal Medicine" OR "Geochemistry & Geophysics" OR "Geriatrics & Gerontology" OR "Government & Law" OR "Health Care Sciences & Services" OR "Hematology" OR "History" OR "History & Philosophy of Science" OR "Imaging Science & Photographic Technology" OR "Information Science & Library Science" OR "Instruments & Instrumentation" OR "Integrative & Complementary Medicine" OR "International Relations" OR "Legal Medicine" OR "Linguistics" OR "Literature" OR "Mathematical & Computational Biology" OR "Mathematical Methods In Social Sciences" OR "Mathematics" OR "Mechanics" OR "Medical Ethics" OR "Medical Informatics" OR "Medical Laboratory Technology" OR "Metallurgy & Metallurgical Engineering" OR "Microscopy" OR "Music" OR "Nuclear Science & Technology" OR "Nursing" OR "Obstetrics & Gynecology" OR "Oncology" OR "Operations Research & Management Science" OR "Ophthalmology" OR "Optics" OR "Orthopedics" OR "Otorhinolaryngology" OR "Pathology" OR "Pediatrics" OR "Philosophy" OR "Physics" OR "Polymer Science" OR "Public Administration" OR "Rehabilitation" OR "Religion" OR "Research & Experimental Medicine" OR "Respiratory System" OR "Rheumatology" OR "Robotics" OR "Social Issues" OR "Social Sciences Other Topics" OR "Social Work" OR "Sociology" OR "Sport Sciences" OR "Substance Abuse" OR "Surgery" OR "Telecommunications" OR "Theater" OR "Thermodynamics" OR "Transplantation" OR "Transportation" OR "Urology & Nephrology" OR "Women's Studies" OR "Biophysics" OR "Chemistry" OR "Energy & Fuels" OR "Engineering" OR "Entomology" OR "Materials Science" OR "Nutrition & Dietetics" OR "Psychiatry" OR "Psychology" OR "Radiology, Nuclear Medicine & Medical Imaging" OR "Reproductive Biology" OR "Spectroscopy" OR "Tropical Medicine" OR "Virology")	NOT TI=("review" OR "meta-analysis" OR "metaanalysis" OR "meta analysis")	882

CO2	TS=(	("CO(2) " OR "CO-2" OR "CO2" OR "CO 2"OR "eCO(2)" OR "eCO2" OR “carbon dioxide” OR "carbon-dioxide") NEAR/10 ("experimental" OR "experiment" OR "experiments" OR "experimented" OR "experimenting" OR "atmospheric" OR "enrichment" OR "enriched" OR "enriching" OR "enhance" OR "enhanced" OR "enhancing" OR "elevate" OR "elevated" OR "elevating" OR "increase" OR "increased" OR "increasing" OR "high" OR "higher" OR "raised" OR "rising" OR "global" OR "climate" OR "climate change" OR "climatic change")	AND	((("arbuscular" OR "vesicular arbuscular" OR "VA" OR "arbutoid" OR "ericoid" OR "ericaceous" OR "monotropoid" OR "orchid" OR "orchidaceous") NEAR/1 ("mycorrhiza" OR "mycorrhizas" OR "mycorrhizal" OR "mycorrhizae" OR "mycorrhization")) OR “ectomycorrhiza” OR “ectomycorrhizas” OR “ectomycorrhizal” OR “ectomycorrhizae” OR “ectomycorrhization” OR "endomycorrhiza” OR "endomycorrhizas” OR "endomycorrhizal” OR “endomycorrhizae” OR “endomycorrhization” OR “ectendomycorrhiza” OR “ectendomycorrhizas” OR “ectendomycorrhizal” OR “ectendomycorrhizae” OR “ectendomycorrhization” OR "AM fungi" OR "AM fungus" OR "AM fungal" OR "VAM fungi" OR "VAM fungus" OR "VAM fungal" OR "EcM fungi" OR "EcM fungus" OR "EcM fungal" OR "ErM fungi" OR "ErM fungus" OR "ErM fungal" OR (("mycorrhiza" OR "mycorrhizas" OR "mycorrhizal" OR "mycorrhizae" OR "mycorrhization" OR "mycorrhized") AND ("inoculated" OR "inoculation" OR "fungal" OR "fungi" OR "fungus")))	)	NOT SU=("Acoustics" OR "Allergy" OR "Anesthesiology" OR "Anthropology" OR "Archaeology" OR "Architecture" OR "Area Studies" OR "Art" OR "Arts & Humanities-Other Topics" OR "Asian Studies" OR "Audiology & Speech-Language Pathology" OR "Automation & Control Systems" OR "Biomedical Social Sciences" OR "Business & Economics" OR "Cardiovascular System & Cardiology" OR "Classics" OR "Communication" OR "Computer Science" OR "Construction & Building Technology" OR "Criminology & Penology" OR "Critical Care Medicine" OR "Crystallography" OR "Cultural Studies" OR "Dance" OR "Demography" OR "Dentistry, Oral Surgery & Medicine" OR "Dermatology" OR "Development Studies" OR "Education & Educational Research" OR "Electrochemistry" OR "Emergency Medicine" OR "Ethnic Studies" OR "Family Studies" OR "Film, Radio & Television" OR "Gastroenterology & Hepatology" OR "General & Internal Medicine" OR "Geochemistry & Geophysics" OR "Geriatrics & Gerontology" OR "Government & Law" OR "Health Care Sciences & Services" OR "Hematology" OR "History" OR "History & Philosophy of Science" OR "Imaging Science & Photographic Technology" OR "Information Science & Library Science" OR "Instruments & Instrumentation" OR "Integrative & Complementary Medicine" OR "International Relations" OR "Legal Medicine" OR "Linguistics" OR "Literature" OR "Mathematical & Computational Biology" OR "Mathematical Methods In Social Sciences" OR "Mathematics" OR "Mechanics" OR "Medical Ethics" OR "Medical Informatics" OR "Medical Laboratory Technology" OR "Metallurgy & Metallurgical Engineering" OR "Microscopy" OR "Music" OR "Nuclear Science & Technology" OR "Nursing" OR "Obstetrics & Gynecology" OR "Oncology" OR "Operations Research & Management Science" OR "Ophthalmology" OR "Optics" OR "Orthopedics" OR "Otorhinolaryngology" OR "Pathology" OR "Pediatrics" OR "Philosophy" OR "Physics" OR "Polymer Science" OR "Public Administration" OR "Rehabilitation" OR "Religion" OR "Research & Experimental Medicine" OR "Respiratory System" OR "Rheumatology" OR "Robotics" OR "Social Issues" OR "Social Sciences Other Topics" OR "Social Work" OR "Sociology" OR "Sport Sciences" OR "Substance Abuse" OR "Surgery" OR "Telecommunications" OR "Theater" OR "Thermodynamics" OR "Transplantation" OR "Transportation" OR "Urology & Nephrology" OR "Women's Studies" OR "Anatomy & Morphology" OR "Energy & Fuels" OR "Engineering" OR "Immunology" OR "Infectious Diseases" OR "Materials Science" OR "Neurosciences & Neurology" OR "Physical Geography" OR "Psychiatry" OR "Psychology" OR "Radiology, Nuclear Medicine & Medical Imaging" OR "Remote Sensing" OR "Spectroscopy" OR "Urban Studies")	NOT TI=("review" OR "meta-analysis" OR "metaanalysis" OR "meta analysis")	751
Temperatur_ warming	TS=(	("warming") NOT ("cold" OR "cool" OR "cooling" OR "chill" OR "chills" OR "chilling")	AND	((("arbuscular" OR "vesicular arbuscular" OR "VA" OR "arbutoid" OR "ericoid" OR "ericaceous" OR "monotropoid" OR "orchid" OR "orchidaceous") NEAR/1 ("mycorrhiza" OR "mycorrhizas" OR "mycorrhizal" OR "mycorrhizae" OR "mycorrhization")) OR “ectomycorrhiza” OR “ectomycorrhizas” OR “ectomycorrhizal” OR “ectomycorrhizae” OR	)	NOT SU=("Acoustics" OR "Allergy" OR "Anesthesiology" OR "Anthropology" OR "Archaeology" OR "Architecture" OR "Area Studies" OR "Art" OR "Arts & Humanities-Other Topics" OR "Asian Studies" OR "Audiology & Speech-Language Pathology" OR "Automation & Control Systems" OR "Biomedical Social Sciences" OR "Business & Economics" OR "Cardiovascular System & Cardiology" OR "Classics" OR "Communication" OR "Computer Science" OR "Construction & Building Technology" OR "Criminology & Penology" OR "Critical Care Medicine" OR "Crystallography" OR "Cultural Studies" OR "Dance" OR "Demography" OR "Dentistry, Oral Surgery & Medicine" OR "Dermatology" OR "Development Studies" OR "Education & Educational Research" OR "Electrochemistry" OR "Emergency Medicine" OR "Ethnic Studies" OR "Family Studies" OR "Film, Radio & Television" OR "Gastroenterology & Hepatology" OR "General & Internal Medicine" OR "Geochemistry & Geophysics" OR	NOT TI=("review" OR "meta-analysis" OR "metaanalysis" OR "meta	228

				“ectomycorrhization” OR "endomycorrhiza” OR "endomycorrhizas” OR “endomycorrhizal” OR “endomycorrhizae” OR “endomycorrhization” OR “ectendomycorrhiza” OR “ectendomycorrhizas” OR “ectendomycorrhizal” OR “ectendomycorrhizae” OR “ectendomycorrhization” OR "AM fungi" OR "AM fungus" OR "AM fungal" OR "VAM fungi" OR "VAM fungus" OR "VAM fungal" OR "EcM fungi" OR "EcM fungus" OR "EcM fungal" OR "ErM fungi" OR "ErM fungus" OR "ErM fungal" OR (("mycorrhiza" OR "mycorrhizas" OR "mycorrhizal" OR "mycorrhizae" OR "mycorrhization" OR "mycorrhized") AND ("inoculated" OR "inoculation" OR "fungal" OR "fungi" OR "fungus")))		"Geriatrics & Gerontology" OR "Government & Law" OR "Health Care Sciences & Services" OR "Hematology" OR "History" OR "History & Philosophy of Science" OR "Imaging Science & Photographic Technology" OR "Information Science & Library Science" OR "Instruments & Instrumentation" OR "Integrative & Complementary Medicine" OR "International Relations" OR "Legal Medicine" OR "Linguistics" OR "Literature" OR "Mathematical & Computational Biology" OR "Mathematical Methods In Social Sciences" OR "Mathematics" OR "Mechanics" OR "Medical Ethics" OR "Medical Informatics" OR "Medical Laboratory Technology" OR "Metallurgy & Metallurgical Engineering" OR "Microscopy" OR "Music" OR "Nuclear Science & Technology" OR "Nursing" OR "Obstetrics & Gynecology" OR "Oncology" OR "Operations Research & Management Science" OR "Ophthalmology" OR "Optics" OR "Orthopedics" OR "Otorhinolaryngology" OR "Pathology" OR "Pediatrics" OR "Philosophy" OR "Physics" OR "Polymer Science" OR "Public Administration" OR "Rehabilitation" OR "Religion" OR "Research & Experimental Medicine" OR "Respiratory System" OR "Rheumatology" OR "Robotics" OR "Social Issues" OR "Social Sciences Other Topics" OR "Social Work" OR "Sociology" OR "Sport Sciences" OR "Substance Abuse" OR "Surgery" OR "Telecommunications" OR "Theater" OR "Thermodynamics" OR "Transplantation" OR "Transportation" OR "Urology & Nephrology" OR "Women's Studies" OR "Anatomy & Morphology" OR "Astronomy & Astrophysics" OR "Behavioral Sciences" OR "Biophysics" OR "Chemistry" OR "Developmental Biology" OR "Energy & Fuels" OR "Engineering" OR "Infectious Diseases" OR "Materials Science" OR "Mineralogy" OR "Mining & Mineral Processing" OR "Neurosciences & Neurology" OR "Paleontology" OR "Pharmacology & Pharmacy" OR "Psychiatry" OR "Psychology" OR "Public, Environmental & Occupational Health" OR "Radiology, Nuclear Medicine & Medical Imaging" OR "Remote Sensing" OR "Reproductive Biology" OR "Spectroscopy" OR "Virology")	analysis ")	
Temperature _temperature	TS=(	("temperature" OR "temperatures") NEAR/2 ("experimental" OR "experimentally" OR "enhance" OR "enhanced" OR "enhancing" OR "elevate" OR "elevated" OR "elevating" OR "increase" OR "increased" OR "increasing" OR "high" OR "higher" OR "raised" OR "rising")	AND	((("arbuscular" OR "vesicular arbuscular" OR "VA" OR "arbutoid" OR "ericoid" OR "ericaceous" OR "monotropoid" OR "orchid" OR "orchidaceous") NEAR/1 ("mycorrhiza" OR "mycorrhizas" OR "mycorrhizal" OR "mycorrhizae" OR "mycorrhization")) OR “ectomycorrhiza” OR “ectomycorrhizas” OR “ectomycorrhizal” OR “ectomycorrhizae” OR “ectomycorrhization” OR "endomycorrhiza” OR "endomycorrhizas” OR “endomycorrhizal” OR “endomycorrhizae” OR “endomycorrhization” OR “ectendomycorrhiza” OR “ectendomycorrhizas” OR “ectendomycorrhizal” OR	)	NOT SU=("Acoustics" OR "Allergy" OR "Anesthesiology" OR "Anthropology" OR "Archaeology" OR "Architecture" OR "Area Studies" OR "Art" OR "Arts & Humanities-Other Topics" OR "Asian Studies" OR "Audiology & Speech-Language Pathology" OR "Automation & Control Systems" OR "Biomedical Social Sciences" OR "Business & Economics" OR "Cardiovascular System & Cardiology" OR "Classics" OR "Communication" OR "Computer Science" OR "Construction & Building Technology" OR "Criminology & Penology" OR "Critical Care Medicine" OR "Crystallography" OR "Cultural Studies" OR "Dance" OR "Demography" OR "Dentistry, Oral Surgery & Medicine" OR "Dermatology" OR "Development Studies" OR "Education & Educational Research" OR "Electrochemistry" OR "Emergency Medicine" OR "Ethnic Studies" OR "Family Studies" OR "Film, Radio & Television" OR "Gastroenterology & Hepatology" OR "General & Internal Medicine" OR "Geochemistry & Geophysics" OR "Geriatrics & Gerontology" OR "Government & Law" OR "Health Care Sciences & Services" OR "Hematology" OR "History" OR "History & Philosophy of Science" OR "Imaging Science & Photographic Technology" OR "Information Science & Library Science" OR "Instruments & Instrumentation" OR "Integrative & Complementary Medicine" OR "International Relations" OR "Legal Medicine" OR "Linguistics" OR "Literature" OR "Mathematical & Computational Biology" OR "Mathematical Methods In Social Sciences" OR "Mathematics" OR "Mechanics" OR "Medical Ethics" OR "Medical Informatics" OR "Medical Laboratory Technology" OR "Metallurgy & Metallurgical Engineering" OR "Microscopy" OR "Music" OR "Nuclear Science &	NOT TI=("review" OR "meta-analysis" OR "metaanalysis" OR "meta analysis")	261

				“ectendomycorrhizae” OR “ectendomycorrhization” OR "AM fungi" OR "AM fungus" OR "AM fungal" OR "VAM fungi" OR "VAM fungus" OR "VAM fungal" OR "EcM fungi" OR "EcM fungus" OR "EcM fungal" OR "ErM fungi" OR "ErM fungus" OR "ErM fungal" OR (("mycorrhiza" OR "mycorrhizas" OR "mycorrhizal" OR "mycorrhizae" OR "mycorrhization" OR "mycorrhized") AND ("inoculated" OR "inoculation" OR "fungal" OR "fungi" OR "fungus")))		Technology" OR "Nursing" OR "Obstetrics & Gynecology" OR "Oncology" OR "Operations Research & Management Science" OR "Ophthalmology" OR "Optics" OR "Orthopedics" OR "Otorhinolaryngology" OR "Pathology" OR "Pediatrics" OR "Philosophy" OR "Physics" OR "Polymer Science" OR "Public Administration" OR "Rehabilitation" OR "Religion" OR "Research & Experimental Medicine" OR "Respiratory System" OR "Rheumatology" OR "Robotics" OR "Social Issues" OR "Social Sciences Other Topics" OR "Social Work" OR "Sociology" OR "Sport Sciences" OR "Substance Abuse" OR "Surgery" OR "Telecommunications" OR "Theater" OR "Thermodynamics" OR "Transplantation" OR "Transportation" OR "Urology & Nephrology" OR "Women's Studies" OR "Anatomy & Morphology" OR "Astronomy & Astrophysics" OR "Behavioral Sciences" OR "Biophysics" OR "Chemistry" OR "Developmental Biology" OR "Energy & Fuels" OR "Engineering" OR "Infectious Diseases" OR "Materials Science" OR "Mineralogy" OR "Mining & Mineral Processing" OR "Neurosciences & Neurology" OR "Paleontology" OR "Pharmacology & Pharmacy" OR "Psychiatry" OR "Psychology" OR "Public, Environmental & Occupational Health" OR "Radiology, Nuclear Medicine & Medical Imaging" OR "Remote Sensing" OR "Reproductive Biology" OR "Spectroscopy" OR "Virology")		
Temperature _heat	TS=(	("heat pulse" OR "heat pulses" OR "heat wave" OR "heat waves" OR "heat event" OR "heat events" OR "heat stress" OR "heat stresses")	AND	((("arbuscular" OR "vesicular arbuscular" OR "VA" OR "arbutoid" OR "ericoid" OR "ericaceous" OR "monotropoid" OR "orchid" OR "orchidaceous") NEAR/1 ("mycorrhiza" OR "mycorrhizas" OR "mycorrhizal" OR "mycorrhizae" OR "mycorrhization")) OR “ectomycorrhiza” OR “ectomycorrhizas” OR “ectomycorrhizal” OR “ectomycorrhizae” OR “ectomycorrhization” OR "endomycorrhiza” OR "endomycorrhizas” OR “endomycorrhizal” OR “endomycorrhizae” OR “endomycorrhization” OR “ectendomycorrhiza” OR “ectendomycorrhizas” OR “ectendomycorrhizal” OR “ectendomycorrhizae” OR “ectendomycorrhization” OR "AM fungi" OR "AM fungus" OR "AM fungal" OR "VAM fungi" OR "VAM fungus" OR "VAM fungal" OR "EcM fungi" OR "EcM fungus" OR "EcM fungal" OR "ErM fungi" OR "ErM fungus" OR "ErM fungal" OR (("mycorrhiza" OR "mycorrhizas" OR	)	NOT SU=("Acoustics" OR "Allergy" OR "Anesthesiology" OR "Anthropology" OR "Archaeology" OR "Architecture" OR "Area Studies" OR "Art" OR "Arts & Humanities-Other Topics" OR "Asian Studies" OR "Audiology & Speech-Language Pathology" OR "Automation & Control Systems" OR "Biomedical Social Sciences" OR "Business & Economics" OR "Cardiovascular System & Cardiology" OR "Classics" OR "Communication" OR "Computer Science" OR "Construction & Building Technology" OR "Criminology & Penology" OR "Critical Care Medicine" OR "Crystallography" OR "Cultural Studies" OR "Dance" OR "Demography" OR "Dentistry, Oral Surgery & Medicine" OR "Dermatology" OR "Development Studies" OR "Education & Educational Research" OR "Electrochemistry" OR "Emergency Medicine" OR "Ethnic Studies" OR "Family Studies" OR "Film, Radio & Television" OR "Gastroenterology & Hepatology" OR "General & Internal Medicine" OR "Geochemistry & Geophysics" OR "Geriatrics & Gerontology" OR "Government & Law" OR "Health Care Sciences & Services" OR "Hematology" OR "History" OR "History & Philosophy of Science" OR "Imaging Science & Photographic Technology" OR "Information Science & Library Science" OR "Instruments & Instrumentation" OR "Integrative & Complementary Medicine" OR "International Relations" OR "Legal Medicine" OR "Linguistics" OR "Literature" OR "Mathematical & Computational Biology" OR "Mathematical Methods In Social Sciences" OR "Mathematics" OR "Mechanics" OR "Medical Ethics" OR "Medical Informatics" OR "Medical Laboratory Technology" OR "Metallurgy & Metallurgical Engineering" OR "Microscopy" OR "Music" OR "Nuclear Science & Technology" OR "Nursing" OR "Obstetrics & Gynecology" OR "Oncology" OR "Operations Research & Management Science" OR "Ophthalmology" OR "Optics" OR "Orthopedics" OR "Otorhinolaryngology" OR "Pathology" OR "Pediatrics" OR "Philosophy" OR "Physics" OR "Polymer Science" OR "Public Administration" OR "Rehabilitation" OR "Religion" OR "Research & Experimental Medicine" OR "Respiratory System" OR "Rheumatology" OR "Robotics" OR "Social Issues" OR "Social Sciences Other Topics" OR "Social Work" OR "Sociology" OR "Sport Sciences" OR "Substance Abuse" OR "Surgery" OR "Telecommunications" OR "Theater" OR "Thermodynamics" OR "Transplantation" OR "Transportation" OR "Urology &	NOT TI=("review" OR "meta-analysis" OR "metaanalysis" OR "meta analysis")	22

				"mycorrhizal" OR "mycorrhizae" OR "mycorrhization" OR "mycorrhized") AND ("inoculated" OR "inoculation" OR "fungal" OR "fungi" OR "fungus")))		Nephrology" OR "Women's Studies" OR "Anatomy & Morphology" OR "Astronomy & Astrophysics" OR "Behavioral Sciences" OR "Biophysics" OR "Chemistry" OR "Developmental Biology" OR "Energy & Fuels" OR "Engineering" OR "Infectious Diseases" OR "Materials Science" OR "Mineralogy" OR "Mining & Mineral Processing" OR "Neurosciences & Neurology" OR "Paleontology" OR "Pharmacology & Pharmacy" OR "Psychiatry" OR "Psychology" OR "Public, Environmental & Occupational Health" OR "Radiology, Nuclear Medicine & Medical Imaging" OR "Remote Sensing" OR "Reproductive Biology" OR "Spectroscopy" OR "Virology")		
Drought	TS=(	("drought stress" OR "drought stresses" OR "drought stressed" OR "water stress" OR "water stresses" OR "water stressed" OR "water deficit" OR "water deficient" OR "water deficiency" OR (("water" OR "watering" OR "drought" OR "droughts" OR "irrigation" OR "irrigations" OR "precipitation" OR "rainfall" OR "rainfalls" OR "soil moisture" OR "soil moistures" OR "soil water content" OR "soil water contents") NEAR/2 ("regime" OR "regimes" OR "treated" OR "treatment" OR "treatments" OR "addition" OR "supply" OR "level" OR "levels" OR "experimental" OR "supplemental"))) NOT (("irrigated with" AND ("saline water" OR "waste water")) OR (("transect" OR "transects" OR "gradient" OR "gradients") NEAR/5 ("rainfall" OR "rainfalls" OR "precipitation" OR "precipitations" OR "topographic" OR "latitudinal" OR "latitude" OR "longitudinal" OR "longitude" OR "elevational" OR "elevation" OR "altitudinal" OR "altitude")))	AND	((("arbuscular" OR "vesicular arbuscular" OR "VA" OR "arbutoid" OR "ericoid" OR "ericaceous" OR "monotropoid" OR "orchid" OR "orchidaceous") NEAR/1 ("mycorrhiza" OR "mycorrhizas" OR "mycorrhizal" OR "mycorrhizae" OR "mycorrhization")) OR “ectomycorrhiza” OR “ectomycorrhizas” OR “ectomycorrhizal” OR “ectomycorrhizae” OR “ectomycorrhization” OR "endomycorrhiza” OR endomycorrhizas” OR “endomycorrhizal” OR “endomycorrhizae” OR “endomycorrhization” OR “ectendomycorrhiza” OR “ectendomycorrhizas” OR “ectendomycorrhizal” OR “ectendomycorrhizae” OR “ectendomycorrhization” OR "AM fungi" OR "AM fungus" OR "AM fungal" OR "VAM fungi" OR "VAM fungus" OR "VAM fungal" OR "EcM fungi" OR EcM fungus" OR "EcM fungal" OR "ErM fungi" OR "ErM fungus" OR "ErM fungal" OR (("mycorrhiza" OR "mycorrhizas" OR "mycorrhizal" OR "mycorrhizae" OR "mycorrhization" OR "mycorrhized") AND ("inoculated" OR "inoculation" OR "fungal" OR "fungi" OR "fungus")))	)	NOT SU=("Acoustics" OR "Allergy" OR "Anesthesiology" OR "Anthropology" OR "Archaeology" OR "Architecture" OR "Area Studies" OR "Art" OR "Arts & Humanities-Other Topics" OR "Asian Studies" OR "Audiology & Speech-Language Pathology" OR "Automation & Control Systems" OR "Biomedical Social Sciences" OR "Business & Economics" OR "Cardiovascular System & Cardiology" OR "Classics" OR "Communication" OR "Computer Science" OR "Construction & Building Technology" OR "Criminology & Penology" OR "Critical Care Medicine" OR "Crystallography" OR "Cultural Studies" OR "Dance" OR "Demography" OR "Dentistry, Oral Surgery & Medicine" OR "Dermatology" OR "Development Studies" OR "Education & Educational Research" OR "Electrochemistry" OR "Emergency Medicine" OR "Ethnic Studies" OR "Family Studies" OR "Film, Radio & Television" OR "Gastroenterology & Hepatology" OR "General & Internal Medicine" OR "Geochemistry & Geophysics" OR "Geriatrics & Gerontology" OR "Government & Law" OR "Health Care Sciences & Services" OR "Hematology" OR "History" OR "History & Philosophy of Science" OR "Imaging Science & Photographic Technology" OR "Information Science & Library Science" OR "Instruments & Instrumentation" OR "Integrative & Complementary Medicine" OR "International Relations" OR "Legal Medicine" OR "Linguistics" OR "Literature" OR "Mathematical & Computational Biology" OR "Mathematical Methods In Social Sciences" OR "Mathematics" OR "Mechanics" OR "Medical Ethics" OR "Medical Informatics" OR "Medical Laboratory Technology" OR "Metallurgy & Metallurgical Engineering" OR "Microscopy" OR "Music" OR "Nuclear Science & Technology" OR "Nursing" OR "Obstetrics & Gynecology" OR "Oncology" OR "Operations Research & Management Science" OR "Ophthalmology" OR "Optics" OR "Orthopedics" OR "Otorhinolaryngology" OR "Pathology" OR "Pediatrics" OR "Philosophy" OR "Physics" OR "Polymer Science" OR "Public Administration" OR "Rehabilitation" OR "Religion" OR "Research & Experimental Medicine" OR "Respiratory System" OR "Rheumatology" OR "Robotics" OR "Social Issues" OR "Social Sciences Other Topics" OR "Social Work" OR "Sociology" OR "Sport Sciences" OR "Substance Abuse" OR "Surgery" OR "Telecommunications" OR "Theater" OR "Thermodynamics" OR "Transplantation" OR "Transportation" OR "Urology & Nephrology" OR "Women's Studies" OR "Anatomy & Morphology" OR "Astronomy & Astrophysics" OR "Geography" OR "Infectious Diseases" OR "Mineralogy" OR "Mining & Mineral Processing" OR "Neurosciences & Neurology" OR "Paleontology" OR "Parasitology" OR "Physical Geography" OR "Physiology" OR "Psychiatry" OR "Psychology" OR "Public, Environmental & Occupational Health" OR "Remote Sensing" OR "Virology")	NOT TI=("review" OR "meta-analysis" OR "metaanalysis" OR "meta analysis")	1330
ALAN	TS=(	((("light" OR "lights" OR "lighting" OR "brightness" OR	AND	((("arbuscular" OR "vesicular arbuscular" OR "VA" OR "arbutoid"	)	NOT SU=("Acoustics" OR "Allergy" OR "Anesthesiology" OR "Anthropology" OR "Archaeology" OR "Architecture" OR "Area Studies" OR "Art" OR "Arts & Humanities-	NOT TI=("re	3

		"skyglow" OR "sky glow" OR "illumination" OR "illuminance" OR "LED") NEAR/2 ("night" OR "night time" OR "nighttime" OR "nocturnal")) OR "light pollution" OR "light pollutions")		OR "ericoid" OR "ericaceous" OR "monotropoid" OR "orchid" OR "orchidaceous") NEAR/1 ("mycorrhiza" OR "mycorrhizas" OR "mycorrhizal" OR "mycorrhizae" OR "mycorrhization")) OR “ectomycorrhiza” OR “ectomycorrhizas” OR “ectomycorrhizal” OR “ectomycorrhizae” OR “ectomycorrhization” OR "endomycorrhiza” OR endomycorrhizas” OR “endomycorrhizal” OR “endomycorrhizae” OR “endomycorrhization” OR “ectendomycorrhiza” OR “ectendomycorrhizas” OR “ectendomycorrhizal” OR “ectendomycorrhizae” OR “ectendomycorrhization” OR "AM fungi" OR "AM fungus" OR "AM fungal" OR "VAM fungi" OR "VAM fungus" OR "VAM fungal" OR "EcM fungi" OR EcM fungus" OR "EcM fungal" OR "ErM fungi" OR "ErM fungus" OR "ErM fungal" OR (("mycorrhiza" OR "mycorrhizas" OR "mycorrhizal" OR "mycorrhizae" OR "mycorrhization" OR "mycorrhized") AND ("inoculated" OR "inoculation" OR "fungal" OR "fungi" OR "fungus")))		Other Topics" OR "Asian Studies" OR "Audiology & Speech-Language Pathology" OR "Automation & Control Systems" OR "Biomedical Social Sciences" OR "Business & Economics" OR "Cardiovascular System & Cardiology" OR "Classics" OR "Communication" OR "Computer Science" OR "Construction & Building Technology" OR "Criminology & Penology" OR "Critical Care Medicine" OR "Crystallography" OR "Cultural Studies" OR "Dance" OR "Demography" OR "Dentistry, Oral Surgery & Medicine" OR "Dermatology" OR "Development Studies" OR "Education & Educational Research" OR "Electrochemistry" OR "Emergency Medicine" OR "Ethnic Studies" OR "Family Studies" OR "Film, Radio & Television" OR "Gastroenterology & Hepatology" OR "General & Internal Medicine" OR "Geochemistry & Geophysics" OR "Geriatrics & Gerontology" OR "Government & Law" OR "Health Care Sciences & Services" OR "Hematology" OR "History" OR "History & Philosophy of Science" OR "Imaging Science & Photographic Technology" OR "Information Science & Library Science" OR "Instruments & Instrumentation" OR "Integrative & Complementary Medicine" OR "International Relations" OR "Legal Medicine" OR "Linguistics" OR "Literature" OR "Mathematical & Computational Biology" OR "Mathematical Methods In Social Sciences" OR "Mathematics" OR "Mechanics" OR "Medical Ethics" OR "Medical Informatics" OR "Medical Laboratory Technology" OR "Metallurgy & Metallurgical Engineering" OR "Microscopy" OR "Music" OR "Nuclear Science & Technology" OR "Nursing" OR "Obstetrics & Gynecology" OR "Oncology" OR "Operations Research & Management Science" OR "Ophthalmology" OR "Optics" OR "Orthopedics" OR "Otorhinolaryngology" OR "Pathology" OR "Pediatrics" OR "Philosophy" OR "Physics" OR "Polymer Science" OR "Public Administration" OR "Rehabilitation" OR "Religion" OR "Research & Experimental Medicine" OR "Respiratory System" OR "Rheumatology" OR "Robotics" OR "Social Issues" OR "Social Sciences Other Topics" OR "Social Work" OR "Sociology" OR "Sport Sciences" OR "Substance Abuse" OR "Surgery" OR "Telecommunications" OR "Theater" OR "Thermodynamics" OR "Transplantation" OR "Transportation" OR "Urology & Nephrology" OR "Women's Studies" OR "Astronomy & Astrophysics" OR "Chemistry" OR "Energy & Fuels" OR "Engineering" OR "Pharmacology & Pharmacy" OR "Public, Environmental & Occupational Health" OR "Remote Sensing")	view" OR "meta-analysis" OR "metaanalysis" OR "meta analysis")	
N&P deposition	TS=(	(("nitrogen" OR "active nitrogen" OR "reactive nitrogen" OR "ammonia" OR "ammonium" OR "nitrate" OR "NH4+" OR "NO3-" OR "phosphorus" OR "phosphate" OR "nutrient" OR "nutrients") AND ("pollution" OR "pollutant" OR "pollutants" OR "loading" OR "loadings" OR "input" OR "inputs" OR "enrichment" OR "enrichments" OR "atmospheric" OR "anthropogenic" OR "global change" OR "climate-change" OR	AND	((("arbuscular" OR "vesicular arbuscular" OR "VA" OR "arbutoid" OR "ericoid" OR "ericaceous" OR "monotropoid" OR "orchid" OR "orchidaceous") NEAR/1 ("mycorrhiza" OR "mycorrhizas" OR "mycorrhizal" OR "mycorrhizae" OR "mycorrhization")) OR “ectomycorrhiza” OR “ectomycorrhizas” OR “ectomycorrhizal” OR “ectomycorrhizae” OR “ectomycorrhization” OR "endomycorrhiza” OR	)	NOT SU=("Acoustics" OR "Allergy" OR "Anesthesiology" OR "Anthropology" OR "Archaeology" OR "Architecture" OR "Area Studies" OR "Art" OR "Arts & Humanities-Other Topics" OR "Asian Studies" OR "Audiology & Speech-Language Pathology" OR "Automation & Control Systems" OR "Biomedical Social Sciences" OR "Business & Economics" OR "Cardiovascular System & Cardiology" OR "Classics" OR "Communication" OR "Computer Science" OR "Construction & Building Technology" OR "Criminology & Penology" OR "Critical Care Medicine" OR "Crystallography" OR "Cultural Studies" OR "Dance" OR "Demography" OR "Dentistry, Oral Surgery & Medicine" OR "Dermatology" OR "Development Studies" OR "Education & Educational Research" OR "Electrochemistry" OR "Emergency Medicine" OR "Ethnic Studies" OR "Family Studies" OR "Film, Radio & Television" OR "Gastroenterology & Hepatology" OR "General & Internal Medicine" OR "Geochemistry & Geophysics" OR "Geriatrics & Gerontology" OR "Government & Law" OR "Health Care Sciences & Services" OR "Hematology" OR "History" OR "History & Philosophy of Science" OR	NOT TI=("review" OR "meta-analysis" OR "metaanalysis" OR "meta analysis")	517

		"climate change" OR "experiment" OR "experiments" OR "experimental" OR "experimentally" OR "simulated" OR "treatment" OR "treatments" OR "long-term" OR "short-term" OR "long term" OR "short term")) AND ("eutrophic" OR "eutrophication" OR "deposition")		endomycorrhizas” OR “endomycorrhizal” OR “endomycorrhizae” OR “endomycorrhization” OR “ectendomycorrhiza” OR “ectendomycorrhizas” OR “ectendomycorrhizal” OR “ectendomycorrhizae” OR “ectendomycorrhization” OR "AM fungi" OR "AM fungus" OR "AM fungal" OR "VAM fungi" OR "VAM fungus" OR "VAM fungal" OR "EcM fungi" OR EcM fungus" OR "EcM fungal" OR "ErM fungi" OR "ErM fungus" OR "ErM fungal" OR (("mycorrhiza" OR "mycorrhizas" OR "mycorrhizal" OR "mycorrhizae" OR "mycorrhization" OR "mycorrhized") AND ("inoculated" OR "inoculation" OR "fungal" OR "fungi" OR "fungus")))		"Imaging Science & Photographic Technology" OR "Information Science & Library Science" OR "Instruments & Instrumentation" OR "Integrative & Complementary Medicine" OR "International Relations" OR "Legal Medicine" OR "Linguistics" OR "Literature" OR "Mathematical & Computational Biology" OR "Mathematical Methods In Social Sciences" OR "Mathematics" OR "Mechanics" OR "Medical Ethics" OR "Medical Informatics" OR "Medical Laboratory Technology" OR "Metallurgy & Metallurgical Engineering" OR "Microscopy" OR "Music" OR "Nuclear Science & Technology" OR "Nursing" OR "Obstetrics & Gynecology" OR "Oncology" OR "Operations Research & Management Science" OR "Ophthalmology" OR "Optics" OR "Orthopedics" OR "Otorhinolaryngology" OR "Pathology" OR "Pediatrics" OR "Philosophy" OR "Physics" OR "Polymer Science" OR "Public Administration" OR "Rehabilitation" OR "Religion" OR "Research & Experimental Medicine" OR "Respiratory System" OR "Rheumatology" OR "Robotics" OR "Social Issues" OR "Social Sciences Other Topics" OR "Social Work" OR "Sociology" OR "Sport Sciences" OR "Substance Abuse" OR "Surgery" OR "Telecommunications" OR "Theater" OR "Thermodynamics" OR "Transplantation" OR "Transportation" OR "Urology & Nephrology" OR "Women's Studies" OR "Biochemistry & Molecular Biology" OR "Cell Biology" OR "Energy & Fuels" OR "Materials Science" OR "Pharmacology & Pharmacy" OR "Public, Environmental & Occupational Health" OR "Veterinary Sciences")		
Heavy metals	TS=(	("heavy metal" OR "heavy-metal" OR "heavy metals" OR "heavy-metals" OR "Zn" OR "zinc" OR " Cu" OR "copper" OR "iron" OR "Fe" OR "manganese" OR "Mn" OR "Cd" OR "cadmium" OR "Pb" OR "lead" OR "Cr" OR "chromium" OR "chromium(vi)" OR "Cr(vi)" OR "hexavalent chromium" OR "hexavalent Cr" OR "Hg" OR "mercury" OR "arsenic") AND ("experiment" OR "experiments" OR "experimental" OR "experimentally" OR "simulated" OR "treatment" OR "treatments" OR "addition" OR "additions" OR "level" OR "levels" OR "exposure" OR "exposures" OR "exposed" OR "stress" OR "stresses" OR "stressed" OR "treatment" OR "treatments" OR "treated" OR "experiment" OR "experiments" OR "experimental" OR	AND	((("arbuscular" OR "vesicular arbuscular" OR "VA" OR "arbutoid" OR "ericoid" OR "ericaceous" OR "monotropoid" OR "orchid" OR "orchidaceous") NEAR/1 ("mycorrhiza" OR "mycorrhizas" OR "mycorrhizal" OR "mycorrhizae" OR "mycorrhization")) OR “ectomycorrhiza” OR “ectomycorrhizas” OR “ectomycorrhizal” OR “ectomycorrhizae” OR “ectomycorrhization” OR "endomycorrhiza” OR endomycorrhizas” OR “endomycorrhizal” OR “endomycorrhizae” OR “endomycorrhization” OR “ectendomycorrhiza” OR “ectendomycorrhizas” OR “ectendomycorrhizal” OR “ectendomycorrhizae” OR “ectendomycorrhization” OR "AM fungi" OR "AM fungus" OR "AM fungal" OR "VAM fungi" OR "VAM fungus" OR "VAM fungal" OR "EcM	)	NOT SU=("Acoustics" OR "Allergy" OR "Anesthesiology" OR "Anthropology" OR "Archaeology" OR "Architecture" OR "Area Studies" OR "Art" OR "Arts & Humanities-Other Topics" OR "Asian Studies" OR "Audiology & Speech-Language Pathology" OR "Automation & Control Systems" OR "Biomedical Social Sciences" OR "Business & Economics" OR "Cardiovascular System & Cardiology" OR "Classics" OR "Communication" OR "Computer Science" OR "Construction & Building Technology" OR "Criminology & Penology" OR "Critical Care Medicine" OR "Crystallography" OR "Cultural Studies" OR "Dance" OR "Demography" OR "Dentistry, Oral Surgery & Medicine" OR "Dermatology" OR "Development Studies" OR "Education & Educational Research" OR "Electrochemistry" OR "Emergency Medicine" OR "Ethnic Studies" OR "Family Studies" OR "Film, Radio & Television" OR "Gastroenterology & Hepatology" OR "General & Internal Medicine" OR "Geochemistry & Geophysics" OR "Geriatrics & Gerontology" OR "Government & Law" OR "Health Care Sciences & Services" OR "Hematology" OR "History" OR "History & Philosophy of Science" OR "Imaging Science & Photographic Technology" OR "Information Science & Library Science" OR "Instruments & Instrumentation" OR "Integrative & Complementary Medicine" OR "International Relations" OR "Legal Medicine" OR "Linguistics" OR "Literature" OR "Mathematical & Computational Biology" OR "Mathematical Methods In Social Sciences" OR "Mathematics" OR "Mechanics" OR "Medical Ethics" OR "Medical Informatics" OR "Medical Laboratory Technology" OR "Metallurgy & Metallurgical Engineering" OR "Microscopy" OR "Music" OR "Nuclear Science & Technology" OR "Nursing" OR "Obstetrics & Gynecology" OR "Oncology" OR "Operations Research & Management Science" OR "Ophthalmology" OR "Optics" OR "Orthopedics" OR "Otorhinolaryngology" OR "Pathology" OR "Pediatrics" OR "Philosophy" OR "Physics" OR "Polymer Science" OR "Public Administration" OR "Rehabilitation" OR "Religion" OR "Research & Experimental Medicine" OR	NOT TI=("review" OR "meta-analysis" OR "metaanalysis" OR "meta analysis")	1372

		"experimenting" OR "experimented") AND ("toxicant " OR "toxicants" OR "toxicity" OR "toxic" OR "pollutant" OR "pollutants" OR "pollution" OR "polluted" OR "contaminant" OR "contaminants" OR "contamination" OR "contaminated" OR "toxin" OR "toxins" OR "noxious" OR "phytoremediation" OR "bioremediation" OR "remediation" OR "tolerance" OR "sensitivity")		fungi" OR EcM fungus" OR "EcM fungal" OR "ErM fungi" OR "ErM fungus" OR "ErM fungal" OR (("mycorrhiza" OR "mycorrhizas" OR "mycorrhizal" OR "mycorrhizae" OR "mycorrhization" OR "mycorrhized") AND ("inoculated" OR "inoculation" OR "fungal" OR "fungi" OR "fungus")))		"Respiratory System" OR "Rheumatology" OR "Robotics" OR "Social Issues" OR "Social Sciences Other Topics" OR "Social Work" OR "Sociology" OR "Sport Sciences" OR "Substance Abuse" OR "Surgery" OR "Telecommunications" OR "Theater" OR "Thermodynamics" OR "Transplantation" OR "Transportation" OR "Urology & Nephrology" OR "Women's Studies" OR "Astronomy & Astrophysics" OR "Chemistry" OR "Energy & Fuels" OR "Geography" OR "Geology" OR "Infectious Diseases" OR "Materials Science" OR "Mineralogy" OR "Mining & Mineral Processing" OR "Neurosciences & Neurology" OR "Nutrition & Dietetics" OR "Paleontology" OR "Pharmacology & Pharmacy" OR "Physical Geography" OR "Psychiatry" OR "Psychology" OR "Radiology, Nuclear Medicine & Medical Imaging" OR "Remote Sensing" OR "Spectroscopy" OR "Tropical Medicine" OR "Urban Studies" OR "Virology")		
sodicity	TS=(	(("salinity" OR "salinities" OR "saline" OR "sodic" OR "sodicity" OR "sodication" OR "salinized" OR "salinization" OR "salt" OR "salts" OR "NaCl" OR "sodium" OR "ESP" OR "exchangeable sodium percentages" OR "exchangeable sodium percentage" OR "SAR" OR "sodium adsorption ratio" OR "sodium adsorption ratios") NEAR/10 ("experiment" OR "experiments" OR "experimental" OR "experimentally" OR "simulated" OR "treatment" OR "treatments" OR "addition" OR "additions" OR "exposure" OR "exposures" OR "exposed" OR "stress" OR "stresses" OR "stressed" OR "treatment" OR "treatments" OR "treated" OR "experiment" OR "experiments" OR "experimental" OR "experimenting" OR "experimented"))	AND	((("arbuscular" OR "vesicular arbuscular" OR "VA" OR "arbutoid" OR "ericoid" OR "ericaceous" OR "monotropoid" OR "orchid" OR "orchidaceous") NEAR/1 ("mycorrhiza" OR "mycorrhizas" OR "mycorrhizal" OR "mycorrhizae" OR "mycorrhization")) OR “ectomycorrhiza” OR “ectomycorrhizas” OR “ectomycorrhizal” OR “ectomycorrhizae” OR “ectomycorrhization” OR “endomycorrhiza” OR endomycorrhizas” OR “endomycorrhizal” OR “endomycorrhizae” OR “endomycorrhization” OR “ectendomycorrhiza” OR “ectendomycorrhizas” OR “ectendomycorrhizal” OR “ectendomycorrhizae” OR “ectendomycorrhization” OR "AM fungi" OR "AM fungus" OR "AM fungal" OR "VAM fungi" OR "VAM fungus" OR "VAM fungal" OR "EcM fungi" OR EcM fungus" OR "EcM fungal" OR "ErM fungi" OR "ErM fungus" OR "ErM fungal" OR (("mycorrhiza" OR "mycorrhizas" OR "mycorrhizal" OR "mycorrhizae" OR "mycorrhization" OR "mycorrhized") AND ("inoculated" OR "inoculation"	)	NOT SU=("Acoustics" OR "Allergy" OR "Anesthesiology" OR "Anthropology" OR "Archaeology" OR "Architecture" OR "Area Studies" OR "Art" OR "Arts & Humanities-Other Topics" OR "Asian Studies" OR "Audiology & Speech-Language Pathology" OR "Automation & Control Systems" OR "Biomedical Social Sciences" OR "Business & Economics" OR "Cardiovascular System & Cardiology" OR "Classics" OR "Communication" OR "Computer Science" OR "Construction & Building Technology" OR "Criminology & Penology" OR "Critical Care Medicine" OR "Crystallography" OR "Cultural Studies" OR "Dance" OR "Demography" OR "Dentistry, Oral Surgery & Medicine" OR "Dermatology" OR "Development Studies" OR "Education & Educational Research" OR "Electrochemistry" OR "Emergency Medicine" OR "Ethnic Studies" OR "Family Studies" OR "Film, Radio & Television" OR "Gastroenterology & Hepatology" OR "General & Internal Medicine" OR "Geochemistry & Geophysics" OR "Geriatrics & Gerontology" OR "Government & Law" OR "Health Care Sciences & Services" OR "Hematology" OR "History" OR "History & Philosophy of Science" OR "Imaging Science & Photographic Technology" OR "Information Science & Library Science" OR "Instruments & Instrumentation" OR "Integrative & Complementary Medicine" OR "International Relations" OR "Legal Medicine" OR "Linguistics" OR "Literature" OR "Mathematical & Computational Biology" OR "Mathematical Methods In Social Sciences" OR "Mathematics" OR "Mechanics" OR "Medical Ethics" OR "Medical Informatics" OR "Medical Laboratory Technology" OR "Metallurgy & Metallurgical Engineering" OR "Microscopy" OR "Music" OR "Nuclear Science & Technology" OR "Nursing" OR "Obstetrics & Gynecology" OR "Oncology" OR "Operations Research & Management Science" OR "Ophthalmology" OR "Optics" OR "Orthopedics" OR "Otorhinolaryngology" OR "Pathology" OR "Pediatrics" OR "Philosophy" OR "Physics" OR "Polymer Science" OR "Public Administration" OR "Rehabilitation" OR "Religion" OR "Research & Experimental Medicine" OR "Respiratory System" OR "Rheumatology" OR "Robotics" OR "Social Issues" OR "Social Sciences Other Topics" OR "Social Work" OR "Sociology" OR "Sport Sciences" OR "Substance Abuse" OR "Surgery" OR "Telecommunications" OR "Theater" OR "Thermodynamics" OR "Transplantation" OR "Transportation" OR "Urology & Nephrology" OR "Women's Studies" OR "Infectious Diseases" OR "Mineralogy" OR "Mining & Mineral Processing" OR "Paleontology" OR "Psychiatry" OR "Psychology" OR "Radiology, Nuclear Medicine & Medical Imaging" OR "Remote Sensing")	NOT TI=("review" OR "meta-analysis" OR "metaanalysis" OR "meta analysis")	730

				OR "fungal" OR "fungi" OR "fungus")))				
Species invasion	TS=(	((("exotic" OR "alien" OR "invasional" OR "invasion" OR "invasions" OR "invasive" OR "invasives" OR "invading" OR "non-native" OR "nonnative" OR "non native" OR "introduced" OR “non-indigenous” OR “nonindigenous” OR “non indigenous” OR "invasiveness" OR "invasibility") NEAR/10 ("aliens" OR "alien" OR "archaeophytes" OR "archaeophyte" OR "bacteria" OR "bacterias" OR "bacterial" OR "exotics" OR "flora" OR "fungi" OR "fungal" OR "grasses" OR "grass" OR "invaders" OR "invader" OR "invertebrates" OR "invertebrate" OR "isolates" OR "isolate" OR "macroalgae" OR "macroalga" OR "neophytes" OR "neophyte" OR "pests" OR "pest" OR "plants" OR "plant" OR "shrub" OR "shrubs" OR "species" OR "strains" OR "strain" OR "trees" OR "tree" OR "weeds" OR "weed" OR "biota" OR "naturalised" OR "naturalized" OR "inference" OR "inferences" OR "biological" OR "ecological" OR "meltdown")) NOT (("paired" NEAR/4 "sites") OR "monitoring" OR ((" hyphal" OR "cell") NEAR/4 "invasion") OR "fu ngal infection" OR (("transect" OR "transects" OR "gradient" OR "gradients") NEAR/1 ("latitudinal " OR "latitude" OR "longitudinal" OR "longitude" OR "elevational" OR "elevation" OR "altitudinal" OR "altitude"))))	AND	((("arbuscular" OR "vesicular arbuscular" OR "VA" OR "arbutoid" OR "ericoid" OR "ericaceous" OR "monotropoid" OR "orchid" OR "orchidaceous") NEAR/1 ("mycorrhiza" OR "mycorrhizas" OR "mycorrhizal" OR "mycorrhizae" OR "mycorrhization")) OR “ectomycorrhiza” OR “ectomycorrhizas” OR “ectomycorrhizal” OR “ectomycorrhizae” OR “ectomycorrhization” OR "endomycorrhiza” OR endomycorrhizas” OR “endomycorrhizal” OR “endomycorrhizae” OR “endomycorrhization” OR “ectendomycorrhiza” OR “ectendomycorrhizas” OR “ectendomycorrhizal” OR “ectendomycorrhizae” OR “ectendomycorrhization” OR "AM fungi" OR "AM fungus" OR "AM fungal" OR "VAM fungi" OR "VAM fungus" OR "VAM fungal" OR "EcM fungi" OR EcM fungus" OR "EcM fungal" OR "ErM fungi" OR "ErM fungus" OR "ErM fungal" OR (("mycorrhiza" OR "mycorrhizas" OR "mycorrhizal" OR "mycorrhizae" OR "mycorrhization" OR "mycorrhized") AND ("inoculated" OR "inoculation" OR "fungal" OR "fungi" OR "fungus")))	)	NOT SU=("Acoustics" OR "Allergy" OR "Anesthesiology" OR "Anthropology" OR "Archaeology" OR "Architecture" OR "Area Studies" OR "Art" OR "Arts & Humanities-Other Topics" OR "Asian Studies" OR "Audiology & Speech-Language Pathology" OR "Automation & Control Systems" OR "Biomedical Social Sciences" OR "Business & Economics" OR "Cardiovascular System & Cardiology" OR "Classics" OR "Communication" OR "Computer Science" OR "Construction & Building Technology" OR "Criminology & Penology" OR "Critical Care Medicine" OR "Crystallography" OR "Cultural Studies" OR "Dance" OR "Demography" OR "Dentistry, Oral Surgery & Medicine" OR "Dermatology" OR "Development Studies" OR "Education & Educational Research" OR "Electrochemistry" OR "Emergency Medicine" OR "Ethnic Studies" OR "Family Studies" OR "Film, Radio & Television" OR "Gastroenterology & Hepatology" OR "General & Internal Medicine" OR "Geochemistry & Geophysics" OR "Geriatrics & Gerontology" OR "Government & Law" OR "Health Care Sciences & Services" OR "Hematology" OR "History" OR "History & Philosophy of Science" OR "Imaging Science & Photographic Technology" OR "Information Science & Library Science" OR "Instruments & Instrumentation" OR "Integrative & Complementary Medicine" OR "International Relations" OR "Legal Medicine" OR "Linguistics" OR "Literature" OR "Mathematical & Computational Biology" OR "Mathematical Methods In Social Sciences" OR "Mathematics" OR "Mechanics" OR "Medical Ethics" OR "Medical Informatics" OR "Medical Laboratory Technology" OR "Metallurgy & Metallurgical Engineering" OR "Microscopy" OR "Music" OR "Nuclear Science & Technology" OR "Nursing" OR "Obstetrics & Gynecology" OR "Oncology" OR "Operations Research & Management Science" OR "Ophthalmology" OR "Optics" OR "Orthopedics" OR "Otorhinolaryngology" OR "Pathology" OR "Pediatrics" OR "Philosophy" OR "Physics" OR "Polymer Science" OR "Public Administration" OR "Rehabilitation" OR "Religion" OR "Research & Experimental Medicine" OR "Respiratory System" OR "Rheumatology" OR "Robotics" OR "Social Issues" OR "Social Sciences Other Topics" OR "Social Work" OR "Sociology" OR "Sport Sciences" OR "Substance Abuse" OR "Surgery" OR "Telecommunications" OR "Theater" OR "Thermodynamics" OR "Transplantation" OR "Transportation" OR "Urology & Nephrology" OR "Women's Studies" OR "Anatomy & Morphology" OR "Astronomy & Astrophysics" OR "Biophysics" OR "Cell Biology" OR "Chemistry" OR "Developmental Biology" OR "Endocrinology & Metabolism" OR "Energy & Fuels" OR "Food Science & Technology" OR "Geography" OR "Infectious Diseases" OR "Materials Science" OR "Mineralogy" OR "Mining & Mineral Processing" OR "Neurosciences & Neurology" OR "Nutrition & Dietetics" OR "Paleontology" OR "Parasitology" OR "Pharmacology & Pharmacy" OR "Physical Geography" OR "Psychiatry" OR "Psychology" OR "Public, Environmental & Occupational Health" OR "Radiology, Nuclear Medicine & Medical Imaging" OR "Remote Sensing" OR "Reproductive Biology" OR "Science & Technology Other Topics" OR "Spectroscopy" OR "Tropical Medicine" OR "Urban Studies" OR "Veterinary Sciences" OR "Virology")	NOT TI=("re view" OR "meta-analysis " OR "metaa nalysis" OR "meta analysis ")	937

Ozone	TS=(	("O-3" OR "O3" OR "ozone")	AND	((("arbuscular" OR "vesicular arbuscular" OR "VA" OR "arbutoid" OR "ericoid" OR "ericaceous" OR "monotropoid" OR "orchid" OR "orchidaceous") NEAR/1 ("mycorrhiza" OR "mycorrhizas" OR "mycorrhizal" OR "mycorrhizae" OR "mycorrhization")) OR “ectomycorrhiza” OR “ectomycorrhizas” OR “ectomycorrhizal” OR “ectomycorrhizae” OR “ectomycorrhization” OR "endomycorrhiza” OR endomycorrhizas” OR “endomycorrhizal” OR “endomycorrhizae” OR “endomycorrhization” OR “ectendomycorrhiza” OR “ectendomycorrhizas” OR “ectendomycorrhizal” OR “ectendomycorrhizae” OR “ectendomycorrhization” OR "AM fungi" OR "AM fungus" OR "AM fungal" OR "VAM fungi" OR "VAM fungus" OR "VAM fungal" OR "EcM fungi" OR EcM fungus" OR "EcM fungal" OR "ErM fungi" OR "ErM fungus" OR "ErM fungal" OR (("mycorrhiza" OR "mycorrhizas" OR "mycorrhizal" OR "mycorrhizae" OR "mycorrhization" OR "mycorrhized") AND ("inoculated" OR "inoculation" OR "fungal" OR "fungi" OR "fungus")))	)	NOT SU=("Acoustics" OR "Allergy" OR "Anesthesiology" OR "Anthropology" OR "Archaeology" OR "Architecture" OR "Area Studies" OR "Art" OR "Arts & Humanities-Other Topics" OR "Asian Studies" OR "Audiology & Speech-Language Pathology" OR "Automation & Control Systems" OR "Biomedical Social Sciences" OR "Business & Economics" OR "Cardiovascular System & Cardiology" OR "Classics" OR "Communication" OR "Computer Science" OR "Construction & Building Technology" OR "Criminology & Penology" OR "Critical Care Medicine" OR "Crystallography" OR "Cultural Studies" OR "Dance" OR "Demography" OR "Dentistry, Oral Surgery & Medicine" OR "Dermatology" OR "Development Studies" OR "Education & Educational Research" OR "Electrochemistry" OR "Emergency Medicine" OR "Ethnic Studies" OR "Family Studies" OR "Film, Radio & Television" OR "Gastroenterology & Hepatology" OR "General & Internal Medicine" OR "Geochemistry & Geophysics" OR "Geriatrics & Gerontology" OR "Government & Law" OR "Health Care Sciences & Services" OR "Hematology" OR "History" OR "History & Philosophy of Science" OR "Imaging Science & Photographic Technology" OR "Information Science & Library Science" OR "Instruments & Instrumentation" OR "Integrative & Complementary Medicine" OR "International Relations" OR "Legal Medicine" OR "Linguistics" OR "Literature" OR "Mathematical & Computational Biology" OR "Mathematical Methods In Social Sciences" OR "Mathematics" OR "Mechanics" OR "Medical Ethics" OR "Medical Informatics" OR "Medical Laboratory Technology" OR "Metallurgy & Metallurgical Engineering" OR "Microscopy" OR "Music" OR "Nuclear Science & Technology" OR "Nursing" OR "Obstetrics & Gynecology" OR "Oncology" OR "Operations Research & Management Science" OR "Ophthalmology" OR "Optics" OR "Orthopedics" OR "Otorhinolaryngology" OR "Pathology" OR "Pediatrics" OR "Philosophy" OR "Physics" OR "Polymer Science" OR "Public Administration" OR "Rehabilitation" OR "Religion" OR "Research & Experimental Medicine" OR "Respiratory System" OR "Rheumatology" OR "Robotics" OR "Social Issues" OR "Social Sciences Other Topics" OR "Social Work" OR "Sociology" OR "Sport Sciences" OR "Substance Abuse" OR "Surgery" OR "Telecommunications" OR "Theater" OR "Thermodynamics" OR "Transplantation" OR "Transportation" OR "Urology & Nephrology" OR "Women's Studies" OR "Astronomy & Astrophysics" OR "Biophysics" OR "Biotechnology & Applied Microbiology" OR "Chemistry" OR "Energy & Fuels" OR "Engineering" OR "Geology" OR "Immunology" OR "Materials Science" OR "Mineralogy" OR "Neurosciences & Neurology" OR "Pharmacology & Pharmacy" OR "Public, Environmental & Occupational Health" OR "Spectroscopy")	NOT TI=("review" OR "meta-analysis" OR "metaanalysis" OR "meta analysis")	97
LUC_degradation	TS=(	(("degradation" OR "degraded" OR "conversion" OR "conversions" OR "converted" OR "transformation" OR "transient" OR "transition" OR "transitions") NEAR/5 ("land" OR "lands" OR "landscape" OR "landscapes" OR "grassland" OR "grasslands" OR "cropland" OR "farmland" OR "wetlands" OR "woodland" OR "shrubland" OR "peatlands" OR "rangelands" OR	AND	((("arbuscular" OR "vesicular arbuscular" OR "VA" OR "arbutoid" OR "ericoid" OR "ericaceous" OR "monotropoid" OR "orchid" OR "orchidaceous") NEAR/1 ("mycorrhiza" OR "mycorrhizas" OR "mycorrhizal" OR "mycorrhizae" OR "mycorrhization")) OR “ectomycorrhiza” OR “ectomycorrhizas” OR “ectomycorrhizal” OR “ectomycorrhizae” OR	)	NOT SU=("Acoustics" OR "Allergy" OR "Anesthesiology" OR "Anthropology" OR "Archaeology" OR "Architecture" OR "Area Studies" OR "Art" OR "Arts & Humanities-Other Topics" OR "Asian Studies" OR "Audiology & Speech-Language Pathology" OR "Automation & Control Systems" OR "Biomedical Social Sciences" OR "Business & Economics" OR "Cardiovascular System & Cardiology" OR "Classics" OR "Communication" OR "Computer Science" OR "Construction & Building Technology" OR "Criminology & Penology" OR "Critical Care Medicine" OR "Crystallography" OR "Cultural Studies" OR "Dance" OR "Demography" OR "Dentistry, Oral Surgery & Medicine" OR "Dermatology" OR "Development Studies" OR "Education & Educational Research" OR "Electrochemistry" OR "Emergency Medicine" OR "Ethnic Studies" OR "Family Studies" OR "Film, Radio & Television" OR "Gastroenterology & Hepatology" OR "General & Internal Medicine" OR "Geochemistry & Geophysics" OR	NOT TI=("review" OR "meta-analysis" OR "metaanalysis" OR "meta	46

		"forestland" OR "pastureland" OR "dryland" OR "forest" OR "meadow" OR "microlandscape" OR "microlandscapes" OR "croplands" OR "farmlands" OR "wetland" OR "woodlands" OR "shrublands" OR "peatlands" OR "rangeland" OR "forestlands" OR "pasturelands" OR "drylands" OR "forests" OR "meadows")) AND ((("climate" OR "climatic" OR "global" OR "globally" OR "environment" OR "environments" OR "environmental") AND ("change" OR "changes" OR "changing" OR "changed")) OR "global climate" OR "globa climatic")		“ectomycorrhization” OR "endomycorrhiza” OR endomycorrhizas” OR “endomycorrhizal” OR “endomycorrhizae” OR “endomycorrhization” OR “ectendomycorrhiza” OR “ectendomycorrhizas” OR “ectendomycorrhizal” OR “ectendomycorrhizae” OR “ectendomycorrhization” OR "AM fungi" OR "AM fungus" OR "AM fungal" OR "VAM fungi" OR "VAM fungus" OR "VAM fungal" OR "EcM fungi" OR EcM fungus" OR "EcM fungal" OR "ErM fungi" OR "ErM fungus" OR "ErM fungal" OR (("mycorrhiza" OR "mycorrhizas" OR "mycorrhizal" OR "mycorrhizae" OR "mycorrhization" OR "mycorrhized") AND ("inoculated" OR "inoculation" OR "fungal" OR "fungi" OR "fungus")))		"Geriatrics & Gerontology" OR "Government & Law" OR "Health Care Sciences & Services" OR "Hematology" OR "History" OR "History & Philosophy of Science" OR "Imaging Science & Photographic Technology" OR "Information Science & Library Science" OR "Instruments & Instrumentation" OR "Integrative & Complementary Medicine" OR "International Relations" OR "Legal Medicine" OR "Linguistics" OR "Literature" OR "Mathematical & Computational Biology" OR "Mathematical Methods In Social Sciences" OR "Mathematics" OR "Mechanics" OR "Medical Ethics" OR "Medical Informatics" OR "Medical Laboratory Technology" OR "Metallurgy & Metallurgical Engineering" OR "Microscopy" OR "Music" OR "Nuclear Science & Technology" OR "Nursing" OR "Obstetrics & Gynecology" OR "Oncology" OR "Operations Research & Management Science" OR "Ophthalmology" OR "Optics" OR "Orthopedics" OR "Otorhinolaryngology" OR "Pathology" OR "Pediatrics" OR "Philosophy" OR "Physics" OR "Polymer Science" OR "Public Administration" OR "Rehabilitation" OR "Religion" OR "Research & Experimental Medicine" OR "Respiratory System" OR "Rheumatology" OR "Robotics" OR "Social Issues" OR "Social Sciences Other Topics" OR "Social Work" OR "Sociology" OR "Sport Sciences" OR "Substance Abuse" OR "Surgery" OR "Telecommunications" OR "Theater" OR "Thermodynamics" OR "Transplantation" OR "Transportation" OR "Urology & Nephrology" OR "Women's Studies" OR "Anatomy & Morphology" OR "Astronomy & Astrophysics" OR "Cell Biology" OR "Developmental Biology" OR "Endocrinology & Metabolism" OR "Energy & Fuels" OR "Engineering" OR "Immunology" OR "Infectious Diseases" OR "Materials Science" OR "Meteorology & Atmospheric Sciences" OR "Mineralogy" OR "Mining & Mineral Processing" OR "Neurosciences & Neurology" OR "Nutrition & Dietetics" OR "Paleontology" OR "Parasitology" OR "Pharmacology & Pharmacy" OR "Physiology" OR "Psychiatry" OR "Psychology" OR "Public, Environmental & Occupational Health" OR "Radiology, Nuclear Medicine & Medical Imaging" OR "Remote Sensing" OR "Reproductive Biology" OR "Spectroscopy" OR "Toxicology" OR "Tropical Medicine" OR "Urban Studies" OR "Veterinary Sciences" OR "Virology" OR "Water Resources")	analysis ")	
LUC_land-use change	TS=(	("land use change" OR "land-use change" OR "land cover change" OR "land use changes" OR "land-use changes" OR "land cover changes" OR "land use conversion" OR "land-use conversion" OR "land use conversions" OR "land-use conversions")	AND	((("arbuscular" OR "vesicular arbuscular" OR "VA" OR "arbutoid" OR "ericoid" OR "ericaceous" OR "monotropoid" OR "orchid" OR "orchidaceous") NEAR/1 ("mycorrhiza" OR "mycorrhizas" OR "mycorrhizal" OR "mycorrhizae" OR "mycorrhization")) OR “ectomycorrhiza” OR “ectomycorrhizas” OR “ectomycorrhizal” OR “ectomycorrhizae” OR “ectomycorrhization” OR "endomycorrhiza” OR endomycorrhizas” OR “endomycorrhizal” OR “endomycorrhizae” OR “endomycorrhization” OR	)	NOT SU=("Acoustics" OR "Allergy" OR "Anesthesiology" OR "Anthropology" OR "Archaeology" OR "Architecture" OR "Area Studies" OR "Art" OR "Arts & Humanities-Other Topics" OR "Asian Studies" OR "Audiology & Speech-Language Pathology" OR "Automation & Control Systems" OR "Biomedical Social Sciences" OR "Business & Economics" OR "Cardiovascular System & Cardiology" OR "Classics" OR "Communication" OR "Computer Science" OR "Construction & Building Technology" OR "Criminology & Penology" OR "Critical Care Medicine" OR "Crystallography" OR "Cultural Studies" OR "Dance" OR "Demography" OR "Dentistry, Oral Surgery & Medicine" OR "Dermatology" OR "Development Studies" OR "Education & Educational Research" OR "Electrochemistry" OR "Emergency Medicine" OR "Ethnic Studies" OR "Family Studies" OR "Film, Radio & Television" OR "Gastroenterology & Hepatology" OR "General & Internal Medicine" OR "Geochemistry & Geophysics" OR "Geriatrics & Gerontology" OR "Government & Law" OR "Health Care Sciences & Services" OR "Hematology" OR "History" OR "History & Philosophy of Science" OR "Imaging Science & Photographic Technology" OR "Information Science & Library Science" OR "Instruments & Instrumentation" OR "Integrative & Complementary Medicine" OR "International Relations" OR "Legal Medicine" OR "Linguistics" OR "Literature" OR "Mathematical & Computational Biology" OR "Mathematical	NOT TI=("review" OR "meta-analysis" OR "metaanalysis" OR "meta analysis")	112

				“ectendomycorrhiza” OR “ectendomycorrhizas” OR “ectendomycorrhizal” OR “ectendomycorrhizae” OR “ectendomycorrhization” OR "AM fungi" OR "AM fungus" OR "AM fungal" OR "VAM fungi" OR "VAM fungus" OR "VAM fungal" OR "EcM fungi" OR EcM fungus" OR "EcM fungal" OR "ErM fungi" OR "ErM fungus" OR "ErM fungal" OR (("mycorrhiza" OR "mycorrhizas" OR "mycorrhizal" OR "mycorrhizae" OR "mycorrhization" OR "mycorrhized") AND ("inoculated" OR "inoculation" OR "fungal" OR "fungi" OR "fungus")))		Methods In Social Sciences" OR "Mathematics" OR "Mechanics" OR "Medical Ethics" OR "Medical Informatics" OR "Medical Laboratory Technology" OR "Metallurgy & Metallurgical Engineering" OR "Microscopy" OR "Music" OR "Nuclear Science & Technology" OR "Nursing" OR "Obstetrics & Gynecology" OR "Oncology" OR "Operations Research & Management Science" OR "Ophthalmology" OR "Optics" OR "Orthopedics" OR "Otorhinolaryngology" OR "Pathology" OR "Pediatrics" OR "Philosophy" OR "Physics" OR "Polymer Science" OR "Public Administration" OR "Rehabilitation" OR "Religion" OR "Research & Experimental Medicine" OR "Respiratory System" OR "Rheumatology" OR "Robotics" OR "Social Issues" OR "Social Sciences Other Topics" OR "Social Work" OR "Sociology" OR "Sport Sciences" OR "Substance Abuse" OR "Surgery" OR "Telecommunications" OR "Theater" OR "Thermodynamics" OR "Transplantation" OR "Transportation" OR "Urology & Nephrology" OR "Women's Studies" OR "Anatomy & Morphology" OR "Astronomy & Astrophysics" OR "Cell Biology" OR "Developmental Biology" OR "Endocrinology & Metabolism" OR "Energy & Fuels" OR "Engineering" OR "Immunology" OR "Infectious Diseases" OR "Materials Science" OR "Meteorology & Atmospheric Sciences" OR "Mineralogy" OR "Mining & Mineral Processing" OR "Neurosciences & Neurology" OR "Nutrition & Dietetics" OR "Paleontology" OR "Parasitology" OR "Pharmacology & Pharmacy" OR "Physiology" OR "Psychiatry" OR "Psychology" OR "Public, Environmental & Occupational Health" OR "Radiology, Nuclear Medicine & Medical Imaging" OR "Remote Sensing" OR "Reproductive Biology" OR "Spectroscopy" OR "Toxicology" OR "Tropical Medicine" OR "Urban Studies" OR "Veterinary Sciences" OR "Virology" OR "Water Resources")		
LUC_fragmentation	TS=(	(("habitat" OR "landscape" OR "microlandscape" OR "habitats" OR "landscapes" OR "microlandscapes") NEAR/2 ("loss" OR "losses" OR "fragmentation" OR "fragmentations" OR "fragmented" OR "patchiness" OR "patch size" OR "patch" OR "patches" OR "degraded" OR "degradation" OR "connectivity" OR "connected" OR "corridor" OR "corridors"))	AND	((("arbuscular" OR "vesicular arbuscular" OR "VA" OR "arbutoid" OR "ericoid" OR "ericaceous" OR "monotropoid" OR "orchid" OR "orchidaceous") NEAR/1 ("mycorrhiza" OR "mycorrhizas" OR "mycorrhizal" OR "mycorrhizae" OR "mycorrhization")) OR “ectomycorrhiza” OR “ectomycorrhizas” OR “ectomycorrhizal” OR “ectomycorrhizae” OR “ectomycorrhization” OR "endomycorrhiza” OR endomycorrhizas” OR “endomycorrhizal” OR “endomycorrhizae” OR “endomycorrhization” OR “ectendomycorrhiza” OR “ectendomycorrhizas” OR “ectendomycorrhizal” OR “ectendomycorrhizae” OR “ectendomycorrhization” OR "AM fungi" OR "AM fungus" OR "AM	)	NOT SU=("Acoustics" OR "Allergy" OR "Anesthesiology" OR "Anthropology" OR "Archaeology" OR "Architecture" OR "Area Studies" OR "Art" OR "Arts & Humanities-Other Topics" OR "Asian Studies" OR "Audiology & Speech-Language Pathology" OR "Automation & Control Systems" OR "Biomedical Social Sciences" OR "Business & Economics" OR "Cardiovascular System & Cardiology" OR "Classics" OR "Communication" OR "Computer Science" OR "Construction & Building Technology" OR "Criminology & Penology" OR "Critical Care Medicine" OR "Crystallography" OR "Cultural Studies" OR "Dance" OR "Demography" OR "Dentistry, Oral Surgery & Medicine" OR "Dermatology" OR "Development Studies" OR "Education & Educational Research" OR "Electrochemistry" OR "Emergency Medicine" OR "Ethnic Studies" OR "Family Studies" OR "Film, Radio & Television" OR "Gastroenterology & Hepatology" OR "General & Internal Medicine" OR "Geochemistry & Geophysics" OR "Geriatrics & Gerontology" OR "Government & Law" OR "Health Care Sciences & Services" OR "Hematology" OR "History" OR "History & Philosophy of Science" OR "Imaging Science & Photographic Technology" OR "Information Science & Library Science" OR "Instruments & Instrumentation" OR "Integrative & Complementary Medicine" OR "International Relations" OR "Legal Medicine" OR "Linguistics" OR "Literature" OR "Mathematical & Computational Biology" OR "Mathematical Methods In Social Sciences" OR "Mathematics" OR "Mechanics" OR "Medical Ethics" OR "Medical Informatics" OR "Medical Laboratory Technology" OR "Metallurgy & Metallurgical Engineering" OR "Microscopy" OR "Music" OR "Nuclear Science & Technology" OR "Nursing" OR "Obstetrics & Gynecology" OR "Oncology" OR "Operations Research & Management Science" OR "Ophthalmology" OR "Optics" OR "Orthopedics" OR "Otorhinolaryngology" OR "Pathology" OR "Pediatrics" OR	NOT TI=("review" OR "meta-analysis" OR "metaanalysis" OR "meta analysis")	90

				fungal" OR "VAM fungi" OR "VAM fungus" OR "VAM fungal" OR "EcM fungi" OR EcM fungus" OR "EcM fungal" OR "ErM fungi" OR "ErM fungus" OR "ErM fungal" OR (("mycorrhiza" OR "mycorrhizas" OR "mycorrhizal" OR "mycorrhizae" OR "mycorrhization" OR "mycorrhized") AND ("inoculated" OR "inoculation" OR "fungal" OR "fungi" OR "fungus")))		"Philosophy" OR "Physics" OR "Polymer Science" OR "Public Administration" OR "Rehabilitation" OR "Religion" OR "Research & Experimental Medicine" OR "Respiratory System" OR "Rheumatology" OR "Robotics" OR "Social Issues" OR "Social Sciences Other Topics" OR "Social Work" OR "Sociology" OR "Sport Sciences" OR "Substance Abuse" OR "Surgery" OR "Telecommunications" OR "Theater" OR "Thermodynamics" OR "Transplantation" OR "Transportation" OR "Urology & Nephrology" OR "Women's Studies" OR "Anatomy & Morphology" OR "Astronomy & Astrophysics" OR "Cell Biology" OR "Developmental Biology" OR "Endocrinology & Metabolism" OR "Energy & Fuels" OR "Engineering" OR "Immunology" OR "Infectious Diseases" OR "Materials Science" OR "Meteorology & Atmospheric Sciences" OR "Mineralogy" OR "Mining & Mineral Processing" OR "Neurosciences & Neurology" OR "Nutrition & Dietetics" OR "Paleontology" OR "Parasitology" OR "Pharmacology & Pharmacy" OR "Physiology" OR "Psychiatry" OR "Psychology" OR "Public, Environmental & Occupational Health" OR "Radiology, Nuclear Medicine & Medical Imaging" OR "Remote Sensing" OR "Reproductive Biology" OR "Spectroscopy" OR "Toxicology" OR "Tropical Medicine" OR "Urban Studies" OR "Veterinary Sciences" OR "Virology" OR "Water Resources")		
UVB	TS=(	((("UV-B" OR "UVB" OR "UV B" OR "(UVB)" OR "(UV-B)" OR "ultraviolet-B" OR "ultraviolet" OR "midultraviolet") AND ("radiation" OR "radiations" OR "radiance" OR "radiances" OR "radiated" OR "irradiance" OR "irradiances" OR "irradiation" OR "irradiations" OR "irradiated")) OR (("stratospheric ozone") AND ("reduction" OR "depletion" OR "reduced" OR "depleted")))	AND	((("arbuscular" OR "vesicular arbuscular" OR "VA" OR "arbutoid" OR "ericoid" OR "ericaceous" OR "monotropoid" OR "orchid" OR "orchidaceous") NEAR/1 ("mycorrhiza" OR "mycorrhizas" OR "mycorrhizal" OR "mycorrhizae" OR "mycorrhization")) OR “ectomycorrhiza” OR “ectomycorrhizas” OR “ectomycorrhizal” OR “ectomycorrhizae” OR “ectomycorrhization” OR "endomycorrhiza” OR endomycorrhizas” OR “endomycorrhizal” OR “endomycorrhizae” OR “endomycorrhization” OR “ectendomycorrhiza” OR “ectendomycorrhizas” OR “ectendomycorrhizal” OR “ectendomycorrhizae” OR “ectendomycorrhization” OR "AM fungi" OR "AM fungus" OR "AM fungal" OR "VAM fungi" OR "VAM fungus" OR "VAM fungal" OR "EcM fungi" OR EcM fungus" OR "EcM fungal" OR "ErM fungi" OR "ErM fungus" OR "ErM fungal" OR (("mycorrhiza" OR "mycorrhizas" OR	)	NOT SU=("Acoustics" OR "Allergy" OR "Anesthesiology" OR "Anthropology" OR "Archaeology" OR "Architecture" OR "Area Studies" OR "Art" OR "Arts & Humanities-Other Topics" OR "Asian Studies" OR "Audiology & Speech-Language Pathology" OR "Automation & Control Systems" OR "Biomedical Social Sciences" OR "Business & Economics" OR "Cardiovascular System & Cardiology" OR "Classics" OR "Communication" OR "Computer Science" OR "Construction & Building Technology" OR "Criminology & Penology" OR "Critical Care Medicine" OR "Crystallography" OR "Cultural Studies" OR "Dance" OR "Demography" OR "Dentistry, Oral Surgery & Medicine" OR "Dermatology" OR "Development Studies" OR "Education & Educational Research" OR "Electrochemistry" OR "Emergency Medicine" OR "Ethnic Studies" OR "Family Studies" OR "Film, Radio & Television" OR "Gastroenterology & Hepatology" OR "General & Internal Medicine" OR "Geochemistry & Geophysics" OR "Geriatrics & Gerontology" OR "Government & Law" OR "Health Care Sciences & Services" OR "Hematology" OR "History" OR "History & Philosophy of Science" OR "Imaging Science & Photographic Technology" OR "Information Science & Library Science" OR "Instruments & Instrumentation" OR "Integrative & Complementary Medicine" OR "International Relations" OR "Legal Medicine" OR "Linguistics" OR "Literature" OR "Mathematical & Computational Biology" OR "Mathematical Methods In Social Sciences" OR "Mathematics" OR "Mechanics" OR "Medical Ethics" OR "Medical Informatics" OR "Medical Laboratory Technology" OR "Metallurgy & Metallurgical Engineering" OR "Microscopy" OR "Music" OR "Nuclear Science & Technology" OR "Nursing" OR "Obstetrics & Gynecology" OR "Oncology" OR "Operations Research & Management Science" OR "Ophthalmology" OR "Optics" OR "Orthopedics" OR "Otorhinolaryngology" OR "Pathology" OR "Pediatrics" OR "Philosophy" OR "Physics" OR "Polymer Science" OR "Public Administration" OR "Rehabilitation" OR "Religion" OR "Research & Experimental Medicine" OR "Respiratory System" OR "Rheumatology" OR "Robotics" OR "Social Issues" OR "Social Sciences Other Topics" OR "Social Work" OR "Sociology" OR "Sport Sciences" OR "Substance Abuse" OR "Surgery" OR "Telecommunications" OR "Theater" OR "Thermodynamics" OR "Transplantation" OR "Transportation" OR "Urology &	NOT TI=("review" OR "meta-analysis" OR "metaanalysis" OR "meta analysis")	18

				"mycorrhizal" OR "mycorrhizae" OR "mycorrhization" OR "mycorrhized") AND ("inoculated" OR "inoculation" OR "fungal" OR "fungi" OR "fungus")))		Nephrology" OR "Women's Studies" OR "Astronomy & Astrophysics" OR "Immunology" OR "Materials Science" OR "Pharmacology & Pharmacy" OR "Psychiatry" OR "Public, Environmental & Occupational Health" OR "Radiology, Nuclear Medicine & Medical Imaging" OR "Spectroscopy")		
SynChem_surfactants	TS=(	("fluorosurfactant" OR "fluorosurfactants" OR "surfactant" OR "surfactants" OR “surface active agent” OR “surface active agents” OR “surface active chemical” OR “surface active chemicals” OR “synthetic detergent” OR “synthetic detergents”)	AND	((("arbuscular" OR "vesicular arbuscular" OR "VA" OR "arbutoid" OR "ericoid" OR "ericaceous" OR "monotropoid" OR "orchid" OR "orchidaceous") NEAR/1 ("mycorrhiza" OR "mycorrhizas" OR "mycorrhizal" OR "mycorrhizae" OR "mycorrhization")) OR “ectomycorrhiza” OR “ectomycorrhizas” OR “ectomycorrhizal” OR “ectomycorrhizae” OR “ectomycorrhization” OR "endomycorrhiza” OR endomycorrhizas” OR “endomycorrhizal” OR “endomycorrhizae” OR “endomycorrhization” OR “ectendomycorrhiza” OR “ectendomycorrhizas” OR “ectendomycorrhizal” OR “ectendomycorrhizae” OR “ectendomycorrhization” OR "AM fungi" OR "AM fungus" OR "AM fungal" OR "VAM fungi" OR "VAM fungus" OR "VAM fungal" OR "EcM fungi" OR EcM fungus" OR "EcM fungal" OR "ErM fungi" OR "ErM fungus" OR "ErM fungal" OR (("mycorrhiza" OR "mycorrhizas" OR "mycorrhizal" OR "mycorrhizae" OR "mycorrhization" OR "mycorrhized") AND ("inoculated" OR "inoculation" OR "fungal" OR "fungi" OR "fungus")))	)	NOT SU=("Acoustics" OR "Allergy" OR "Anesthesiology" OR "Anthropology" OR "Archaeology" OR "Architecture" OR "Area Studies" OR "Art" OR "Arts & Humanities-Other Topics" OR "Asian Studies" OR "Audiology & Speech-Language Pathology" OR "Automation & Control Systems" OR "Biomedical Social Sciences" OR "Business & Economics" OR "Cardiovascular System & Cardiology" OR "Classics" OR "Communication" OR "Computer Science" OR "Construction & Building Technology" OR "Criminology & Penology" OR "Critical Care Medicine" OR "Crystallography" OR "Cultural Studies" OR "Dance" OR "Demography" OR "Dentistry, Oral Surgery & Medicine" OR "Dermatology" OR "Development Studies" OR "Education & Educational Research" OR "Electrochemistry" OR "Emergency Medicine" OR "Ethnic Studies" OR "Family Studies" OR "Film, Radio & Television" OR "Gastroenterology & Hepatology" OR "General & Internal Medicine" OR "Geochemistry & Geophysics" OR "Geriatrics & Gerontology" OR "Government & Law" OR "Health Care Sciences & Services" OR "Hematology" OR "History" OR "History & Philosophy of Science" OR "Imaging Science & Photographic Technology" OR "Information Science & Library Science" OR "Instruments & Instrumentation" OR "Integrative & Complementary Medicine" OR "International Relations" OR "Legal Medicine" OR "Linguistics" OR "Literature" OR "Mathematical & Computational Biology" OR "Mathematical Methods In Social Sciences" OR "Mathematics" OR "Mechanics" OR "Medical Ethics" OR "Medical Informatics" OR "Medical Laboratory Technology" OR "Metallurgy & Metallurgical Engineering" OR "Microscopy" OR "Music" OR "Nuclear Science & Technology" OR "Nursing" OR "Obstetrics & Gynecology" OR "Oncology" OR "Operations Research & Management Science" OR "Ophthalmology" OR "Optics" OR "Orthopedics" OR "Otorhinolaryngology" OR "Pathology" OR "Pediatrics" OR "Philosophy" OR "Physics" OR "Polymer Science" OR "Public Administration" OR "Rehabilitation" OR "Religion" OR "Research & Experimental Medicine" OR "Respiratory System" OR "Rheumatology" OR "Robotics" OR "Social Issues" OR "Social Sciences Other Topics" OR "Social Work" OR "Sociology" OR "Sport Sciences" OR "Substance Abuse" OR "Surgery" OR "Telecommunications" OR "Theater" OR "Thermodynamics" OR "Transplantation" OR "Transportation" OR "Urology & Nephrology" OR "Women's Studies" OR "Biochemistry & Molecular Biology" OR "Cell Biology" OR "Endocrinology & Metabolism" OR "Energy & Fuels" OR "Genetics & Heredity" OR "Materials Science" OR "Mineralogy" OR "Mining & Mineral Processing" OR "Physiology" OR "Psychiatry" OR "Radiology, Nuclear Medicine & Medical Imaging" OR "Spectroscopy")	NOT TI=("review" OR "meta-analysis" OR "metaanalysis" OR "meta analysis")	29
SynChem_fluoro	TS=(	(("fluorinated" OR "perfluorinated" OR "polyfluorinated" OR "fluoroalkyl" OR "perfluoroalkyl" OR "polyfluoroalkyl") AND ("acid"	AND	((("arbuscular" OR "vesicular arbuscular" OR "VA" OR "arbutoid" OR "ericoid" OR "ericaceous" OR "monotropoid" OR "orchid" OR "orchidaceous") NEAR/1 ("mycorrhiza" OR "mycorrhizas" OR	)	NOT SU=("Acoustics" OR "Allergy" OR "Anesthesiology" OR "Anthropology" OR "Archaeology" OR "Architecture" OR "Area Studies" OR "Art" OR "Arts & Humanities-Other Topics" OR "Asian Studies" OR "Audiology & Speech-Language Pathology" OR "Automation & Control Systems" OR "Biomedical Social Sciences" OR "Business & Economics" OR "Cardiovascular System & Cardiology" OR "Classics" OR "Communication" OR "Computer Science" OR "Construction & Building Technology"	NOT TI=("review" OR "meta-analysis	2

		OR "acids"OR "substance" OR "substances" OR "compound" OR "compounds" OR "chemical" OR "chemicals"))		"mycorrhizal" OR "mycorrhizae" OR "mycorrhization")) OR “ectomycorrhiza” OR “ectomycorrhizas” OR “ectomycorrhizal” OR “ectomycorrhizae” OR “ectomycorrhization” OR "endomycorrhiza” OR endomycorrhizas” OR “endomycorrhizal” OR “endomycorrhizae” OR “endomycorrhization” OR “ectendomycorrhiza” OR “ectendomycorrhizas” OR “ectendomycorrhizal” OR “ectendomycorrhizae” OR “ectendomycorrhization” OR "AM fungi" OR "AM fungus" OR "AM fungal" OR "VAM fungi" OR "VAM fungus" OR "VAM fungal" OR "EcM fungi" OR EcM fungus" OR "EcM fungal" OR "ErM fungi" OR "ErM fungus" OR "ErM fungal" OR (("mycorrhiza" OR "mycorrhizas" OR "mycorrhizal" OR "mycorrhizae" OR "mycorrhization" OR "mycorrhized") AND ("inoculated" OR "inoculation" OR "fungal" OR "fungi" OR "fungus")))		OR "Criminology & Penology" OR "Critical Care Medicine" OR "Crystallography" OR "Cultural Studies" OR "Dance" OR "Demography" OR "Dentistry, Oral Surgery & Medicine" OR "Dermatology" OR "Development Studies" OR "Education & Educational Research" OR "Electrochemistry" OR "Emergency Medicine" OR "Ethnic Studies" OR "Family Studies" OR "Film, Radio & Television" OR "Gastroenterology & Hepatology" OR "General & Internal Medicine" OR "Geochemistry & Geophysics" OR "Geriatrics & Gerontology" OR "Government & Law" OR "Health Care Sciences & Services" OR "Hematology" OR "History" OR "History & Philosophy of Science" OR "Imaging Science & Photographic Technology" OR "Information Science & Library Science" OR "Instruments & Instrumentation" OR "Integrative & Complementary Medicine" OR "International Relations" OR "Legal Medicine" OR "Linguistics" OR "Literature" OR "Mathematical & Computational Biology" OR "Mathematical Methods In Social Sciences" OR "Mathematics" OR "Mechanics" OR "Medical Ethics" OR "Medical Informatics" OR "Medical Laboratory Technology" OR "Metallurgy & Metallurgical Engineering" OR "Microscopy" OR "Music" OR "Nuclear Science & Technology" OR "Nursing" OR "Obstetrics & Gynecology" OR "Oncology" OR "Operations Research & Management Science" OR "Ophthalmology" OR "Optics" OR "Orthopedics" OR "Otorhinolaryngology" OR "Pathology" OR "Pediatrics" OR "Philosophy" OR "Physics" OR "Polymer Science" OR "Public Administration" OR "Rehabilitation" OR "Religion" OR "Research & Experimental Medicine" OR "Respiratory System" OR "Rheumatology" OR "Robotics" OR "Social Issues" OR "Social Sciences Other Topics" OR "Social Work" OR "Sociology" OR "Sport Sciences" OR "Substance Abuse" OR "Surgery" OR "Telecommunications" OR "Theater" OR "Thermodynamics" OR "Transplantation" OR "Transportation" OR "Urology & Nephrology" OR "Women's Studies" OR "Biochemistry & Molecular Biology" OR "Cell Biology" OR "Endocrinology & Metabolism" OR "Energy & Fuels" OR "Genetics & Heredity" OR "Materials Science" OR "Mineralogy" OR "Mining & Mineral Processing" OR "Physiology" OR "Psychiatry" OR "Radiology, Nuclear Medicine & Medical Imaging" OR "Spectroscopy")	" OR "metaanalysis" OR "meta analysis ")	
MP	TS=(	("micro-plastic" OR "micro-plastics" OR "microplastic" OR "microplastics")	AND	((("arbuscular" OR "vesicular arbuscular" OR "VA" OR "arbutoid" OR "ericoid" OR "ericaceous" OR "monotropoid" OR "orchid" OR "orchidaceous") NEAR/1 ("mycorrhiza" OR "mycorrhizas" OR "mycorrhizal" OR "mycorrhizae" OR "mycorrhization")) OR “ectomycorrhiza” OR “ectomycorrhizas” OR “ectomycorrhizal” OR “ectomycorrhizae” OR “ectomycorrhization” OR "endomycorrhiza” OR endomycorrhizas” OR “endomycorrhizal” OR “endomycorrhizae” OR “endomycorrhization” OR	)	NOT SU=("Acoustics" OR "Allergy" OR "Anesthesiology" OR "Anthropology" OR "Archaeology" OR "Architecture" OR "Area Studies" OR "Art" OR "Arts & Humanities-Other Topics" OR "Asian Studies" OR "Audiology & Speech-Language Pathology" OR "Automation & Control Systems" OR "Biomedical Social Sciences" OR "Business & Economics" OR "Cardiovascular System & Cardiology" OR "Classics" OR "Communication" OR "Computer Science" OR "Construction & Building Technology" OR "Criminology & Penology" OR "Critical Care Medicine" OR "Crystallography" OR "Cultural Studies" OR "Dance" OR "Demography" OR "Dentistry, Oral Surgery & Medicine" OR "Dermatology" OR "Development Studies" OR "Education & Educational Research" OR "Electrochemistry" OR "Emergency Medicine" OR "Ethnic Studies" OR "Family Studies" OR "Film, Radio & Television" OR "Gastroenterology & Hepatology" OR "General & Internal Medicine" OR "Geochemistry & Geophysics" OR "Geriatrics & Gerontology" OR "Government & Law" OR "Health Care Sciences & Services" OR "Hematology" OR "History" OR "History & Philosophy of Science" OR "Imaging Science & Photographic Technology" OR "Information Science & Library Science" OR "Instruments & Instrumentation" OR "Integrative & Complementary Medicine" OR "International Relations" OR "Legal Medicine" OR "Linguistics" OR "Literature" OR "Mathematical & Computational Biology" OR "Mathematical	NOT TI=("review" OR "meta-analysis " OR "metaanalysis" OR "meta analysis ")	2

				“ectendomycorrhiza” OR “ectendomycorrhizas” OR “ectendomycorrhizal” OR “ectendomycorrhizae” OR “ectendomycorrhization” OR "AM fungi" OR "AM fungus" OR "AM fungal" OR "VAM fungi" OR "VAM fungus" OR "VAM fungal" OR "EcM fungi" OR EcM fungus" OR "EcM fungal" OR "ErM fungi" OR "ErM fungus" OR "ErM fungal" OR (("mycorrhiza" OR "mycorrhizas" OR "mycorrhizal" OR "mycorrhizae" OR "mycorrhization" OR "mycorrhized") AND ("inoculated" OR "inoculation" OR "fungal" OR "fungi" OR "fungus")))		Methods In Social Sciences" OR "Mathematics" OR "Mechanics" OR "Medical Ethics" OR "Medical Informatics" OR "Medical Laboratory Technology" OR "Metallurgy & Metallurgical Engineering" OR "Microscopy" OR "Music" OR "Nuclear Science & Technology" OR "Nursing" OR "Obstetrics & Gynecology" OR "Oncology" OR "Operations Research & Management Science" OR "Ophthalmology" OR "Optics" OR "Orthopedics" OR "Otorhinolaryngology" OR "Pathology" OR "Pediatrics" OR "Philosophy" OR "Physics" OR "Polymer Science" OR "Public Administration" OR "Rehabilitation" OR "Religion" OR "Research & Experimental Medicine" OR "Respiratory System" OR "Rheumatology" OR "Robotics" OR "Social Issues" OR "Social Sciences Other Topics" OR "Social Work" OR "Sociology" OR "Sport Sciences" OR "Substance Abuse" OR "Surgery" OR "Telecommunications" OR "Theater" OR "Thermodynamics" OR "Transplantation" OR "Transportation" OR "Urology & Nephrology" OR "Women's Studies" OR "Psychiatry")		
Over_overexploitation	TS=(	(("overexploitation" OR "overexploited" OR "over-used" OR "overused" OR "overuse" OR "over-use" OR "overutilization" OR "overutilisation" OR "overutilized" OR "overfishing" OR "overgrazing") AND ("experiment" OR "experiments" OR "experimental" OR "experimentally" OR "simulated" OR "treatment" OR "treatments"))	AND	((("arbuscular" OR "vesicular arbuscular" OR "VA" OR "arbutoid" OR "ericoid" OR "ericaceous" OR "monotropoid" OR "orchid" OR "orchidaceous") NEAR/1 ("mycorrhiza" OR "mycorrhizas" OR "mycorrhizal" OR "mycorrhizae" OR "mycorrhization")) OR “ectomycorrhiza” OR “ectomycorrhizas” OR “ectomycorrhizal” OR “ectomycorrhizae” OR “ectomycorrhization” OR "endomycorrhiza” OR endomycorrhizas” OR “endomycorrhizal” OR “endomycorrhizae” OR “endomycorrhization” OR “ectendomycorrhiza” OR “ectendomycorrhizas” OR “ectendomycorrhizal” OR “ectendomycorrhizae” OR “ectendomycorrhization” OR "AM fungi" OR "AM fungus" OR "AM fungal" OR "VAM fungi" OR "VAM fungus" OR "VAM fungal" OR "EcM fungi" OR EcM fungus" OR "EcM fungal" OR "ErM fungi" OR "ErM fungus" OR "ErM fungal" OR (("mycorrhiza" OR "mycorrhizas" OR	)	NOT SU=("Acoustics" OR "Allergy" OR "Anesthesiology" OR "Anthropology" OR "Archaeology" OR "Architecture" OR "Area Studies" OR "Art" OR "Arts & Humanities-Other Topics" OR "Asian Studies" OR "Audiology & Speech-Language Pathology" OR "Automation & Control Systems" OR "Biomedical Social Sciences" OR "Business & Economics" OR "Cardiovascular System & Cardiology" OR "Classics" OR "Communication" OR "Computer Science" OR "Construction & Building Technology" OR "Criminology & Penology" OR "Critical Care Medicine" OR "Crystallography" OR "Cultural Studies" OR "Dance" OR "Demography" OR "Dentistry, Oral Surgery & Medicine" OR "Dermatology" OR "Development Studies" OR "Education & Educational Research" OR "Electrochemistry" OR "Emergency Medicine" OR "Ethnic Studies" OR "Family Studies" OR "Film, Radio & Television" OR "Gastroenterology & Hepatology" OR "General & Internal Medicine" OR "Geochemistry & Geophysics" OR "Geriatrics & Gerontology" OR "Government & Law" OR "Health Care Sciences & Services" OR "Hematology" OR "History" OR "History & Philosophy of Science" OR "Imaging Science & Photographic Technology" OR "Information Science & Library Science" OR "Instruments & Instrumentation" OR "Integrative & Complementary Medicine" OR "International Relations" OR "Legal Medicine" OR "Linguistics" OR "Literature" OR "Mathematical & Computational Biology" OR "Mathematical Methods In Social Sciences" OR "Mathematics" OR "Mechanics" OR "Medical Ethics" OR "Medical Informatics" OR "Medical Laboratory Technology" OR "Metallurgy & Metallurgical Engineering" OR "Microscopy" OR "Music" OR "Nuclear Science & Technology" OR "Nursing" OR "Obstetrics & Gynecology" OR "Oncology" OR "Operations Research & Management Science" OR "Ophthalmology" OR "Optics" OR "Orthopedics" OR "Otorhinolaryngology" OR "Pathology" OR "Pediatrics" OR "Philosophy" OR "Physics" OR "Polymer Science" OR "Public Administration" OR "Rehabilitation" OR "Religion" OR "Research & Experimental Medicine" OR "Respiratory System" OR "Rheumatology" OR "Robotics" OR "Social Issues" OR "Social Sciences Other Topics" OR "Social Work" OR "Sociology" OR "Sport Sciences" OR "Substance Abuse" OR "Surgery" OR "Telecommunications" OR "Theater" OR "Thermodynamics" OR "Transplantation" OR "Transportation" OR "Urology &	NOT TI=("review" OR "meta-analysis" OR "metaanalysis" OR "meta analysis")	10

New Phytologist

				"mycorrhizal" OR "mycorrhizae" OR "mycorrhization" OR "mycorrhized") AND ("inoculated" OR "inoculation" OR "fungal" OR "fungi" OR "fungus")))		Nephrology" OR "Women's Studies" OR "Biochemistry & Molecular Biology" OR "Cell Biology" OR "Chemistry" OR "Endocrinology & Metabolism" OR "Food Science & Technology" OR "Genetics & Heredity" OR "Infectious Diseases" OR "Materials Science" OR "Neurosciences & Neurology" OR "Nutrition & Dietetics" OR "Pharmacology & Pharmacy" OR "Physiology" OR "Psychiatry" OR "Psychology" OR "Public, Environmental & Occupational Health" OR "Radiology, Nuclear Medicine & Medical Imaging" OR "Remote Sensing" OR "Reproductive Biology" OR "Toxicology" OR "Veterinary Sciences")		
Over_clear cutting	TS=(	(("cutting" OR "clearcutting" OR "clear-cutting" OR "clear-cut" OR "biomass removal" OR "harvest" OR "clearfelling" OR "clear felling" OR "treecutting" OR "tree cutting") NEAR/15 ("forest" OR "forests" OR "forestry" OR "timber" OR "overstory" OR "understory" OR "trees"))	AND	((("arbuscular" OR "vesicular arbuscular" OR "VA" OR "arbutoid" OR "ericoid" OR "ericaceous" OR "monotropoid" OR "orchid" OR "orchidaceous") NEAR/1 ("mycorrhiza" OR "mycorrhizas" OR "mycorrhizal" OR "mycorrhizae" OR "mycorrhization")) OR “ectomycorrhiza” OR “ectomycorrhizas” OR “ectomycorrhizal” OR “ectomycorrhizae” OR “ectomycorrhization” OR "endomycorrhiza" OR endomycorrhizas” OR “endomycorrhizal” OR “endomycorrhizae” OR “endomycorrhization” OR “ectendomycorrhiza” OR “ectendomycorrhizas” OR “ectendomycorrhizal” OR “ectendomycorrhizae” OR “ectendomycorrhization” OR "AM fungi" OR "AM fungus" OR "AM fungal" OR "VAM fungi" OR "VAM fungus" OR "VAM fungal" OR "EcM fungi" OR EcM fungus" OR "EcM fungal" OR "ErM fungi" OR "ErM fungus" OR "ErM fungal" OR (("mycorrhiza" OR "mycorrhizas" OR "mycorrhizal" OR "mycorrhizae" OR "mycorrhization" OR "mycorrhized") AND ("inoculated" OR "inoculation" OR "fungal" OR "fungi" OR "fungus")))	)	NOT SU=("Acoustics" OR "Allergy" OR "Anesthesiology" OR "Anthropology" OR "Archaeology" OR "Architecture" OR "Area Studies" OR "Art" OR "Arts & Humanities-Other Topics" OR "Asian Studies" OR "Audiology & Speech-Language Pathology" OR "Automation & Control Systems" OR "Biomedical Social Sciences" OR "Business & Economics" OR "Cardiovascular System & Cardiology" OR "Classics" OR "Communication" OR "Computer Science" OR "Construction & Building Technology" OR "Criminology & Penology" OR "Critical Care Medicine" OR "Crystallography" OR "Cultural Studies" OR "Dance" OR "Demography" OR "Dentistry, Oral Surgery & Medicine" OR "Dermatology" OR "Development Studies" OR "Education & Educational Research" OR "Electrochemistry" OR "Emergency Medicine" OR "Ethnic Studies" OR "Family Studies" OR "Film, Radio & Television" OR "Gastroenterology & Hepatology" OR "General & Internal Medicine" OR "Geochemistry & Geophysics" OR "Geriatrics & Gerontology" OR "Government & Law" OR "Health Care Sciences & Services" OR "Hematology" OR "History" OR "History & Philosophy of Science" OR "Imaging Science & Photographic Technology" OR "Information Science & Library Science" OR "Instruments & Instrumentation" OR "Integrative & Complementary Medicine" OR "International Relations" OR "Legal Medicine" OR "Linguistics" OR "Literature" OR "Mathematical & Computational Biology" OR "Mathematical Methods In Social Sciences" OR "Mathematics" OR "Mechanics" OR "Medical Ethics" OR "Medical Informatics" OR "Medical Laboratory Technology" OR "Metallurgy & Metallurgical Engineering" OR "Microscopy" OR "Music" OR "Nuclear Science & Technology" OR "Nursing" OR "Obstetrics & Gynecology" OR "Oncology" OR "Operations Research & Management Science" OR "Ophthalmology" OR "Optics" OR "Orthopedics" OR "Otorhinolaryngology" OR "Pathology" OR "Pediatrics" OR "Philosophy" OR "Physics" OR "Polymer Science" OR "Public Administration" OR "Rehabilitation" OR "Religion" OR "Research & Experimental Medicine" OR "Respiratory System" OR "Rheumatology" OR "Robotics" OR "Social Issues" OR "Social Sciences Other Topics" OR "Social Work" OR "Sociology" OR "Sport Sciences" OR "Substance Abuse" OR "Surgery" OR "Telecommunications" OR "Theater" OR "Thermodynamics" OR "Transplantation" OR "Transportation" OR "Urology & Nephrology" OR "Women's Studies" OR "Biochemistry & Molecular Biology" OR "Cell Biology" OR "Chemistry" OR "Endocrinology & Metabolism" OR "Food Science & Technology" OR "Genetics & Heredity" OR "Infectious Diseases" OR "Materials Science" OR "Neurosciences & Neurology" OR "Nutrition & Dietetics" OR "Pharmacology & Pharmacy" OR "Physiology" OR "Psychiatry" OR "Psychology" OR "Public, Environmental & Occupational Health" OR "Radiology, Nuclear Medicine & Medical Imaging" OR "Remote Sensing" OR "Reproductive Biology" OR "Toxicology" OR "Veterinary Sciences")	NOT TI=("review" OR "meta-analysis" OR "metaanalysis" OR "meta analysis")	125

Multiple factors	TS=(	((("multiple" OR "multi" OR "mixture" OR "mixtures" OR "combination" OR "combinations" OR "combined" OR "sequential") NEAR/10 ("change" OR "changes" OR "changing" OR "stressor" OR "stressors" OR "stress" OR "stresses" OR "driver" OR "drivers" OR "factor" OR "factors" OR "pressure" OR "pressures" OR "risk" OR "risks" OR "treatment" OR "treatments") OR "compound event" OR "compound events" O R "joint impact" OR "joint impact s" OR "multi-driver" OR "multi-drivers" OR "multidriver" OR "mu ltidrivers") AND ("anthropogenic " OR "environmental" OR "environment" OR "environments" OR "global" OR "golbally" OR "climate" OR "climatic"))	AND	((("arbuscular" OR "vesicular arbuscular" OR "VA" OR "arbutoid" OR "ericoid" OR "ericaceous" OR "monotropoid" OR "orchid" OR "orchidaceous") NEAR/1 ("mycorrhiza" OR "mycorrhizas" OR "mycorrhizal" OR "mycorrhizae" OR "mycorrhization")) OR “ectomycorrhiza” OR “ectomycorrhizas” OR “ectomycorrhizal” OR “ectomycorrhizae” OR “ectomycorrhization” OR "endomycorrhiza” OR endomycorrhizas” OR “endomycorrhizal” OR “endomycorrhizae” OR “endomycorrhization” OR “ectendomycorrhiza” OR “ectendomycorrhizas” OR “ectendomycorrhizal” OR “ectendomycorrhizae” OR “ectendomycorrhization” OR "AM fungi" OR "AM fungus" OR "AM fungal" OR "VAM fungi" OR "VAM fungus" OR "VAM fungal" OR "EcM fungi" OR EcM fungus" OR "EcM fungal" OR "ErM fungi" OR "ErM fungus" OR "ErM fungal" OR (("mycorrhiza" OR "mycorrhizas" OR "mycorrhizal" OR "mycorrhizae" OR "mycorrhization" OR "mycorrhized") AND ("inoculated" OR "inoculation" OR "fungal" OR "fungi" OR "fungus")))	)	NOT SU=("Acoustics" OR "Allergy" OR "Anesthesiology" OR "Anthropology" OR "Archaeology" OR "Architecture" OR "Area Studies" OR "Art" OR "Arts & Humanities-Other Topics" OR "Asian Studies" OR "Audiology & Speech-Language Pathology" OR "Automation & Control Systems" OR "Biomedical Social Sciences" OR "Business & Economics" OR "Cardiovascular System & Cardiology" OR "Classics" OR "Communication" OR "Computer Science" OR "Construction & Building Technology" OR "Criminology & Penology" OR "Critical Care Medicine" OR "Crystallography" OR "Cultural Studies" OR "Dance" OR "Demography" OR "Dentistry, Oral Surgery & Medicine" OR "Dermatology" OR "Development Studies" OR "Education & Educational Research" OR "Electrochemistry" OR "Emergency Medicine" OR "Ethnic Studies" OR "Family Studies" OR "Film, Radio & Television" OR "Gastroenterology & Hepatology" OR "General & Internal Medicine" OR "Geochemistry & Geophysics" OR "Geriatrics & Gerontology" OR "Government & Law" OR "Health Care Sciences & Services" OR "Hematology" OR "History" OR "History & Philosophy of Science" OR "Imaging Science & Photographic Technology" OR "Information Science & Library Science" OR "Instruments & Instrumentation" OR "Integrative & Complementary Medicine" OR "International Relations" OR "Legal Medicine" OR "Linguistics" OR "Literature" OR "Mathematical & Computational Biology" OR "Mathematical Methods In Social Sciences" OR "Mathematics" OR "Mechanics" OR "Medical Ethics" OR "Medical Informatics" OR "Medical Laboratory Technology" OR "Metallurgy & Metallurgical Engineering" OR "Microscopy" OR "Music" OR "Nuclear Science & Technology" OR "Nursing" OR "Obstetrics & Gynecology" OR "Oncology" OR "Operations Research & Management Science" OR "Ophthalmology" OR "Optics" OR "Orthopedics" OR "Otorhinolaryngology" OR "Pathology" OR "Pediatrics" OR "Philosophy" OR "Physics" OR "Polymer Science" OR "Public Administration" OR "Rehabilitation" OR "Religion" OR "Research & Experimental Medicine" OR "Respiratory System" OR "Rheumatology" OR "Robotics" OR "Social Issues" OR "Social Sciences Other Topics" OR "Social Work" OR "Sociology" OR "Sport Sciences" OR "Substance Abuse" OR "Surgery" OR "Telecommunications" OR "Theater" OR "Thermodynamics" OR "Transplantation" OR "Transportation" OR "Urology & Nephrology" OR "Women's Studies" OR "Anatomy & Morphology" OR "Astronomy & Astrophysics" OR "Behavioral Sciences" OR "Biochemistry & Molecular Biology" OR "Biophysics" OR "Biotechnology & Applied Microbiology" OR "Cell Biology" OR "Chemistry" OR "Developmental Biology" OR "Endocrinology & Metabolism" OR "Energy & Fuels" OR "Engineering" OR "Entomology" OR "Food Science & Technology" OR "Genetics & Heredity" OR "Geography" OR "Geology" OR "Immunology" OR "Infectious Diseases" OR "Materials Science" OR "Mineralogy" OR "Mining & Mineral Processing" OR "Neurosciences & Neurology" OR "Nutrition & Dietetics" OR "Paleontology" OR "Parasitology" OR "Pharmacology & Pharmacy" OR "Physical Geography" OR "Physiology" OR "Psychiatry" OR "Psychology" OR "Public, Environmental & Occupational Health" OR "Radiology, Nuclear Medicine & Medical Imaging" OR "Remote Sensing" OR "Reproductive Biology" OR "Spectroscopy" OR "Toxicology" OR "Tropical Medicine" OR "Urban Studies" OR "Veterinary Sciences" OR "Virology" OR "Water Resources")	NOT TI=("re view" OR "meta-analysis " OR "metaa nalysis" OR "meta analysis ")	211

Table S5 Eligibility criteria

Eligibility criteria for the 15 global change factors included in this study. The global change factors are: species invasion, land use change, overexploitation, biocide, elevated atmospheric carbon dioxide, drought, heavy metals, N and P deposition, elevated tropospheric ozone, sodicity, synthetic chemicals, microplastic, artificial light at night, warming and ultraviolet B radiation.

**Global change factor**	**Inclusion criteria**	**Explanation**	**Exclusion criteria**	**Factor nature**
ALAN	dark at night (control) vs artificial light at night (by installations)	light installation to manipulate illumination of system during night	no proximity to city/street lighting as treatment allowed; no light (light intensity/ colour) manipulation just throughout the day	physical
biocides	biocides applied vs biocide free control	experimentally applied	no experimental manipulation of biocide	chemical
CO2	ambient (control) vs elevated atmospheric CO2 (carbon dioxid)	FACE (free-air CO2 enrichment) units, climate-chambers, open-top chambers...	no CO2 springs (natural source, gradient approach)	chemical
drought	control vs reduction in water availability (precipitation/ natural rain/irrigation manipulation)	rainout shelter, water addition to establish a "watered" control only allowed for "greenhouse" studies (it seldomly rains inside a building)	no irrigation with saline (NaCl rich) water (for clear distinction from the GC factor sodicity)	chemical
HM	control vs contamination by zinc (Zn), copper (Cu), iron (Fe), manganese (Mn, do not get confused with magnesium Mg), cadmium (Cd), lead (Pb), chromium (chromium vi, Cr), mercury (Hg), arsenic (As);	experimental addition of heavy metal to system	no supplement studies (addition of low concentrations of nutrients to overcome malnutrition of plants); no "distance to mine/spillage/contamination" approach	chemical
invasion	introduction of an invasive alien/non-indigenous species (plant, animal, microbe...) to the test system, compared to effects of native/naturally occurring/indigenous species	experimental manipulation of the invading species or the native species	no manipulation of the invasive or native species	biological
LUC	LUC= land-use change (change in the way the land is used/converted), describes change in the biophysical attributes of a land, affects the structure and functioning of an ecosystem; we focus on *land degradation, conversion of *land to forest/grassland/cropland/wetland/urbanland; we focus on *land fragmentation/ loss of connectivity of habitats/ loss of habitats	experimental degradation of a system (from natural to altered state)	not restauration or renaturation (from degraded state back to natural), no paired-plot approach (treatment plot with control plot nearby)	biological
MP	control vs microplastic addition	experimentally applied	no nanoplastic	chemical-physical
NP deposition*	ambient (control) vs N or P enriched systems, we focus on atmospheric N and/or P deposition which can lead to eutrophication	check study context for atmospheric N or P deposition OR eutrophication; N/P applied wet or dry, simulated by application of synthetic fertilizer	we do not focus on fertilizer studies per se; no organic matter, manure, slurry, sludge treatments allowed	chemical

Overexploitation	control vs over-used/over-harvested/ over-grazed system; very often (aquatic or) forest systems	experimentally applied	no *land conversion: no land-use change	biological
ozone	ambient (control) vs elevated ozone concentration	FAOE (free-air O3 enrichment) units, climate-chambers, open-top chambers...	no natural ozone sources/ gradient approach	chemical
sodicity	ambient (control) vs elevated NaCl concentrations	experimental interventions which increase sodium concentration in system; Na+ has to be the dominant ion in the treatment	other salt ion than Na+ is the dominant ion in the treatment	chemical
SynChem	control vs product applied	experimentally applied surfactants and per-/polyfluorinated alkyl compounds	no "distance to contamination" approach	chemical
UVB	control vs increased UV-B radiation	UVB-lamps and filters	not UV-A; no radionuclides	physical
warming	ambient (control) vs elevated system temperature; we focus on warming, temperature increase and heat pulses	incubators, warming mats, heat cables, open-top chambers....	no freezing/ cooling down, no natural temperature gradient (e.g., distance to hot spring...)	physical

Table S6 Coding of general system information

Category	Coding	Explanation
setting	field vs lab	was this experiment performed in the field or under controlled environmental conditions (e.g., greenhouse); everything "outside", exposed to wind, weather, animals will be counted as "field" (e.g., pots placed outside)
system sterility	yes vs no	was the test system (e.g., soil, agar) sterile or not; in case no statement of system sterility was reported in the article we assume the system was not sterile
location	latitude and longitude data in decimal degrees	

New Phytologist

Table S7 Quality assessment

The global change factors are: species invasion, land use change, overexploitation, biocide, elevated atmospheric carbon dioxide, drought, heavy metals, N and P deposition, elevated tropospheric ozone, sodicity, synthetic chemicals, microplastic, artificial light at night, warming and ultraviolet B radiation. An article could be covered by different search strings. The search string “multi” represents no global change factor but terminologies related to studies working on multiple factors. NOF means number of factors tested in combination.

**Global change factor search string**	**hits**	invasion	luc	overexpl	biocide	co2	drought	hm	np	ozone	sodicity	synchem	mp	alan	uvb	warming	NOF	yes-match hit rate for specific tag across all GC factors [%]	yes-match hit rate for specific GC-tag for its specific GC factor [%]
Invasion	937	222	0	0	20	2	17	9	1	12	0	5	7	0	72	0	33	39.2	23.7
LUC	248	3	7	0	1	0	3	0	3	0	0	0	0	0	0	2	2	7.7	2.8
Overexploitation	135	0	1	23	1	0	0	0	0	0	0	0	0	0	0	0	1	18.5	17.0
biocides	882	22	1	6	269	2	8	8	6	2	2	0	0	0	0	2	17	37.2	30.5
CO2	751	5	1	0	5	216	36	7	39	19	13	0	0	0	0	49	68	51.9	28.8
Drought	1330	12	2	2	8	18	668	4	18	6	51	1	0	0	0	25	49	61.3	50.2
HM	1372	7	1	0	10	2	44	531	1	1	41	1	1	0	0	1	14	46.7	38.7
NP deposition	517	9	0	3	2	18	12	6	180	4	3	0	0	0	0	14	35	48.5	34.8
Ozone	97	0	0	0	3	11	2	0	3	51	1	0	0	0	4	1	15	78.4	52.6
Sodicity	730	9	0	1	5	4	48	12	2	0	372	1	0	0	0	4	26	62.7	51.0
SynChem	31	0	0	0	3	0	0	2	0	0	1	12	0	0	0	0	1	58.1	38.7
MP	2	0	0	0	0	0	0	1	0	0	0	0	1	0	0	0	1	100.0	50.0
ALAN	3	0	0	0	0	0	0	0	0	0	0	0	0	0	0	0	0	0.0	0.0
UVB	18	0	0	0	0	0	1	0	0	0	0	0	0	0	6	0	0	38.9	33.3
Warming	511	5	1	4	0	21	36	1	19	1	1	1	0	0	0	139	54	44.8	27.2
"multi"	211	5	0	5	3	10	29	1	9	2	7	0	0	0	38	18	16	60.2	7.6

Table S8 Search outcomes for search results retrieved until 2021 and since 2021

The original search represents the search conducted in the years 2021/2022 (see methods and Table S1 to S6). The "mycorrhiza" search represents the research outcomes for general mycorrhiza research using the search string "TS = ("mycorrhiza*")" in Web of Science as described in the methods.
These data were used to estimate the contribution of "global change and mycorrhizal fungi" research in the broader field of "mycorrhizal" research, for articles published till 2021 and since 2021 (updated search).

	1949-2021	2022- July 2025
"Original" search	7107	2629
"Mycorrhiza" search	29211	6638
Ratio (original-to-mycorrhiza research articles)	24.30%	39.60%

Table S9 Most common factor combinations for mycorrhiza types

The global change factors are: species invasion, land use change, overexploitation, biocide, elevated atmospheric carbon dioxide, drought, heavy metals, N and P deposition, elevated tropospheric ozone, sodicity, synthetic chemicals, microplastic, artificial light at night, warming and ultraviolet B radiation. The mycorrhiza types are: arbuscular mycorrhiza (AM), ectomycorrhiza (EcM), ericoid mycorrhiza (ErM).

**Mycorrhiza type**	**Global change factor combinations**	**Counts**	**Percent value** (within mycorrhiza type)
AM	drought,warming	26	15.9509202
AM	np,warming	15	9.20245399
AM	co2,drought	12	7.36196319
AM	drought,invasion	12	7.36196319
AM	biocide,invasion	11	6.74846626
AM	co2,warming	10	6.13496933
AM	drought,np	10	6.13496933
AM	drought,sodicity	10	6.13496933
AM	invasion,sodicity	10	6.13496933
AM	invasion,np	7	4.29447853
AM	co2,np	5	3.06748466
AM	co2,ozone	4	2.45398773
AM	invasion,warming	4	2.45398773
AM	co2,sodicity	3	1.8404908
AM	co2,hm	2	1.22699387
AM	drought,hm	2	1.22699387
AM	hm,sodicity	2	1.22699387
AM	np,ozone	2	1.22699387
AM	np,sodicity	2	1.22699387
AM	ozone,warming	2	1.22699387
AM	biocide,co2	1	0.61349693
AM	biocide,drought	1	0.61349693
AM	biocide,hm	1	0.61349693
AM	biocide,np	1	0.61349693
AM	biocide,warming	1	0.61349693
AM	co2,invasion	1	0.61349693
AM	drought,over	1	0.61349693
AM	hm,invasion	1	0.61349693

AM	hm,mp	1	0.61349693
AM	hm,warming	1	0.61349693
AM	over,warming	1	0.61349693
AM	sodicity,synchem	1	0.61349693
EcM	co2,warming	10	14.084507
EcM	drought,warming	10	14.084507
EcM	drought,np	9	12.6760563
EcM	co2,np	6	8.45070423
EcM	co2,ozone	6	8.45070423
EcM	np,warming	6	8.45070423
EcM	co2,drought	5	7.04225352
EcM	biocide,invasion	3	4.22535211
EcM	biocide,ozone	3	4.22535211
EcM	hm,np	2	2.81690141
EcM	biocide,drought	1	1.4084507
EcM	biocide,over	1	1.4084507
EcM	drought,hm	1	1.4084507
EcM	drought,invasion	1	1.4084507
EcM	drought,ozone	1	1.4084507
EcM	drought,synchem	1	1.4084507
EcM	hm,sodicity	1	1.4084507
EcM	invasion,np	1	1.4084507
EcM	invasion,warming	1	1.4084507
EcM	np,ozone	1	1.4084507
EcM	over,warming	1	1.4084507
ErM	co2,warming	3	42.8571429
ErM	co2,drought	1	14.2857143
ErM	drought,synchem	1	14.2857143
ErM	drought,warming	1	14.2857143
ErM	np,warming	1	14.2857143

New Phytologist

Table S10 Number of occurrences for mycorrhiza fungi and factor combinations

Data is given for different settings (lab vs. field) or for data excluding setting information (all). Data is given in %.

Mycorrhiza type	Setting	Single	Two-factor combination	Three-factor combination
arbuscular mycorrhiza	all	94.1	5.2	0.7
	field	90.6	7.8	1.7
	lab	95.1	4.5	0.3
ectomycorrhiza	all	90.1	9.5	0.5
	field	83	16.7	0.4
	lab	94.8	4.6	0.5
ericoid mycorrhiza	all	85.7	11.4	2.9
	field	81	19	0
	lab	93.3	0	6.7

New Phytologist

Table S11 Comparison of "till 2021" and "since 2021" searches

The global change factors are: species invasion, land use change, overexploitation, biocide, elevated atmospheric carbon dioxide, drought, heavy metals, N and P deposition, elevated tropospheric ozone, sodicity, synthetic chemicals, microplastic, artificial light at night, warming and ultraviolet B radiation.

GC search string	1949-2021	2022- July 2025
biocide	882	237
co2	751	171
temp_warming	228	135
temp_temperature	261	83
temp_heat	22	32
drought	1330	643
alan	3	0
npdepos	517	182
hm	1372	548
sodicity	730	383
invasion	937	270
ozone	97	10
luc_degradation	46	41
luc_landusechange	112	59
luc_fragmentation	90	34
uvb	18	5
synchem_surfactants	29	7
synchem_fluoro	2	2
mp	2	29
over_overexplot	10	7
over_clearcut	125	27
mutliple	211	167

Table S12 Comparison of “till 2021” and “since 2021” searches for global change factors

The global change factors are: species invasion, land use change, overexploitation, biocide, elevated atmospheric carbon dioxide, drought, heavy metals, N and P deposition, elevated tropospheric ozone, sodicity, synthetic chemicals, microplastic, artificial light at night, warming and ultraviolet B radiation.

			No. of factors tested in combination		
search string	2022- July 20225	fulfilled eligibility criteria	1	2	3
mp	29	25	23	2	0
multiple	167	59	43	15	1

Table S13 Cases of occurrence for inoculum types

Cases of occurrence for inoculum types in arbuscular mycorrhizal (AM) research. Data is given for different settings (lab vs. field) or for data excluding setting.

**Inoculum type**	**Setting**	**Counts**
AM fungal species mixture	field	73
AM fungal species mixture	lab	370
commercial multi-phylum mixture	field	6
commercial multi-phylum mixture	lab	6
single AM fungal species	field	127
single AM fungal species	lab	1184
soil community	field	351
soil community	lab	261

Table S14 Cases of occurrence for traits measured in arbuscular mycorrhizal fungi

Cases of occurrence for traits measured in arbuscular mycorrhizal (AM) research for different inoculum types. Data is given for different settings (lab vs. field) or for data excluding setting.

**Inoculum type**	**Setting**	**Intraradicle AM fungal structures**	**Extraradicle AM fungal structures**	**Spore abundance**	**Spore germination capability**	**Fungal community metrics**
single AM fungal species	all	1097	108	94	18	8
single AM fungal species	field	86	2	6		2
single AM fungal species	lab	1015	107	88	18	6
AM fungal species mixture	all	366	18	40	1	18
AM fungal species mixture	field	49	1	7		5
AM fungal species mixture	lab	323	17	34	1	13
soil community	all	396	68	87	1	119
soil community	field	184	45	62		94
soil community	lab	224	24	28	1	27

Table S15 Cases of occurrence for global change factors and factor combinations

Cases of occurrence for global change factors and factor combinations in single species studies in arbuscular mycorrhizal (AM) research

Parameter	Level	Counts	Percent value
biological factors	species invasion	53	3.9
	land use change	2	0.1
	overexploitation	0	0.0
chemical factors	biocides	102	7.5
	elevated atmospheric carbon dioxide	50	3.7
	drought	466	34.4
	heavy metals	344	25.4
	N and P deposition	13	1.0
	elevated tropospheric ozone	6	0.4
	sodicity	278	20.5
	synthetic chemicals	5	0.4
chemical/physical factor	microplastic	0	0.0
physical factor	ultraviolet B radiation	0	0.0
	warming	36	2.7
number of factors combined	1	1271	97.3
	2	34	2.6
	3	1*	0.1

* one study reporting data for 2 AM fungal species

New Phytologist

Notes S1 Reference list of database articles

List of dois for articles fulfilling the eligibility criteria.

10.1002/agg2.20164
10.1002/agj2.20116
10.1002/ajb2.1731
10.1002/clen.200900252
10.1002/clen.201700665
10.1002/eap.1613
10.1002/eap.1763
10.1002/eap.2444
10.1002/ece3.1145
10.1002/ece3.1952
10.1002/ece3.24
10.1002/ece3.2900
10.1002/ece3.3226
10.1002/ece3.3578
10.1002/ece3.3635
10.1002/ece3.4112
10.1002/ece3.4397
10.1002/ece3.5407
10.1002/ece3.6703
10.1002/ece3.750
10.1002/ecm.1453
10.1002/ecs2.1527
10.1002/ecs2.2640
10.1002/ecs2.2775
10.1002/ecs2.3228
10.1002/ecs2.3460
10.1002/ecy.1595
10.1002/ecy.1864
10.1002/ecy.2357
10.1002/ecy.2401
10.1002/ecy.2740
10.1002/ecy.2850
10.1002/ecy.2855
10.1002/ecy.3201
10.1002/ecy.3328
10.1002/ecy.3330
10.1002/etc.2661
10.1002/etc.5620170728
10.1002/etc.5620171021
10.1002/etc.5620190322
10.1002/fes3.314
10.1002/fsn3.1884
10.1002/ieam.4350
10.1002/jeq2.20212
10.1002/jobm.200710320
10.1002/jobm.201900294
10.1002/jpln.19881510402
10.1002/jpln.19911540502
10.1002/jpln.201000051
10.1002/jpln.201100349
10.1002/jpln.201200079
10.1002/jpln.201200618
10.1002/jpln.201300548
10.1002/jpln.201400092
10.1002/jpln.201700575
10.1002/jpln.201800160
10.1002/jpln.201800262
10.1002/jpln.202000335
10.1002/jsfa.10166
10.1002/jsfa.11370
10.1002/jsfa.11530
10.1002/jsfa.6021
10.1002/jsfa.8201
10.1002/ldr.2784
10.1002/ldr.2842
10.1002/ldr.3491
10.1002/ldr.3651
10.1002/ldr.3773
10.1002/ldr.4011
10.1002/ldr.4153
10.1002/ldr.4509
10.1002/pld3.350
10.1002/pmic.200800336
10.1002/pmic.200900436
10.1002/ppp3.10094
10.1002/ppp3.10172
10.1002/ppp3.10174
10.1002/ppp3.10185
10.1002/ppp3.10193
10.1002/ppp3.10222
10.1002/ps.5140
10.1002/saj2.20201
10.1002/saj2.20218
10.1002/saj2.20334
10.1006/anbo.1997.0526
10.1007/BF00007874
10.1007/BF00007944
10.1007/BF00007946
10.1007/BF00007960
10.1007/BF00009380
10.1007/BF00009543
10.1007/BF00009964
10.1007/BF00009965
10.1007/BF00009970
10.1007/BF00010352
10.1007/BF00010449
10.1007/BF00010915
10.1007/BF00010925
10.1007/BF00011011
10.1007/BF00011359
10.1007/BF00011589
10.1007/BF00012041
10.1007/BF00012064
10.1007/BF00015307
10.1007/BF00016612
10.1007/BF00150343
10.1007/BF00202342
10.1007/BF00203335
10.1007/BF00203769
10.1007/BF00210694
10.1007/BF00210696
10.1007/BF00292569
10.1007/BF00317079
10.1007/BF00336445
10.1007/BF00337215
10.1007/BF00346056
10.1007/BF00979666
10.1007/BF01273247
10.1007/BF01420215
10.1007/BF01701623
10.1007/BF01875449
10.1007/BF02181749
10.1007/BF02181854
10.1007/BF02203086
10.1007/BF02220192
10.1007/BF02370276
10.1007/BF02370548
10.1007/BF02390226
10.1007/BF02465228
10.1007/BF02803141
10.1007/BF02803148
10.1007/BF02803152
10.1007/BF02804004
10.1007/BF03182887
10.1007/BF03326287
10.1007/PL00009721
10.1007/PL00009757
10.1007/PL00021509
10.1007/s00128-021-03113-x
10.1007/s00128-021-03240-5
10.1007/s00128-021-03329-x
10.1007/s00248-004-0044-4
10.1007/s00248-006-9004-5
10.1007/s00248-006-9015-2
10.1007/s00248-007-9239-9
10.1007/s00248-007-9249-7
10.1007/s00248-007-9269-3
10.1007/s00248-008-9390-y
10.1007/s00248-008-9437-0
10.1007/s00248-009-9544-6
10.1007/s00248-011-9837-4
10.1007/s00248-011-9972-y
10.1007/s00248-014-0383-8
10.1007/s00248-014-0443-0
10.1007/s00248-016-0875-9
10.1007/s00248-017-0999-6
10.1007/s00248-017-1044-5
10.1007/s00248-020-01637-z
10.1007/s00248-021-01721-y
10.1007/s00248-021-01744-5
10.1007/s00248-021-01779-8
10.1007/s00248-021-01814-8
10.1007/s00248-021-01815-7
10.1007/s00248-021-01841-5
10.1007/s00248-021-01853-1
10.1007/s00248-021-01886-6
10.1007/s00248-021-01895-5
10.1007/s00248-021-01931-4
10.1007/s00248-021-01937-y
10.1007/s00267-004-5006-6
10.1007/s00284-020-02034-y
10.1007/s00284-021-02432-w
10.1007/s00294-010-0298-y
10.1007/s00344-007-9038-z
10.1007/s00344-009-9136-1
10.1007/s00344-010-9191-7
10.1007/s00344-011-9211-2
10.1007/s00344-011-9239-3
10.1007/s00344-011-9252-6
10.1007/s00344-012-9265-9
10.1007/s00344-013-9346-4
10.1007/s00344-013-9347-3
10.1007/s00344-013-9410-0
10.1007/s00344-014-9414-4
10.1007/s00344-015-9494-9
10.1007/s00344-016-9607-0
10.1007/s00344-016-9659-1
10.1007/s00344-017-9675-9
10.1007/s00344-017-9690-x
10.1007/s00344-017-9708-4
10.1007/s00344-018-9815-x
10.1007/s00344-020-10165-6
10.1007/s00344-020-10174-5
10.1007/s00344-020-10197-y
10.1007/s00344-021-10489-x
10.1007/s00344-021-10529-6
10.1007/s00374-003-0580-y
10.1007/s00374-003-0621-6
10.1007/s00374-003-0632-3
10.1007/s00374-003-0636-z
10.1007/s003740050212
10.1007/s003740050298
10.1007/s003740050405
10.1007/s003740050590
10.1007/s00374-005-0845-8
10.1007/s00374-007-0232-8
10.1007/s00374-011-0563-3
10.1007/s00374-011-0590-0
10.1007/s00374-011-0621-x
10.1007/s00374-012-0732-z
10.1007/s00374-012-0751-9
10.1007/s00374-012-0770-6
10.1007/s00374-013-0827-1
10.1007/s00374-014-0942-7
10.1007/s00374-015-1013-4
10.1007/s00374-016-1165-x
10.1007/s00374-017-1181-5
10.1007/s00374-017-1238-5
10.1007/s00374-017-1251-8

10.1007/s00374-018-1283-8
10.1007/s00374-018-1328-z
10.1007/s00374-019-01362-x
10.1007/s00374-019-01425-z
10.1007/s00374-021-01593-x
10.1007/s003749900180
10.1007/s00425-012-1759-y
10.1007/s00425-017-2808-3
10.1007/s00425-021-03569-5
10.1007/s004420000385
10.1007/s00442-002-0932-6
10.1007/s00442-003-1441-y
10.1007/s004420050096
10.1007/s004420050375
10.1007/s004420050376
10.1007/s004420050685
10.1007/s004420050821
10.1007/s00442-010-1668-3
10.1007/s00442-010-1736-8
10.1007/s00442-012-2258-3
10.1007/s00442-012-2562-y
10.1007/s00442-013-2705-9
10.1007/s00442-014-2940-8
10.1007/s00442-015-3337-z
10.1007/s00442-015-3470-8
10.1007/s00442-018-4081-y
10.1007/s00442-019-04419-8
10.1007/s00442-020-04797-4
10.1007/s00442-021-04986-9
10.1007/s00442-021-05065-9
10.1007/s00442-021-05079-3
10.1007/s00468-004-0361-8
10.1007/s004680050161
10.1007/s00468-008-0243-6
10.1007/s00468-009-0379-z
10.1007/s00468-011-0613-3
10.1007/s00468-013-0939-0
10.1007/s00468-015-1253-9
10.1007/s00468-017-1613-8
10.1007/s00468-020-02015-5
10.1007/s00468-021-02234-4
10.1007/s005720000055
10.1007/s005720000066
10.1007/s005720000067
10.1007/s005720000071
10.1007/s005720000075
10.1007/s005720000084
10.1007/s00572-001-0144-7
10.1007/s00572-002-0161-1
10.1007/s00572-002-0170-0
10.1007/s00572-002-0197-2
10.1007/s00572-002-0211-8
10.1007/s00572-003-0223-z
10.1007/s00572-003-0228-7
10.1007/s00572-003-0235-8
10.1007/s00572-003-0247-4
10.1007/s00572-003-0261-6
10.1007/s00572-003-0265-2
10.1007/s00572-003-0269-y
10.1007/s00572-003-0274-1
10.1007/s00572-003-0276-z
10.1007/s00572-003-0281-2
10.1007/s00572-003-0289-7
10.1007/s00572-004-0309-2
10.1007/s00572-004-0320-7
10.1007/s00572-004-0325-2
10.1007/s00572-004-0336-z
10.1007/s00572-004-0345-y
10.1007/s00572-005-0001-1
10.1007/s00572-005-0009-6
10.1007/s00572-005-0016-7
10.1007/s00572-005-0020-y
10.1007/s00572-005-0024-7
10.1007/s005720050068
10.1007/s005720050072
10.1007/s005720050113
10.1007/s005720050116
10.1007/s005720050119
10.1007/s005720050159
10.1007/s005720050160
10.1007/s005720050166
10.1007/s005720050209
10.1007/s005720050237
10.1007/s005720050277
10.1007/s005720050289
10.1007/s00572-005-0346-5
10.1007/s00572-006-0043-z
10.1007/s00572-006-0045-x
10.1007/s00572-006-0046-9
10.1007/s00572-006-0072-7
10.1007/s00572-006-0073-6
10.1007/s00572-006-0075-4
10.1007/s00572-006-0082-5
10.1007/s00572-006-0084-3
10.1007/s00572-007-0108-7
10.1007/s00572-007-0134-5
10.1007/s00572-008-0162-9
10.1007/s00572-008-0174-5
10.1007/s00572-008-0177-2
10.1007/s00572-008-0180-7
10.1007/s00572-008-0184-3
10.1007/s00572-008-0203-4
10.1007/s00572-009-0230-9
10.1007/s00572-009-0238-1
10.1007/s00572-009-0250-5
10.1007/s00572-009-0259-9
10.1007/s00572-009-0267-9
10.1007/s00572-009-0285-7
10.1007/s00572-009-0294-6
10.1007/s00572-009-0296-4
10.1007/s005720100098
10.1007/s005720100114
10.1007/s005720100118
10.1007/s00572-010-0310-x
10.1007/s00572-010-0311-9
10.1007/s00572-010-0316-4
10.1007/s00572-010-0329-z
10.1007/s00572-010-0331-5
10.1007/s00572-010-0334-2
10.1007/s00572-010-0343-1
10.1007/s00572-010-0344-0
10.1007/s00572-010-0350-2
10.1007/s00572-010-0353-z
10.1007/s00572-011-0370-6
10.1007/s00572-011-0385-z
10.1007/s00572-011-0392-0
10.1007/s00572-011-0407-x
10.1007/s00572-011-0408-9
10.1007/s00572-012-0430-6
10.1007/s00572-012-0433-3
10.1007/s00572-012-0443-1
10.1007/s00572-012-0449-8
10.1007/s00572-012-0460-0
10.1007/s00572-013-0477-z
10.1007/s00572-013-0517-8
10.1007/s00572-013-0529-4
10.1007/s00572-013-0545-4
10.1007/s00572-013-0546-3
10.1007/s00572-014-0578-3
10.1007/s00572-014-0595-2
10.1007/s00572-014-0596-1
10.1007/s00572-014-0598-z
10.1007/s00572-014-0600-9
10.1007/s00572-014-0603-6
10.1007/s00572-014-0608-1
10.1007/s00572-014-0610-7
10.1007/s00572-015-0629-4
10.1007/s00572-015-0632-9
10.1007/s00572-015-0638-3
10.1007/s00572-015-0650-7
10.1007/s00572-015-0653-4
10.1007/s00572-015-0654-3
10.1007/s00572-015-0670-3
10.1007/s00572-016-0686-3
10.1007/s00572-016-0693-4
10.1007/s00572-016-0700-9
10.1007/s00572-016-0704-5
10.1007/s00572-016-0723-2
10.1007/s00572-016-0729-9
10.1007/s00572-016-0749-5
10.1007/s00572-017-0765-0
10.1007/s00572-017-0767-y
10.1007/s00572-017-0775-y
10.1007/s00572-017-0778-8
10.1007/s00572-017-0794-8
10.1007/s00572-018-0819-y
10.1007/s00572-018-0823-2
10.1007/s00572-018-0827-y
10.1007/s00572-018-0853-9
10.1007/s00572-018-0866-4
10.1007/s00572-018-0873-5
10.1007/s00572-018-0875-3
10.1007/s00572-019-00880-8
10.1007/s00572-019-00893-3
10.1007/s00572-019-00900-7
10.1007/s00572-019-00902-5
10.1007/s00572-020-00935-1
10.1007/s00572-020-00942-2
10.1007/s00572-020-00949-9

10.1007/s00572-020-00952-0
10.1007/s00572-020-00953-z
10.1007/s00572-020-00954-y
10.1007/s00572-020-00957-9
10.1007/s00572-020-00958-8
10.1007/s00572-020-00962-y
10.1007/s00572-020-00963-x
10.1007/s00572-020-00990-8
10.1007/s00572-020-00992-6
10.1007/s00572-020-01012-3
10.1007/s00572-021-01021-w
10.1007/s00572-021-01025-6
10.1007/s00572-021-01033-6
10.1007/s00572-021-01047-0
10.1007/s00572-021-01052-3
10.1007/s00572-021-01056-z
10.1007/s00709-015-0892-4
10.1007/s10021-003-0182-4
10.1007/s10021-010-9347-0
10.1007/s10021-010-9360-3
10.1007/s10021-018-0280-y
10.1007/s10021-020-00575-8
10.1007/s10021-021-00624-w
10.1007/s10021-021-00689-7
10.1007/s10021-021-00715-8
10.1007/s10265-021-01275-7
10.1007/s10267-010-0069-0
10.1007/s10310-007-0040-x
10.1007/s10342-010-0435-7
10.1007/s10342-013-0751-9
10.1007/s10342-021-01383-y
10.1007/s10343-017-0393-9
10.1007/s10343-017-0394-8
10.1007/s10343-019-00461-x
10.1007/s10343-019-00480-8
10.1007/s10343-020-00533-3
10.1007/s10343-021-00588-w
10.1007/s10457-005-2477-2
10.1007/s10457-008-9197-3
10.1007/s10457-017-0146-x
10.1007/s10457-020-00488-4
10.1007/s10482-021-01612-9
10.1007/s10530-006-0003-8
10.1007/s10530-009-9435-2
10.1007/s10530-010-9920-7
10.1007/s10530-012-0238-5
10.1007/s10530-012-0371-1
10.1007/s10530-013-0464-5
10.1007/s10530-013-0469-0
10.1007/s10530-013-0584-y
10.1007/s10530-014-0642-0
10.1007/s10530-016-1166-6
10.1007/s10530-018-1804-2
10.1007/s10530-019-01950-w
10.1007/s10530-019-02031-8
10.1007/s10530-020-02268-8
10.1007/s10530-021-02649-7
10.1007/s10531-021-02287-4
10.1007/s10533-015-0110-7
10.1007/s10533-015-0159-3
10.1007/s10533-021-00774-y
10.1007/s10533-021-00871-y
10.1007/s10534-013-9634-2
10.1007/s10534-018-00164-2
10.1007/s10534-021-00340-x
10.1007/s10646-007-0149-x
10.1007/s10646-014-1331-6
10.1007/s10646-014-1368-6
10.1007/s10646-016-1678-y
10.1007/s10646-018-1946-0
10.1007/s10646-019-02042-0
10.1007/s10646-020-02298-x
10.1007/s10646-021-02492-5
10.1007/s10653-005-9020-2
10.1007/s10653-020-00627-x
10.1007/s10658-018-01641-7
10.1007/s10658-020-02150-2
10.1007/s10661-006-9414-3
10.1007/s10661-015-4557-8
10.1007/s10661-016-5428-7
10.1007/s10725-011-9624-8
10.1007/s10725-012-9771-6
10.1007/s10725-014-0016-8
10.1007/s10725-014-9977-x
10.1007/s10725-015-0099-x
10.1007/s10725-016-0146-2
10.1007/s10725-018-0414-4
10.1007/s10886-009-9644-2
10.1007/s10886-013-0241-z
10.1007/s11033-019-04974-6
10.1007/s11056-010-9198-8
10.1007/s11056-011-9249-9
10.1007/s11056-012-9309-9
10.1007/s11056-012-9349-1
10.1007/s11056-014-9417-9
10.1007/s11056-014-9442-8
10.1007/s11056-014-9455-3
10.1007/s11056-018-9681-1
10.1007/s11056-021-09875-w
10.1007/s11056-021-09879-6
10.1007/s11099-005-0037-7
10.1007/s11099-011-0064-5
10.1007/s11099-012-0024-8
10.1007/s11099-012-0035-5
10.1007/s11099-015-0100-y
10.1007/s11099-016-0650-7
10.1007/s11099-017-0662-y
10.1007/s11103-005-4210-y
10.1007/s11103-009-9492-z
10.1007/s11104-004-1559-7
10.1007/s11104-004-2267-z
10.1007/s11104-004-3847-7
10.1007/s11104-005-0415-8
10.1007/s11104-005-0606-3
10.1007/s11104-005-1238-3
10.1007/s11104-005-3701-6
10.1007/s11104-005-5349-7
10.1007/s11104-006-9001-y
10.1007/s11104-006-9015-5
10.1007/s11104-006-9034-2
10.1007/s11104-006-9091-6
10.1007/s11104-006-9106-3
10.1007/s11104-006-9183-3
10.1007/s11104-007-9203-y
10.1007/s11104-007-9230-8
10.1007/s11104-007-9290-9
10.1007/s11104-007-9306-5
10.1007/s11104-007-9392-4
10.1007/s11104-007-9509-9
10.1007/s11104-007-9526-8
10.1007/s11104-008-9619-z
10.1007/s11104-008-9677-2
10.1007/s11104-008-9678-1
10.1007/s11104-008-9706-1
10.1007/s11104-009-0158-z
10.1007/s11104-009-0255-z
10.1007/s11104-009-9972-6
10.1007/s11104-010-0317-2
10.1007/s11104-010-0591-z
10.1007/s11104-010-0709-3
10.1007/s11104-011-0727-9
10.1007/s11104-011-0805-z
10.1007/s11104-011-0809-8
10.1007/s11104-011-0883-y
10.1007/s11104-011-1005-6
10.1007/s11104-011-1098-y
10.1007/s11104-012-1190-y
10.1007/s11104-012-1312-6
10.1007/s11104-012-1409-y
10.1007/s11104-012-1431-0
10.1007/s11104-013-1601-8
10.1007/s11104-013-1610-7
10.1007/s11104-013-1635-y
10.1007/s11104-013-1670-8
10.1007/s11104-013-1950-3
10.1007/s11104-014-2026-8
10.1007/s11104-014-2091-z
10.1007/s11104-014-2158-x
10.1007/s11104-014-2203-9
10.1007/s11104-014-2247-x
10.1007/s11104-014-2374-4
10.1007/s11104-015-2420-x
10.1007/s11104-015-2451-3
10.1007/s11104-015-2520-7
10.1007/s11104-015-2599-x

10.1007/s11104-015-2635-x
10.1007/s11104-015-2656-5
10.1007/s11104-015-2776-y
10.1007/s11104-016-2942-x
10.1007/s11104-016-3018-7
10.1007/s11104-016-3062-3
10.1007/s11104-017-3283-0
10.1007/s11104-017-3308-8
10.1007/s11104-017-3354-2
10.1007/s11104-017-3367-x
10.1007/s11104-017-3403-x
10.1007/s11104-017-3409-4
10.1007/s11104-017-3414-7
10.1007/s11104-017-3444-1
10.1007/s11104-017-3532-2
10.1007/s11104-018-03900-2
10.1007/s11104-018-03903-z
10.1007/s11104-018-03917-7
10.1007/s11104-018-3823-2
10.1007/s11104-019-04250-3
10.1007/s11104-019-04258-9
10.1007/s11104-019-04297-2
10.1007/s11104-019-04355-9
10.1007/s11104-019-04365-7
10.1007/s11104-020-04537-w
10.1007/s11104-020-04594-1
10.1007/s11104-020-04609-x
10.1007/s11104-020-04618-w
10.1007/s11104-020-04620-2
10.1007/s11104-020-04678-y
10.1007/s11104-020-04746-3
10.1007/s11104-020-04805-9
10.1007/s11104-021-04831-1
10.1007/s11104-021-04949-2
10.1007/s11104-021-04972-3
10.1007/s11104-021-04979-w
10.1007/s11104-021-05020-w
10.1007/s11104-021-05073-x
10.1007/s11104-021-05085-7
10.1007/s11104-021-05092-8
10.1007/s11104-021-05193-4
10.1007/s11104-022-05454-w
10.1007/s11120-018-0538-4
10.1007/s11258-007-9350-5
10.1007/s11258-008-9554-3
10.1007/s11258-011-9940-0
10.1007/s11258-012-0079-4
10.1007/s11258-013-0179-9
10.1007/s11258-014-0410-3
10.1007/s11258-015-0461-0
10.1007/s11258-016-0675-9
10.1007/s11258-018-0803-9
10.1007/s11258-018-0816-4
10.1007/s11258-018-0832-4
10.1007/s11258-018-0857-8
10.1007/s11258-018-0900-9
10.1007/s11258-020-01014-3
10.1007/s11258-020-01042-z
10.1007/s11258-021-01114-8
10.1007/s11258-021-01135-3
10.1007/s11258-021-01156-y
10.1007/s11270-005-7711-z
10.1007/s11270-006-9171-5
10.1007/s11270-009-0131-8
10.1007/s11270-011-0780-2
10.1007/s11270-011-0902-x
10.1007/s11270-014-1930-0
10.1007/s11270-016-3183-6
10.1007/s11270-016-3214-3
10.1007/s11270-019-4295-6
10.1007/s11270-019-4381-9
10.1007/s11270-021-05061-y
10.1007/s11270-022-05561-5
10.1007/s11274-004-3572-7
10.1007/s11274-006-9337-8
10.1007/s11274-007-9368-9
10.1007/s11274-008-9681-y
10.1007/s11274-019-2656-3
10.1007/s11284-006-0161-2
10.1007/s11284-006-0176-8
10.1007/s11284-015-1328-5
10.1007/s11355-009-0072-9
10.1007/s11355-021-00455-w
10.1007/s11356-012-1056-0
10.1007/s11356-012-1440-9
10.1007/s11356-013-2072-4
10.1007/s11356-013-2098-7
10.1007/s11356-013-2154-3
10.1007/s11356-014-3209-9
10.1007/s11356-015-5502-7
10.1007/s11356-015-5706-x
10.1007/s11356-016-6337-6
10.1007/s11356-016-7283-z
10.1007/s11356-016-7984-3
10.1007/s11356-016-8287-4
10.1007/s11356-017-1157-x
10.1007/s11356-017-9463-x
10.1007/s11356-017-9633-x
10.1007/s11356-018-2422-3
10.1007/s11356-018-2452-x
10.1007/s11356-018-3569-7
10.1007/s11356-018-3923-9
10.1007/s11356-019-04256-5
10.1007/s11356-019-05201-2
10.1007/s11356-019-05323-7
10.1007/s11356-019-06237-0
10.1007/s11356-019-06785-5
10.1007/s11356-020-11215-y
10.1007/s11356-021-13205-0
10.1007/s11356-021-13755-3
10.1007/s11356-021-14500-6
10.1007/s11356-021-14716-6
10.1007/s11356-021-14740-6
10.1007/s11356-021-14777-7
10.1007/s11356-021-14821-6
10.1007/s11356-021-15045-4
10.1007/s11356-021-15326-y
10.1007/s11356-021-15367-3
10.1007/s11356-021-15382-4
10.1007/s11356-021-15520-y
10.1007/s11356-021-16169-3
10.1007/s11356-021-16776-0
10.1007/s11356-021-17256-1
10.1007/s11356-021-17353-1
10.1007/s11368-009-0119-4
10.1007/s11368-010-0259-6
10.1007/s11368-020-02849-z
10.1007/s11368-021-02892-4
10.1007/s11368-021-03115-6
10.1007/s11434-013-5961-5
10.1007/s11434-014-0602-1
10.1007/s11557-010-0721-2
10.1007/s11557-015-1108-1
10.1007/s11557-021-01699-4
10.1007/s11557-021-01721-9
10.1007/s11627-012-9485-5
10.1007/s11676-017-0583-4
10.1007/s11676-019-01007-7
10.1007/s11676-019-01053-1
10.1007/s11676-019-01056-y
10.1007/s11676-020-01190-y
10.1007/s11738-002-0031-7
10.1007/s11738-004-0007-x
10.1007/s11738-004-0008-9
10.1007/s11738-007-0065-y
10.1007/s11738-008-0260-5
10.1007/s11738-009-0407-z
10.1007/s11738-010-0670-z
10.1007/s11738-011-0849-y
10.1007/s11738-012-0933-y
10.1007/s11738-012-0966-2
10.1007/s11738-012-1109-5
10.1007/s11738-013-1295-9
10.1007/s11738-014-1546-4
10.1007/s11738-014-1636-3
10.1007/s11738-015-1932-6
10.1007/s11738-015-2054-x
10.1007/s11738-016-2207-6
10.1007/s11738-017-2388-7
10.1007/s11738-017-2493-7
10.1007/s11738-018-2656-1
10.1007/s11738-018-2692-x
10.1007/s11738-019-2842-9
10.1007/s11738-020-03191-0
10.1007/s11738-021-03250-0

10.1007/s11756-021-00700-5
10.1007/s11756-021-00759-0
10.1007/s11756-021-00919-2
10.1007/s12011-011-9267-7
10.1007/s12088-018-00776-9
10.1007/s12223-019-00696-1
10.1007/s12237-009-9254-9
10.1007/s12275-013-2423-3
10.1007/s12275-021-0317-3
10.1007/s12298-016-0382-y
10.1007/s12298-017-0439-6
10.1007/s12298-019-00658-4
10.1007/s12298-019-00727-8
10.1007/s12298-021-01043-w
10.1007/s12355-018-0640-0
10.1007/s12600-013-0356-3
10.1007/s13199-010-0058-2
10.1007/s13199-010-0080-4
10.1007/s13199-010-0097-8
10.1007/s13199-012-0152-8
10.1007/s13199-012-0191-1
10.1007/s13199-012-0199-6
10.1007/s13199-013-0260-0
10.1007/s13199-015-0318-2
10.1007/s13199-016-0466-z
10.1007/s13199-017-0497-0
10.1007/s13199-018-0573-0
10.1007/s13199-019-00621-7
10.1007/s13199-019-00633-3
10.1007/s13199-019-00645-z
10.1007/s13199-021-00761-9
10.1007/s13199-021-00765-5
10.1007/s13199-021-00770-8
10.1007/s13199-021-00783-3
10.1007/s13199-021-00792-2
10.1007/s13199-021-00794-0
10.1007/s13199-021-00813-0
10.1007/s13205-012-0046-8
10.1007/s13205-020-02559-w
10.1007/s13213-013-0706-x
10.1007/s13213-014-0895-y
10.1007/s13225-020-00453-7
10.1007/s13313-020-00685-w
10.1007/s13562-021-00745-2
10.1007/s13580-017-0122-4
10.1007/s13580-020-00273-3
10.1007/s13593-012-0083-z
10.1007/s13593-020-00659-8
10.1007/s13595-013-0329-1
10.1007/s13595-018-0714-x
10.1007/s13595-018-0747-1
10.1007/s13762-020-03000-8
10.1007/s13762-021-03783-4
10.1007/s13765-015-0072-4
10.1007/s40333-014-0046-0
10.1007/s40415-018-0457-9
10.1007/s40626-014-0019-x
10.1007/s40626-016-0081-7
10.1007/s40626-017-0089-7
10.1007/S40995-018-0543-7
10.1007/s40995-021-01073-9
10.1007/s42729-019-00032-z
10.1007/s42729-019-00075-2
10.1007/s42729-019-00146-4
10.1007/s42729-020-00193-2
10.1007/s42729-021-00493-1
10.1007/s42729-021-00523-y
10.1007/s42729-021-00565-2
10.1007/s42729-021-00590-1
10.1007/s42729-021-00596-9
10.1007/s42729-021-00666-y
10.1007/s42729-021-00684-w
10.1007/s42729-021-00690-y
10.1007/s42770-019-00125-y
10.1007/s42770-021-00486-3
10.1007/s42773-021-00114-1
10.1007/s42965-021-00152-7
10.1007/s42977-020-00037-1
10.1007/s42977-020-00054-0
10.1016/0038-0717(87)90131-3
10.1016/0038-0717(93)90009-Z
10.1016/0038-0717(94)00179-5
10.1016/0038-0717(94)90088-4
10.1016/0048-9697(94)90169-4
10.1016/0098-8472(91)90055-S
10.1016/0098-8472(93)90045-H
10.1016/0143-1471(83)90050-8
10.1016/0143-1471(83)90051-X
10.1016/0143-1471(85)90100-X
10.1016/0167-8809(90)90073-M
10.1016/0167-8809(90)90094-T
10.1016/0167-8809(90)90291-K
10.1016/0167-8809(91)90075-9
10.1016/0167-8809(91)90096-G
10.1016/0167-8809(93)90056-U
10.1016/0167-8809(93)90111-2
10.1016/0167-8809(94)90110-4
10.1016/0168-1923(94)90051-5
10.1016/0168-9452(95)04184-V
10.1016/0269-7491(88)90107-8
10.1016/0269-7491(90)90041-A
10.1016/0269-7491(90)90049-I
10.1016/0269-7491(90)90092-Q
10.1016/0269-7491(91)90004-G
10.1016/0269-7491(92)90118-T
10.1016/0269-7491(94)90188-0
10.1016/0269-7491(94)90189-9
10.1016/0269-7491(95)93444-5
10.1016/0304-4238(93)90106-Z
10.1016/0378-1127(93)90171-I
10.1016/0378-1127(94)06088-Z
10.1016/0378-1127(95)03567-5
10.1016/0929-1393(96)00092-3
10.1016/i.jprot.2017.03.024
10.1016/j.actao.2012.09.002
10.1016/j.actao.2014.10.008
10.1016/j.actao.2015.06.004
10.1016/j.actao.2016.12.005
10.1016/j.actao.2017.04.003
10.1016/j.actao.2018.07.003
10.1016/j.agee.2008.08.010
10.1016/j.agee.2014.12.017
10.1016/j.agee.2015.07.026
10.1016/j.agee.2016.05.037
10.1016/j.agee.2016.06.011
10.1016/j.agee.2017.05.014
10.1016/j.agee.2017.10.026
10.1016/j.agee.2018.02.016
10.1016/j.agee.2019.106712
10.1016/j.agee.2021.107369
10.1016/j.agee.2021.107503
10.1016/j.agee.2021.107521
10.1016/j.agrformet.2015.06.017
10.1016/j.agrformet.2017.07.025
10.1016/j.agrformet.2019.107619
10.1016/j.agrformet.2020.108144
10.1016/j.agsy.2020.103008
10.1016/j.agwat.2012.11.007
10.1016/j.agwat.2017.12.004
10.1016/j.agwat.2017.12.012
10.1016/j.agwat.2018.03.020
10.1016/j.agwat.2019.04.022
10.1016/j.agwat.2019.105768
10.1016/j.agwat.2020.106234
10.1016/j.agwat.2020.106416
10.1016/j.agwat.2020.106635
10.1016/j.apsoil.2003.10.010
10.1016/j.apsoil.2003.11.002
10.1016/j.apsoil.2004.12.006
10.1016/j.apsoil.2005.01.007
10.1016/j.apsoil.2005.02.017
10.1016/j.apsoil.2006.04.004
10.1016/j.apsoil.2007.03.002
10.1016/j.apsoil.2008.02.007
10.1016/j.apsoil.2008.06.002
10.1016/j.apsoil.2008.10.002
10.1016/j.apsoil.2008.10.007
10.1016/j.apsoil.2009.03.004
10.1016/j.apsoil.2009.03.007
10.1016/j.apsoil.2009.07.004
10.1016/j.apsoil.2009.12.004
10.1016/j.apsoil.2010.05.001
10.1016/j.apsoil.2011.02.005
10.1016/j.apsoil.2011.04.005
10.1016/j.apsoil.2011.04.014

10.1016/j.apsoil.2011.10.018
10.1016/j.apsoil.2012.03.003
10.1016/j.apsoil.2012.09.013
10.1016/j.apsoil.2013.01.007
10.1016/j.apsoil.2013.05.012
10.1016/j.apsoil.2013.05.013
10.1016/j.apsoil.2013.06.001
10.1016/j.apsoil.2013.06.011
10.1016/j.apsoil.2013.10.006
10.1016/j.apsoil.2013.12.005
10.1016/j.apsoil.2014.04.001
10.1016/j.apsoil.2014.11.016
10.1016/j.apsoil.2015.01.006
10.1016/j.apsoil.2015.05.004
10.1016/j.apsoil.2015.07.014
10.1016/j.apsoil.2015.10.003
10.1016/j.apsoil.2015.11.001
10.1016/j.apsoil.2015.11.004
10.1016/j.apsoil.2016.01.003
10.1016/j.apsoil.2016.03.015
10.1016/j.apsoil.2016.06.005
10.1016/j.apsoil.2017.06.041
10.1016/j.apsoil.2017.07.030
10.1016/j.apsoil.2017.09.010
10.1016/j.apsoil.2017.09.019
10.1016/j.apsoil.2017.12.003
10.1016/j.apsoil.2018.01.012
10.1016/j.apsoil.2019.04.020
10.1016/j.apsoil.2019.06.013
10.1016/j.apsoil.2019.06.014
10.1016/j.apsoil.2019.08.006
10.1016/j.apsoil.2019.103370
10.1016/j.apsoil.2019.103375
10.1016/j.apsoil.2019.103394
10.1016/j.apsoil.2019.103443
10.1016/j.apsoil.2020.103511
10.1016/j.apsoil.2020.103590
10.1016/j.apsoil.2020.103722
10.1016/j.apsoil.2020.103736
10.1016/j.apsoil.2020.103763
10.1016/j.apsoil.2020.103810
10.1016/j.apsoil.2020.103814
10.1016/j.apsoil.2021.103896
10.1016/j.apsoil.2021.103927
10.1016/j.apsoil.2021.103991
10.1016/j.apsoil.2021.103998
10.1016/j.apsoil.2021.104030
10.1016/j.apsoil.2021.104039
10.1016/j.apsoil.2021.104042
10.1016/j.apsoil.2021.104054
10.1016/j.apsoil.2021.104064
10.1016/j.apsoil.2021.104065
10.1016/j.apsoil.2021.104111
10.1016/j.apsoil.2021.104124
10.1016/j.apsoil.2021.104140
10.1016/j.aquabot.2015.10.003
10.1016/j.baae.2008.10.009
10.1016/j.baae.2012.09.009
10.1016/j.bcab.2021.101914
10.1016/j.bjm.2016.10.012
10.1016/j.bjm.2017.04.005
10.1016/j.bse.2020.104021
10.1016/j.catena.2019.104094
10.1016/j.cej.2021.129925
10.1016/j.chemosphere.2004.10.009
10.1016/j.chemosphere.2005.01.029
10.1016/j.chemosphere.2005.06.053
10.1016/j.chemosphere.2006.01.034
10.1016/j.chemosphere.2006.02.048
10.1016/j.chemosphere.2006.03.022
10.1016/j.chemosphere.2006.07.007
10.1016/j.chemosphere.2008.05.063
10.1016/j.chemosphere.2009.01.046
10.1016/j.chemosphere.2009.02.008
10.1016/j.chemosphere.2009.05.020
10.1016/j.chemosphere.2009.07.042
10.1016/j.chemosphere.2009.08.050
10.1016/j.chemosphere.2013.04.062
10.1016/j.chemosphere.2013.04.093
10.1016/j.chemosphere.2014.03.094
10.1016/j.chemosphere.2014.04.023
10.1016/j.chemosphere.2014.08.019
10.1016/j.chemosphere.2015.04.023
10.1016/j.chemosphere.2015.12.076
10.1016/j.chemosphere.2016.04.049
10.1016/j.chemosphere.2016.12.093
10.1016/j.chemosphere.2017.08.021
10.1016/j.chemosphere.2017.08.079
10.1016/j.chemosphere.2017.12.025
10.1016/j.chemosphere.2018.06.092
10.1016/j.chemosphere.2018.06.099
10.1016/j.chemosphere.2018.09.143
10.1016/j.chemosphere.2019.03.015
10.1016/j.chemosphere.2019.124914
10.1016/j.chemosphere.2020.126791
10.1016/j.chemosphere.2020.127046
10.1016/j.chemosphere.2020.127337
10.1016/j.chemosphere.2020.128924
10.1016/j.chemosphere.2020.129321
10.1016/j.chemosphere.2020.129407
10.1016/j.chemosphere.2021.130272
10.1016/j.colsurfb.2007.04.023
10.1016/j.cropro.2004.08.012
10.1016/j.cropro.2010.12.010
10.1016/j.cropro.2014.07.004
10.1016/j.cropro.2016.03.011
10.1016/j.crvi.2011.05.001
10.1016/j.crvi.2016.04.009
10.1016/j.cub.2020.02.087
10.1016/j.ecoenv.2014.12.033
10.1016/j.ecoenv.2016.06.012
10.1016/j.ecoenv.2017.10.048
10.1016/j.ecoenv.2017.11.011
10.1016/j.ecoenv.2017.12.017
10.1016/j.ecoenv.2018.03.073
10.1016/j.ecoenv.2018.06.004
10.1016/j.ecoenv.2018.09.030
10.1016/j.ecoenv.2018.12.093
10.1016/j.ecoenv.2018.12.097
10.1016/j.ecoenv.2019.02.073
10.1016/j.ecoenv.2019.04.025
10.1016/j.ecoenv.2019.04.055
10.1016/j.ecoenv.2019.109383
10.1016/j.ecoenv.2019.109744
10.1016/j.ecoenv.2019.109783
10.1016/j.ecoenv.2019.109935
10.1016/j.ecoenv.2020.110450
10.1016/j.ecoenv.2020.110485
10.1016/j.ecoenv.2020.110988
10.1016/j.ecoenv.2020.111122
10.1016/j.ecoenv.2020.111196
10.1016/j.ecoenv.2020.111315
10.1016/j.ecoenv.2020.111495
10.1016/j.ecoenv.2020.111599
10.1016/j.ecoenv.2021.111996
10.1016/j.ecoenv.2021.112023
10.1016/j.ecoenv.2021.112154
10.1016/j.ecoenv.2021.112170
10.1016/j.ecoenv.2021.112270
10.1016/j.ecoenv.2021.112782
10.1016/j.ecoleng.2021.106191
10.1016/j.ecolind.2010.09.001
10.1016/j.ecolind.2013.12.004
10.1016/j.ecolind.2020.106917
10.1016/j.ecolind.2021.107800
10.1016/j.ecolind.2021.108193
10.1016/j.ecolind.2021.108209
10.1016/j.ecolind.2021.108377
10.1016/j.ejsobi.2005.12.006
10.1016/j.ejsobi.2007.10.001
10.1016/j.ejsobi.2010.01.001
10.1016/j.ejsobi.2010.05.006
10.1016/j.ejsobi.2010.06.002
10.1016/j.ejsobi.2011.12.005
10.1016/j.ejsobi.2012.11.001
10.1016/j.ejsobi.2013.06.003
10.1016/j.ejsobi.2016.03.003
10.1016/j.ejsobi.2017.05.007
10.1016/j.ejsobi.2020.103217
10.1016/j.ejsobi.2020.103272
10.1016/j.envexpbot.2005.05.011
10.1016/j.envexpbot.2006.06.001
10.1016/j.envexpbot.2006.11.001
10.1016/j.envexpbot.2007.10.008
10.1016/j.envexpbot.2008.09.008

10.1016/j.envexpbot.2009.03.002
10.1016/j.envexpbot.2009.04.005
10.1016/j.envexpbot.2009.08.009
10.1016/j.envexpbot.2009.11.009
10.1016/j.envexpbot.2010.02.005
10.1016/j.envexpbot.2011.04.008
10.1016/j.envexpbot.2011.08.012
10.1016/j.envexpbot.2012.03.003
10.1016/j.envexpbot.2012.11.005
10.1016/j.envexpbot.2013.10.002
10.1016/j.envexpbot.2013.10.005
10.1016/j.envexpbot.2013.11.007
10.1016/j.envexpbot.2013.11.016
10.1016/j.envexpbot.2014.03.007
10.1016/j.envexpbot.2016.06.007
10.1016/j.envexpbot.2016.06.015
10.1016/j.envexpbot.2018.09.005
10.1016/j.envexpbot.2019.01.001
10.1016/j.envexpbot.2019.02.022
10.1016/j.envexpbot.2019.04.015
10.1016/j.envexpbot.2019.103821
10.1016/j.envexpbot.2019.103824
10.1016/j.envexpbot.2019.103925
10.1016/j.envexpbot.2019.103926
10.1016/j.envexpbot.2019.103950
10.1016/j.envexpbot.2020.104034
10.1016/j.envexpbot.2020.104049
10.1016/j.envexpbot.2020.104088
10.1016/j.envexpbot.2020.104096
10.1016/j.envexpbot.2020.104159
10.1016/j.envexpbot.2021.104402
10.1016/j.envint.2005.05.041
10.1016/j.envpol.2004.01.004
10.1016/j.envpol.2004.02.015
10.1016/j.envpol.2005.05.009
10.1016/j.envpol.2005.08.020
10.1016/j.envpol.2006.07.001
10.1016/j.envpol.2007.04.005
10.1016/j.envpol.2007.07.012
10.1016/j.envpol.2008.01.003
10.1016/j.envpol.2008.12.030
10.1016/j.envpol.2015.08.029
10.1016/j.envpol.2016.07.010
10.1016/j.envpol.2017.01.071
10.1016/j.envpol.2018.04.138
10.1016/j.envpol.2018.06.003
10.1016/j.envpol.2020.114585
10.1016/j.envpol.2020.115980
10.1016/j.envpol.2021.116499
10.1016/j.envpol.2021.116758
10.1016/j.envres.2020.110203
10.1016/j.fcr.2011.02.002
10.1016/j.fcr.2020.108030
10.1016/j.fcr.2020.108035
10.1016/j.fcr.2021.108292
10.1016/j.fgb.2004.10.007
10.1016/j.fgb.2008.09.013
10.1016/j.fgb.2014.08.003
10.1016/j.flora.2016.07.016
10.1016/j.flora.2017.05.011
10.1016/j.foreco.2004.03.018
10.1016/j.foreco.2004.07.038
10.1016/j.foreco.2007.10.042
10.1016/j.foreco.2012.10.019
10.1016/j.foreco.2014.01.015
10.1016/j.foreco.2014.12.012
10.1016/j.foreco.2016.08.006
10.1016/j.foreco.2017.01.025
10.1016/j.foreco.2017.08.010
10.1016/j.foreco.2018.05.004
10.1016/j.foreco.2019.117456
10.1016/j.foreco.2020.118039
10.1016/j.foreco.2020.118078
10.1016/j.foreco.2020.118091
10.1016/j.foreco.2020.118309
10.1016/j.foreco.2020.118659
10.1016/j.foreco.2020.118899
10.1016/j.foreco.2020.118901
10.1016/j.foreco.2021.119182
10.1016/j.foreco.2021.119378
10.1016/j.foreco.2021.119464
10.1016/j.foreco.2021.119478
10.1016/j.foreco.2021.119549
10.1016/j.foreco.2021.119670
10.1016/j.foreco.2021.119709
10.1016/j.funbio.2010.03.008
10.1016/j.funbio.2010.08.004
10.1016/j.funbio.2011.08.005
10.1016/j.funbio.2014.03.002
10.1016/j.funbio.2014.04.011
10.1016/j.funbio.2015.11.007
10.1016/j.funbio.2017.11.003
10.1016/j.funbio.2018.12.006
10.1016/j.funbio.2020.11.011
10.1016/j.funbio.2021.01.001
10.1016/j.funbio.2021.08.003
10.1016/j.funeco.2011.08.007
10.1016/j.funeco.2011.12.001
10.1016/j.funeco.2013.05.001
10.1016/j.funeco.2013.08.004
10.1016/j.funeco.2013.11.003
10.1016/j.funeco.2015.03.005
10.1016/j.funeco.2015.03.009
10.1016/j.funeco.2016.01.003
10.1016/j.funeco.2016.04.001
10.1016/j.funeco.2016.05.011
10.1016/j.funeco.2016.10.002
10.1016/j.funeco.2016.11.001
10.1016/j.funeco.2017.02.004
10.1016/j.funeco.2017.05.003
10.1016/j.funeco.2018.11.008
10.1016/j.funeco.2018.12.008
10.1016/j.funeco.2018.12.010
10.1016/j.funeco.2018.12.013
10.1016/j.funeco.2019.04.005
10.1016/j.funeco.2020.101013
10.1016/j.funeco.2020.101019
10.1016/j.funeco.2021.101060
10.1016/j.geoderma.2017.10.002
10.1016/j.geoderma.2018.10.029
10.1016/j.geoderma.2019.114034
10.1016/j.geoderma.2020.114273
10.1016/j.geoderma.2020.114470
10.1016/j.geoderma.2021.115011
10.1016/j.geoderma.2021.115099
10.1016/j.geoderma.2021.115191
10.1016/j.geoderma.2021.115283
10.1016/j.heliyon.2020.e05891
10.1016/j.heliyon.2021.e06022
10.1016/j.indcrop.2015.03.093
10.1016/j.indcrop.2015.07.009
10.1016/j.indcrop.2015.12.021
10.1016/j.indcrop.2018.02.087
10.1016/j.indcrop.2018.12.038
10.1016/j.indcrop.2019.111934
10.1016/j.indcrop.2020.112234
10.1016/j.indcrop.2020.112763
10.1016/j.indcrop.2020.113136
10.1016/j.indcrop.2021.113792
10.1016/j.indcrop.2021.114002
10.1016/j.jamiap.2016.09.0011
10.1016/j.jaridenv.2009.03.004
10.1016/j.jaridenv.2010.05.001
10.1016/j.jarmap.2017.04.001
10.1016/j.jarmap.2021.100317
10.1016/j.jbiotec.2004.12.009
10.1016/j.jbiotec.2020.09.003
10.1016/j.jbiotec.2021.03.008
10.1016/j.jece.2019.103316
10.1016/j.jece.2021.106395
10.1016/j.jenvman.2006.05.005
10.1016/j.jenvman.2010.07.008
10.1016/j.jenvman.2011.01.025
10.1016/j.jenvman.2012.05.003
10.1016/j.jenvman.2012.06.015
10.1016/j.jenvman.2017.04.014
10.1016/j.jenvman.2018.10.040
10.1016/j.jenvman.2019.109982
10.1016/j.jenvman.2021.113516
10.1016/j.jes.2015.01.016
10.1016/j.jes.2015.12.024
10.1016/j.jes.2020.06.032
10.1016/j.jhazmat.2009.12.056
10.1016/j.jhazmat.2010.05.042
10.1016/j.jhazmat.2010.09.004

10.1016/j.jhazmat.2012.05.091
10.1016/j.jhazmat.2012.08.020
10.1016/j.jhazmat.2016.05.017
10.1016/j.jhazmat.2017.04.065
10.1016/j.jhazmat.2019.120813
10.1016/j.jhazmat.2019.121655
10.1016/j.jhazmat.2019.121873
10.1016/j.jhazmat.2020.123393
10.1016/j.jhazmat.2020.124123
10.1016/j.jhazmat.2020.124325
10.1016/j.jhazmat.2021.125390
10.1016/j.jhazmat.2021.125814
10.1016/j.jhazmat.2021.126095
10.1016/j.jphotobiol.2019.03.002
10.1016/j.jplph.2004.03.011
10.1016/j.jplph.2004.09.014
10.1016/j.jplph.2005.04.024
10.1016/j.jplph.2005.05.003
10.1016/j.jplph.2005.09.001
10.1016/j.jplph.2006.06.016
10.1016/j.jplph.2006.08.005
10.1016/j.jplph.2007.08.010
10.1016/j.jplph.2009.02.010
10.1016/j.jplph.2010.01.018
10.1016/j.jplph.2010.06.024
10.1016/j.jplph.2010.12.019
10.1016/j.jplph.2012.01.014
10.1016/j.jplph.2012.08.020
10.1016/j.jplph.2013.06.006
10.1016/j.jplph.2014.03.007
10.1016/j.jplph.2014.07.027
10.1016/j.jplph.2015.07.006
10.1016/j.jplph.2015.09.007
10.1016/j.jplph.2016.06.013
10.1016/j.jplph.2017.08.012
10.1016/j.jplph.2020.153115
10.1016/j.jplph.2021.153420
10.1016/j.jplph.2021.153544
10.1016/j.jprot.2011.03.027
10.1016/j.jprot.2012.04.039
10.1016/j.jtemb.2020.126594
10.1016/j.jtusci.2017.02.002
10.1016/j.micres.2010.04.004
10.1016/j.micres.2020.126640
10.1016/j.micres.2020.126688
10.1016/j.micres.2021.126774
10.1016/j.molp.2021.06.029
10.1016/j.myc.2017.04.009
10.1016/j.mycres.2007.06.005
10.1016/j.mycres.2009.05.001
10.1016/j.mycres.2009.09.005
10.1016/j.pedobi.2007.02.003
10.1016/j.pedobi.2007.11.002
10.1016/j.pedobi.2008.04.003
10.1016/j.pedobi.2010.01.001
10.1016/j.pedobi.2011.05.002
10.1016/j.pedobi.2012.06.002
10.1016/j.pedobi.2014.09.004
10.1016/j.pedobi.2019.150595
10.1016/j.pedobi.2019.150612
10.1016/j.pedobi.2020.150665
10.1016/j.pedobi.2020.150690
10.1016/j.pedobi.2020.150691
10.1016/j.pedobi.2020.150700
10.1016/j.pedobi.2021.150731
10.1016/j.pedobi.2021.150744
10.1016/j.phytochem.2008.09.009
10.1016/j.phytochem.2011.12.012
10.1016/j.plantsci.2005.03.013
10.1016/j.plantsci.2012.11.009
10.1016/j.plantsci.2016.09.010
10.1016/j.plantsci.2017.06.006
10.1016/j.plantsci.2018.05.015
10.1016/j.plantsci.2018.06.009
10.1016/j.plantsci.2021.110873
10.1016/j.plaphy.2006.12.008
10.1016/j.plaphy.2012.07.018
10.1016/j.plaphy.2012.08.014
10.1016/j.plaphy.2012.11.016
10.1016/j.plaphy.2015.03.004
10.1016/j.plaphy.2015.04.001
10.1016/j.plaphy.2018.04.016
10.1016/j.plaphy.2018.08.004
10.1016/j.plaphy.2018.09.011
10.1016/j.plaphy.2019.05.013
10.1016/j.plaphy.2019.09.032
10.1016/j.plaphy.2019.10.001
10.1016/j.plaphy.2020.02.020
10.1016/j.plaphy.2020.02.039
10.1016/j.plaphy.2020.03.026
10.1016/j.plaphy.2020.09.011
10.1016/j.plaphy.2020.11.025
10.1016/j.plaphy.2021.02.026
10.1016/j.plaphy.2021.05.025
10.1016/j.pmpp.2020.101522
10.1016/j.pmpp.2021.101691
10.1016/j.pmpp.2021.101708
10.1016/j.pmpp.2021.101733
10.1016/j.ppees.2016.01.005
10.1016/j.quaint.2020.12.024
10.1016/j.rama.2020.08.005
10.1016/j.rama.2021.04.008
10.1016/j.rama.2021.08.001
10.1016/j.rhisph.2016.09.004
10.1016/j.rhisph.2018.02.001
10.1016/j.rhisph.2018.07.002
10.1016/j.rhisph.2018.10.003
10.1016/j.rhisph.2018.11.008
10.1016/j.rhisph.2019.100148
10.1016/j.rhisph.2019.100150
10.1016/j.rhisph.2019.100178
10.1016/j.rhisph.2020.100269
10.1016/j.rhisph.2021.100307
10.1016/j.rhisph.2021.100308
10.1016/j.rhisph.2021.100325
10.1016/j.rhisph.2021.100330
10.1016/j.rhisph.2021.100338
10.1016/j.rhisph.2021.100345
10.1016/j.rhisph.2021.100349
10.1016/j.rhisph.2021.100354
10.1016/j.rhisph.2021.100367
10.1016/j.rhisph.2021.100392
10.1016/j.rhisph.2021.100394
10.1016/j.rhisph.2021.100404
10.1016/j.rhisph.2021.100415
10.1016/j.rhisph.2021.100417
10.1016/j.rhisph.2021.100454
10.1016/j.sajb.2018.10.024
10.1016/j.sajb.2019.06.001
10.1016/j.scienta.2003.09.001
10.1016/j.scienta.2005.07.006
10.1016/j.scienta.2006.02.019
10.1016/j.scienta.2007.05.012
10.1016/j.scienta.2008.07.025
10.1016/j.scienta.2009.01.001
10.1016/j.scienta.2010.09.020
10.1016/j.scienta.2011.03.051
10.1016/j.scienta.2011.08.019
10.1016/j.scienta.2012.03.016
10.1016/j.scienta.2012.06.010
10.1016/j.scienta.2013.06.011
10.1016/j.scienta.2014.10.037
10.1016/j.scienta.2015.02.029
10.1016/j.scienta.2015.02.033
10.1016/j.scienta.2015.06.024
10.1016/j.scienta.2015.09.062
10.1016/j.scienta.2015.11.002
10.1016/j.scienta.2017.10.038
10.1016/j.scienta.2018.08.010
10.1016/j.scienta.2018.08.027
10.1016/j.scienta.2019.01.026
10.1016/j.scienta.2019.04.066
10.1016/j.scienta.2019.108595
10.1016/j.scienta.2019.108652
10.1016/j.scienta.2019.108745
10.1016/j.scienta.2019.108749
10.1016/j.scienta.2019.108923
10.1016/j.scienta.2019.109109
10.1016/j.scienta.2020.109373
10.1016/j.scienta.2020.109394
10.1016/j.scienta.2020.109535
10.1016/j.scienta.2020.109549
10.1016/j.scienta.2020.109712
10.1016/j.scienta.2020.109846
10.1016/j.scienta.2021.109933

10.1016/j.scienta.2021.109937
10.1016/j.scienta.2021.110077
10.1016/j.scienta.2021.110337
10.1016/j.scitotenv.2006.07.008
10.1016/j.scitotenv.2006.07.038
10.1016/j.scitotenv.2008.02.003
10.1016/j.scitotenv.2010.07.064
10.1016/j.scitotenv.2011.10.053
10.1016/j.scitotenv.2012.12.085
10.1016/j.scitotenv.2013.02.036
10.1016/j.scitotenv.2014.12.014
10.1016/j.scitotenv.2016.02.100
10.1016/j.scitotenv.2016.05.178
10.1016/j.scitotenv.2016.07.077
10.1016/j.scitotenv.2016.07.124
10.1016/j.scitotenv.2016.10.091
10.1016/j.scitotenv.2017.01.067
10.1016/j.scitotenv.2017.05.047
10.1016/j.scitotenv.2017.05.063
10.1016/j.scitotenv.2017.08.283
10.1016/j.scitotenv.2017.08.307
10.1016/j.scitotenv.2017.09.066
10.1016/j.scitotenv.2017.09.265
10.1016/j.scitotenv.2017.12.278
10.1016/j.scitotenv.2018.06.014
10.1016/j.scitotenv.2018.09.246
10.1016/j.scitotenv.2018.10.249
10.1016/j.scitotenv.2018.11.317
10.1016/j.scitotenv.2019.05.222
10.1016/j.scitotenv.2019.06.035
10.1016/j.scitotenv.2020.136954
10.1016/j.scitotenv.2020.137040
10.1016/j.scitotenv.2020.137438
10.1016/j.scitotenv.2020.137475
10.1016/j.scitotenv.2020.142631
10.1016/j.scitotenv.2020.143137
10.1016/j.scitotenv.2020.143667
10.1016/j.scitotenv.2020.143825
10.1016/j.scitotenv.2020.143945
10.1016/j.scitotenv.2020.144453
10.1016/j.scitotenv.2021.145133
10.1016/j.scitotenv.2021.145672
10.1016/j.scitotenv.2021.146461
10.1016/j.scitotenv.2021.146581
10.1016/j.scitotenv.2021.147943
10.1016/j.scitotenv.2021.147944
10.1016/j.scitotenv.2021.148015
10.1016/j.scitotenv.2021.148522
10.1016/j.scitotenv.2021.148720
10.1016/j.scitotenv.2021.148737
10.1016/j.scitotenv.2021.149222
10.1016/j.scitotenv.2021.149481
10.1016/j.scitotenv.2021.149774
10.1016/j.sjbs.2010.06.007
10.1016/j.sjbs.2013.12.005
10.1016/j.sjbs.2015.03.004
10.1016/j.sjbs.2015.11.002
10.1016/j.sjbs.2015.11.007
10.1016/j.sjbs.2016.02.010
10.1016/j.sjbs.2016.11.003
10.1016/j.sjbs.2016.11.015
10.1016/j.sjbs.2017.10.015
10.1016/j.sjbs.2018.03.009
10.1016/j.sjbs.2018.11.005
10.1016/j.sjbs.2019.10.008
10.1016/j.sjbs.2020.11.018
10.1016/j.sjbs.2021.02.070
10.1016/j.sjbs.2021.03.064
10.1016/j.sjbs.2021.05.019
10.1016/j.sjbs.2021.06.025
10.1016/j.sjbs.2021.08.016
10.1016/j.soilbio.2004.07.029
10.1016/j.soilbio.2005.03.005
10.1016/j.soilbio.2005.10.016
10.1016/j.soilbio.2005.11.011
10.1016/j.soilbio.2007.05.008
10.1016/j.soilbio.2008.01.025
10.1016/j.soilbio.2008.04.016
10.1016/j.soilbio.2008.08.011
10.1016/j.soilbio.2008.12.007
10.1016/j.soilbio.2009.01.009
10.1016/j.soilbio.2009.02.016
10.1016/j.soilbio.2009.05.014
10.1016/j.soilbio.2009.10.021
10.1016/j.soilbio.2009.11.021
10.1016/j.soilbio.2010.11.002
10.1016/j.soilbio.2011.06.001
10.1016/j.soilbio.2011.08.007
10.1016/j.soilbio.2011.08.011
10.1016/j.soilbio.2012.08.025
10.1016/j.soilbio.2012.10.001
10.1016/j.soilbio.2012.10.031
10.1016/j.soilbio.2012.11.002
10.1016/j.soilbio.2013.05.004
10.1016/j.soilbio.2013.10.010
10.1016/j.soilbio.2013.12.010
10.1016/j.soilbio.2014.03.010
10.1016/j.soilbio.2014.05.002
10.1016/j.soilbio.2014.06.010
10.1016/j.soilbio.2014.07.025
10.1016/j.soilbio.2015.01.023
10.1016/j.soilbio.2015.03.009
10.1016/j.soilbio.2015.05.028
10.1016/j.soilbio.2015.06.019
10.1016/j.soilbio.2015.07.007
10.1016/j.soilbio.2015.08.011
10.1016/j.soilbio.2015.10.002
10.1016/j.soilbio.2015.12.005
10.1016/j.soilbio.2015.12.016
10.1016/j.soilbio.2016.11.020
10.1016/j.soilbio.2017.01.011
10.1016/j.soilbio.2017.05.028
10.1016/j.soilbio.2017.07.024
10.1016/j.soilbio.2017.08.019
10.1016/j.soilbio.2017.08.024
10.1016/j.soilbio.2017.09.023
10.1016/j.soilbio.2018.01.033
10.1016/j.soilbio.2018.03.009
10.1016/j.soilbio.2018.09.019
10.1016/j.soilbio.2018.09.031
10.1016/j.soilbio.2018.12.021
10.1016/j.soilbio.2019.02.005
10.1016/j.soilbio.2019.107572
10.1016/j.soilbio.2019.107594
10.1016/j.soilbio.2019.107600
10.1016/j.soilbio.2019.107611
10.1016/j.soilbio.2019.107634
10.1016/j.soilbio.2019.107643
10.1016/j.soilbio.2020.107732
10.1016/j.soilbio.2020.107734
10.1016/j.soilbio.2020.107764
10.1016/j.soilbio.2020.107798
10.1016/j.soilbio.2020.107833
10.1016/j.soilbio.2020.108021
10.1016/j.soilbio.2020.108075
10.1016/j.soilbio.2020.108102
10.1016/j.soilbio.2021.108151
10.1016/j.soilbio.2021.108154
10.1016/j.soilbio.2021.108163
10.1016/j.soilbio.2021.108179
10.1016/j.soilbio.2021.108208
10.1016/j.soilbio.2021.108243
10.1016/j.soilbio.2021.108299
10.1016/j.soilbio.2021.108305
10.1016/j.soilbio.2021.108361
10.1016/j.soilbio.2021.108366
10.1016/j.soilbio.2021.108386
10.1016/j.soilbio.2021.108396
10.1016/j.soilbio.2024.109414
10.1016/j.still.2006.04.001
10.1016/j.still.2018.08.010
10.1016/j.still.2020.104885
10.1016/j.ufug.2015.04.011
10.1016/j.ufug.2021.127050
10.1016/S0007-1536(78)80144-2
10.1016/S0016-7061(97)00118-3
10.1016/S0038-0717(01)00028-1
10.1016/S0038-0717(02)00024-X
10.1016/S0038-0717(03)00004-X
10.1016/S0038-0717(97)00204-6
10.1016/S0038-0717(98)00073-X
10.1016/S0038-0717(99)00059-0
10.1016/S0038-0717(99)00223-0
10.1016/S0045-6535(00)00125-9
10.1016/S0045-6535(00)00126-0

10.1016/S0045-6535(02)00227-8
10.1016/S0045-6535(02)00228-X
10.1016/S0045-6535(02)00229-1
10.1016/S0098-8472(97)00003-8
10.1016/S0098-8472(98)00039-2
10.1016/S0098-8472(99)00039-8
10.1016/S0167-7012(99)00010-X
10.1016/S0167-8809(02)00044-0
10.1016/S0167-8809(96)01074-2
10.1016/S0167-8809(97)00151-5
10.1016/S0168-9452(01)00416-2
10.1016/S0168-9452(96)04533-5
10.1016/S0176-1617(11)80339-1
10.1016/S0176-1617(98)80224-1
10.1016/S0181-1584(01)01076-4
10.1016/S0232-4393(11)80196-4
10.1016/S0269-7491(01)00126-9
10.1016/S0269-7491(03)00195-7
10.1016/S0269-7491(03)00235-5
10.1016/S0269-7491(96)00090-5
10.1016/S0269-7491(99)00095-0
10.1016/S0269-7491(99)00248-1
10.1016/S0269-7491(99)00275-4
10.1016/S0304-4238(01)00293-X
10.1016/S0308-8146(00)00314-9
10.1016/S0308-8146(99)00127-2
10.1016/S0378-1127(03)00147-6
10.1016/S0378-1127(97)00118-7
10.1016/S0378-1127(97)00132-1
10.1016/S0378-1127(97)00138-2
10.1016/S0378-1127(98)00262-X
10.1016/S0378-1127(99)00155-3
10.1016/S0378-1127(99)00220-0
10.1016/S0378-1127(99)00251-0
10.1016/S0929-1393(00)00056-1
10.1016/S0929-1393(01)00129-9
10.1016/S0929-1393(01)00139-1
10.1016/S0929-1393(01)00156-1
10.1016/S0929-1393(02)00160-9
10.1016/S0929-1393(03)00088-X
10.1016/S0929-1393(97)00014-0
10.1016/S0929-1393(97)00025-5
10.1016/S0929-1393(97)00060-7
10.1016/S0929-1393(98)00153-X
10.1016/S0953-7562(09)80449-8
10.1016/S0953-7562(09)81238-0
10.1016/S1001-0742(08)62121-X
10.1016/S1001-0742(08)62406-7
10.1016/S1001-0742(08)62471-7
10.1016/S1001-0742(11)60721-3
10.1016/S1001-0742(11)60898-X
10.1016/S1001-0742(12)60060-6
10.1016/S1002-0160(08)60071-5
10.1016/S1002-0160(11)60132-X
10.1016/S1002-0160(12)60008-3
10.1016/S1002-0160(14)60029-1
10.1016/S1002-0160(14)60064-3
10.1016/S1002-0160(15)60018-2
10.1016/S1002-0160(19)60832-5
10.1016/S1146-609X(99)80013-7
10.1016/S1164-5563(01)01081-0
10.1016/S1671-2927(09)60202-9
10.1017/inp.2018.32
10.1017/inp.2019.19
10.1017/inp.2021.17
10.1017/S001447970300125X
10.1017/S0021859602002101
10.1017/S0021859615000040
10.1017/S0021859618000023
10.1017/S0043174500057131
10.1017/S0043174500069320
10.1017/S0266467405002348
10.1017/S0266467416000274
10.1017/S0953756201003914
10.1017/S095375620400214X
10.1017/S0953756297004681
10.1017/S0953756297005959
10.1017/S0953756299002099
10.1017/S1742170508002226
10.1021/acs.est.5b03659
10.1021/acs.jafc.1c05576
10.1021/es000288s
10.1021/es1008664
10.1021/es502070m
10.1023/A:1004210709108
10.1023/A:1004216225159
10.1023/A:1004246720186
10.1023/A:1004249629643
10.1023/A:1004286704906
10.1023/A:1004290719824
10.1023/A:1004298804353
10.1023/A:1004299928220
10.1023/A:1004399303192
10.1023/A:1004553315041
10.1023/A:1004692907692
10.1023/A:1004721626216
10.1023/A:1004747004058
10.1023/A:1004806122105
10.1023/A:1004891210871
10.1023/A:1004929801839
10.1023/A:1005021607135
10.1023/A:1008899204502
10.1023/A:1009751703827
10.1023/A:1010207610974
10.1023/A:1010321509628
10.1023/A:1010500400263
10.1023/A:1010564013601
10.1023/A:1012721912136
10.1023/A:1012764902915
10.1023/A:1012975605995
10.1023/A:1014435407735
10.1023/A:1014799008459
10.1023/A:1014989422241
10.1023/A:1015037127126
10.1023/A:1015792204633
10.1023/A:1019943426535
10.1023/A:1021555809036
10.1023/A:1024569615325
10.1023/A:1024843419670
10.1023/A:1025479701246
10.1023/A:1025539100767
10.1023/A:1026475925703
10.1023/A:1026496420809
10.1023/A:1026565701391
10.1023/B:BIOP.0000047157.49910.69
10.1023/B:PLSO.0000035538.09222.ff
10.1023/B:VEGE.0000026031.14086.f1
10.1023/B:WATE.0000015335.32888.36
10.1023/B:WATE.0000026527.34649.3c
10.1029/2021GL092764
10.1034/j.1399-3054.1996.980413.x
10.1034/j.1399-3054.1998.1020217.x
10.1034/j.1399-3054.1999.106403.x
10.1034/j.1399-3054.2001.1110307.x
10.1034/j.1600-0706.2002.970105.x
10.1034/j.1600-0706.2003.12006.x
10.1038/ismej.2013.209
10.1038/ismej.2015.204
10.1038/nature03268
10.1038/s41396-020-0614-6
10.1038/s41396-021-00894-1
10.1038/s41396-021-01006-9
10.1038/s41396-021-01133-3
10.1038/s41438-021-00524-z
10.1038/s41467-021-23605-y
10.1038/s41467-021-25652-x
10.1038/s41559-021-01401-7
10.1038/s41598-017-11083-6
10.1038/s41598-018-20456-4
10.1038/s41598-020-68112-0
10.1038/s41598-020-69213-6
10.1038/s41598-021-02018-3
10.1038/s41598-021-84002-5
10.1038/s41598-021-84284-9
10.1038/s41598-021-86758-2
10.1038/s41598-021-89448-1
10.1038/s41598-021-94681-9
10.1038/s41598-021-97674-w
10.1038/s41598-021-97742-1
10.1038/s41598-024-58486-w
10.1038/s41893-021-00791-7
10.1038/srep05634
10.1038/srep17546
10.1038/srep19990
10.1038/srep20245
10.1038/srep20469
10.1038/srep21805
10.1038/srep24749
10.1038/srep37663
10.1038/srep41134
10.1038/srep42335
10.1038/srep42389
10.1039/c3mt00061c
10.1039/c5mt00024f
10.1039/c7mt00072c
10.1039/c7mt90028g
10.1039/c8ra00721g
10.1039/c8ra10442e

10.1046/j.0028-646x.2001.00187.x
10.1046/j.0028-646x.2001.00196.x
10.1046/j.0028-646X.2001.00276.x
10.1046/j.0028-646X.2001.00294.x
10.1046/j.0269-8463.2001.00538.x
10.1046/j.1351-0754.2003.0565.x
10.1046/j.1365-2435.1997.00141.x
10.1046/j.1365-2435.1999.00307.x
10.1046/j.1365-2435.2002.00676.x
10.1046/j.1365-2486.1997.00085.x
10.1046/j.1365-2486.1999.00230.x
10.1046/j.1365-2486.1999.00251.x
10.1046/j.1365-2486.1999.00278.x
10.1046/j.1365-2486.2000.00277.x
10.1046/j.1365-2486.2000.00290.x
10.1046/j.1365-2486.2001.00404.x
10.1046/j.1365-2486.2003.00560.x
10.1046/j.1365-2486.2003.00582.x
10.1046/j.1365-2486.2003.00593.x
10.1046/j.1365-294X.2003.01967.x
10.1046/j.1365-3040.1998.00337.x
10.1046/j.1365-3040.2000.00637.x
10.1046/j.1432-1327.2001.02216.x
10.1046/j.1439-0329.2000.00200.x
10.1046/j.1461-0248.2001.00251.x
10.1046/j.1461-0248.2003.00460.x
10.1046/j.1469-8137.1997.00780.x
10.1046/j.1469-8137.1997.00842.x
10.1046/j.1469-8137.1998.00141.x
10.1046/j.1469-8137.1998.00200.x
10.1046/j.1469-8137.1998.00204.x
10.1046/j.1469-8137.1998.00206.x
10.1046/j.1469-8137.1998.00306.x
10.1046/j.1469-8137.1998.00892.x
10.1046/j.1469-8137.1999.00372.x
10.1046/j.1469-8137.1999.00376.x
10.1046/j.1469-8137.1999.00381.x
10.1046/j.1469-8137.1999.00408.x
10.1046/j.1469-8137.1999.00479.x
10.1046/j.1469-8137.2000.00598.x
10.1046/j.1469-8137.2000.00610.x
10.1046/j.1469-8137.2000.00653.x
10.1046/j.1469-8137.2000.00694.x
10.1046/j.1469-8137.2000.00744.x
10.1046/j.1469-8137.2001.00013.x
10.1046/j.1469-8137.2001.00081.x
10.1046/j.1469-8137.2002.00367.x
10.1046/j.1469-8137.2002.00404.x
10.1046/j.1469-8137.2002.00470.x
10.1046/j.1469-8137.2003.00658.x
10.1046/j.1469-8137.2003.00682.x
10.1046/j.1469-8137.2003.00696.x
10.1046/j.1469-8137.2003.00865.x
10.1046/j.1469-8137.2003.00941.x
10.1051/agro:2000127
10.1051/agro:2003037
10.1051/agro:2005059
10.1051/forest:19960404
10.1051/forest:2008007
10.1051/forest:2008073
10.1055/s-2005-872893
10.1055/s-2006-924106
10.1055/s-2006-924489
10.1055/s-2006-955916
10.1055/s-2006-955946
10.1071/AR9910835
10.1071/BT01005
10.1071/BT20102
10.1071/CP11193
10.1071/CP13441
10.1071/CP14089
10.1071/CP15212
10.1071/CP16038
10.1071/CP17087
10.1071/CP19264
10.1071/CP20459
10.1071/FP04069
10.1071/FP06340
10.1071/FP07218
10.1071/FP09309
10.1071/PP96089
10.1071/SR09169
10.1073/pnas.0912421107
10.1078/0176-1617-00311
10.1078/0176-1617-01191
10.1078/0944-5013-00139
10.1079/SUM2005284
10.1080/00087114.2007.10589564
10.1080/00103624.2014.956888
10.1080/00103624.2014.981271
10.1080/00103624.2015.1069323
10.1080/00103624.2015.1103252
10.1080/00103624.2016.1146897
10.1080/00103624.2016.1216561
10.1080/00103624.2017.1358740
10.1080/00103624.2018.1431265
10.1080/00103624.2018.1435798
10.1080/00103624.2018.1538376
10.1080/00103624.2018.1563102
10.1080/00103624.2019.1566919
10.1080/00103624.2019.1695828
10.1080/00103624.2020.1784917
10.1080/00103624.2021.1879124
10.1080/00380768.1995.10419612
10.1080/00380768.1996.10416325
10.1080/00380768.2000.10408761
10.1080/00380768.2000.10408762
10.1080/00380768.2014.936037
10.1080/00380768.2014.985578
10.1080/00380768.2018.1490631
10.1080/00380768.2018.1543534
10.1080/00380768.2019.1684808
10.1080/00380768.2020.1851144
10.1080/01448765.1990.9754541
10.1080/01904160009382046
10.1080/01904160009382120
10.1080/01904160500203606
10.1080/01904160801895027
10.1080/01904160802043296
10.1080/01904160903150941
10.1080/01904167.2012.733051
10.1080/01904167.2012.738275
10.1080/01904167.2013.816733
10.1080/01904167.2013.849732
10.1080/01904167.2013.868478
10.1080/01904167.2014.889149
10.1080/01904167.2014.963114
10.1080/01904167.2015.1043374
10.1080/01904167.2015.1087030
10.1080/01904167.2015.1108441
10.1080/01904167.2016.1143499
10.1080/01904167.2016.1170851
10.1080/01904167.2016.1201499
10.1080/01904167.2016.1246567
10.1080/01904167.2016.1263332
10.1080/01904167.2016.1264600
10.1080/01904167.2016.1267208
10.1080/01904167.2017.1406107
10.1080/01904167.2019.1628972
10.1080/01904167.2019.1701029
10.1080/01904167.2020.1711940
10.1080/01904167.2020.1766069
10.1080/01904167.2020.1845375
10.1080/01904167.2021.1871748
10.1080/01904167.2021.1881552
10.1080/01904167.2021.1927087
10.1080/01904167.2021.1952228
10.1080/01904169409364742
10.1080/01904169409364877
10.1080/01904169509364896
10.1080/01904169809365401
10.1080/01904169809365451
10.1080/01904169909365753
10.1080/02757540.2017.1391798
10.1080/02757540.2018.1437150
10.1080/02757540.2019.1705285
10.1080/02827580410030181
10.1080/02827581.2015.1080295
10.1080/02827581.2021.1890206
10.1080/02827589209382710
10.1080/02827589209382744
10.1080/03601230902935139
10.1080/03650340.2010.506481
10.1080/03650340.2011.653683
10.1080/03650340.2013.853289
10.1080/03650340.2013.867950
10.1080/03650340.2015.1051471
10.1080/03650340.2016.1175556
10.1080/03650340.2016.1222609
10.1080/03650340.2018.1493724
10.1080/03650340.2018.1563780
10.1080/03650340.2018.1564907
10.1080/03650340.2019.1607313
10.1080/03650340.2020.1775816
10.1080/03650340.2020.1800645
10.1080/03650340.2021.1878497
10.1080/03650340.2021.1949709
10.1080/03650340.2021.1983174
10.1080/09064710.2016.1204467

10.1080/09064710.2021.1952300
10.1080/09064710510008694
10.1080/09064710510029169
10.1080/09583157.2020.1833304
10.1080/0972060X.2014.890085
10.1080/0972060X.2019.1604166
10.1080/0972060X.2020.1727366
10.1080/10412905.2018.1512533
10.1080/10549811.2019.1602057
10.1080/10549811.2020.1758151
10.1080/10889868.2013.847401
10.1080/10889868.2014.938726
10.1080/10889868.2014.995371
10.1080/10889868.2021.1900052
10.1080/10934529409376086
10.1080/112635004000011837
10.1080/11263504.2010.539851
10.1080/11263504.2013.845268
10.1080/11263504.2014.994575
10.1080/11263504.2020.1813832
10.1080/11263504.2020.1829727
10.1080/11263504.2021.1881644
10.1080/11263504.2021.1985006
10.1080/11956860.2020.1802934
10.1080/12298093.2021.1938803
10.1080/13102818.2020.1837011
10.1080/14620316.2003.11511576
10.1080/14620316.2008.11512413
10.1080/14620316.2014.11513137
10.1080/14620316.2015.11513211
10.1080/15226510009359034
10.1080/15226510108500057
10.1080/15226510108500058
10.1080/15226510208500083
10.1080/15226510701476214
10.1080/15226510701827002
10.1080/15226510902787310
10.1080/15226510903051740
10.1080/15226510903535056
10.1080/15226511003671353
10.1080/15226514.2011.573822
10.1080/15226514.2012.716099
10.1080/15226514.2013.798619
10.1080/15226514.2013.876968
10.1080/15226514.2014.898023
10.1080/15226514.2014.922928
10.1080/15226514.2014.989311
10.1080/15226514.2015.1021952
10.1080/15226514.2015.1045131
10.1080/15226514.2015.1045134
10.1080/15226514.2016.1216079
10.1080/15226514.2016.1225289
10.1080/15226514.2016.1244155
10.1080/15226514.2017.1328390
10.1080/15226514.2018.1438358
10.1080/15226514.2018.1438360
10.1080/15226514.2018.1474443
10.1080/15226514.2018.1537249
10.1080/15226514.2018.1556584
10.1080/15226514.2020.1775548
10.1080/15226514.2020.1803206
10.1080/15226514.2020.1812506
10.1080/15226514.2020.1812507
10.1080/15226514.2020.1813076
10.1080/15226514.2021.1901852
10.1080/15226514.2021.1985960
10.1080/15226514.2021.2002809
10.1080/15230430.2021.1951949
10.1080/15320383.2019.1657381
10.1080/15320383.2021.1887809
10.1080/15320383.2021.1893647
10.1080/15320383.2021.1910623
10.1080/15320383.2021.1963670
10.1080/15324982.2017.1406413
10.1080/15324989009381237
10.1080/15324989509385883
10.1080/15538362.2019.1678448
10.1080/15569543.2021.1992444
10.1080/15592324.2020.1813998
10.1080/16226510490888820
10.1080/17429145.2014.949886
10.1080/17429145.2015.1052025
10.1080/17429145.2016.1262914
10.1080/17429145.2019.1662101
10.1080/17429145.2020.1866091
10.1080/17429145.2021.1934131
10.1080/17550874.2014.992488
10.1080/21501203.2021.1876778
10.1080/23311932.2021.1935529
10.1081/CSS-120024064
10.1081/PLN-100106983
10.1081/PLN-120014702
10.1081/PLN-120016500
10.1081/PLN-120027547
10.1081/PLN-120027552
10.1086/696686
10.1088/1748-9326/abd5e1
10.1089/ees.2016.0460
10.1093/aob/mcaa191
10.1093/aob/mcn238
10.1093/aob/mcq170
10.1093/aob/mcs007
10.1093/aobpla/plaa002
10.1093/aobpla/plw018
10.1093/aobpla/plx004
10.1093/femsec/fiaa108
10.1093/femsec/fiaa174
10.1093/femsec/fiab027
10.1093/femsec/fiab059
10.1093/femsec/fiab109
10.1093/femsec/fiv062
10.1093/femsec/fiv095
10.1093/femsec/fiw113
10.1093/femsec/fiw186
10.1093/femsec/fix036
10.1093/forsci/fxz047
10.1093/forsci/fxz060
10.1093/jexbot/50.335.853
10.1093/jexbot/51.352.1931
10.1093/jexbot/52.364.2241
10.1093/jexbot/53.371.1177
10.1093/jpe/rtaa102
10.1093/jpe/rtab017
10.1093/jpe/rtab050
10.1093/jpe/rtt031
10.1093/jpe/rtw075
10.1093/jpe/rtw097
10.1093/jxb/29.5.1029
10.1093/jxb/29.6.1431
10.1093/jxb/31.6.1687
10.1093/jxb/46.10.1543
10.1093/jxb/46.3.297
10.1093/jxb/47.5.683
10.1093/jxb/erab253
10.1093/jxb/erad158
10.1093/jxb/erh049
10.1093/jxb/erh188
10.1093/jxb/eri188
10.1093/jxb/erm009
10.1093/jxb/erm266
10.1093/jxb/ern057
10.1093/jxb/erw383
10.1093/jxb/erz389
10.1093/treephys/10.2.153
10.1093/treephys/14.11.1229
10.1093/treephys/21.10.673
10.1093/treephys/21.2-3.83
10.1093/treephys/24.1.65
10.1093/treephys/26.1.25
10.1093/treephys/26.9.1185
10.1093/treephys/27.1.97
10.1093/treephys/27.3.375
10.1093/treephys/tpaa035
10.1093/treephys/tpaa109
10.1093/treephys/tpp038
10.1093/treephys/tpq032
10.1093/treephys/tpq070
10.1093/treephys/tpv119
10.1093/treephys/tpw125
10.1093/treephys/tpx105
10.1093/treephys/tpx131
10.1093/treephys/tpz039
10.1094/MPMI-04-21-0084-R
10.1094/MPMI-07-12-0178-R
10.1094/MPMI-09-13-0268-R
10.1094/MPMI-09-14-0251-R
10.1094/MPMI-22-9-1169
10.1094/PD-78-0441
10.1094/PDIS.2002.86.12.1318
10.1094/Phyto-64-48
10.1094/Phyto-68-1810
10.1098/rsbl.2014.0375
10.1098/rspb.2007.0804
10.1099/00221287-146-5-1109
10.1099/mic.0.080218-0
10.1104/pp.112.195370
10.1104/pp.16.00307
10.1104/pp.16.00837
10.1104/pp.82.3.765
10.1111/1365-2435.12056
10.1111/1365-2435.12183
10.1111/1365-2435.12910
10.1111/1365-2435.13003
10.1111/1365-2435.13081
10.1111/1365-2435.13787
10.1111/1365-2435.13832
10.1111/1365-2435.13855
10.1111/1365-2435.13911
10.1111/1365-2435.13940
10.1111/1365-2435.13985
10.1111/1365-2664.12070
10.1111/1365-2664.13363
10.1111/1365-2664.14004
10.1111/1365-2745.12117

New Phytologist

10.1111/1365-2745.12558
10.1111/1365-2745.12609
10.1111/1365-2745.12641
10.1111/1365-2745.12731
10.1111/1365-2745.12888
10.1111/1365-2745.12910
10.1111/1365-2745.12983
10.1111/1365-2745.13102
10.1111/1365-2745.13267
10.1111/1365-2745.13292
10.1111/1365-2745.13521
10.1111/1365-2745.13546
10.1111/1365-2745.13746
10.1111/1365-2745.13766
10.1111/1365-2745.13770
10.1111/1365-2745.13774
10.1111/1365-2745.13807
10.1111/1365-2745.13810
10.1111/1462-2920.13149
10.1111/1462-2920.13729
10.1111/1462-2920.13737
10.1111/1462-2920.15368
10.1111/1462-2920.15492
10.1111/1574-6941.12001
10.1111/1574-6941.12361
10.1111/1744-7917.12445
10.1111/aab.12092
10.1111/aec.12567
10.1111/aec.12598
10.1111/btp.12518
10.1111/ecog.00965
10.1111/ejss.12671
10.1111/ejss.12882
10.1111/ejss.13058
10.1111/ejss.13186
10.1111/ejss.13206
10.1111/ele.12378
10.1111/ele.13209
10.1111/ele.13408
10.1111/ele.13886
10.1111/ele.13903
10.1111/gcb.12045
10.1111/gcb.12348
10.1111/gcb.12409
10.1111/gcb.12609
10.1111/gcb.12716
10.1111/gcb.13238
10.1111/gcb.13785
10.1111/gcb.13803
10.1111/gcb.13884
10.1111/gcb.13957
10.1111/gcb.14081
10.1111/gcb.14722
10.1111/gcb.14851
10.1111/gcb.15087
10.1111/gcb.15220
10.1111/gcb.15432
10.1111/gcb.15523
10.1111/gcb.15593
10.1111/gcb.15713
10.1111/gcb.15774
10.1111/gcb.15914
10.1111/gcb.15945
10.1111/gcb.16027
10.1111/geb.13418
10.1111/gfs.12294
10.1111/grs.12299
10.1111/grs.12331
10.1111/j.0022-0477.2004.00917.x
10.1111/j.1365-2311.2009.01105.x
10.1111/j.1365-2311.2012.01399.x
10.1111/j.1365-2389.2005.00775.x
10.1111/j.1365-2389.2007.00962.x
10.1111/j.1365-2427.2006.01608.x
10.1111/j.1365-2486.2003.00713.x
10.1111/j.1365-2486.2004.00853.x
10.1111/j.1365-2486.2004.00861.x
10.1111/j.1365-2486.2005.00905.x
10.1111/j.1365-2486.2005.00933.x
10.1111/j.1365-2486.2005.00967.x
10.1111/j.1365-2486.2006.01149.x
10.1111/j.1365-2486.2007.01313.x
10.1111/j.1365-2486.2007.01347.x
10.1111/j.1365-2486.2007.01535.x
10.1111/j.1365-2486.2008.01549.x
10.1111/j.1365-2486.2008.01555.x
10.1111/j.1365-2486.2008.01567.x
10.1111/j.1365-2486.2008.01691.x
10.1111/j.1365-2486.2008.01714.x
10.1111/j.1365-2486.2008.01760.x
10.1111/j.1365-2486.2009.02036.x
10.1111/j.1365-2486.2009.02079.x
10.1111/j.1365-2486.2010.02318.x
10.1111/j.1365-2486.2012.02686.x
10.1111/j.1365-2672.2009.04414.x
10.1111/j.1365-2745.2006.01160.x
10.1111/j.1365-2745.2006.01202.x
10.1111/j.1365-2745.2007.01239.x
10.1111/j.1365-2745.2007.01349.x
10.1111/j.1365-2745.2008.01389.x
10.1111/j.1365-2745.2009.01549.x
10.1111/j.1365-2745.2011.01858.x
10.1111/j.1365-2745.2012.01995.x
10.1111/j.1365-2745.2012.02000.x
10.1111/j.1365-3040.1995.tb00572.x
10.1111/j.1365-3040.1996.tb00333.x
10.1111/j.1365-3040.2009.02103.x
10.1111/j.1365-3180.2006.00510.x
10.1111/j.1365-3180.2010.00793.x
10.1111/j.1399-3054.1991.tb00123.x
10.1111/j.1399-3054.1995.tb00865.x
10.1111/j.1399-3054.1995.tb05308.x
10.1111/j.1399-3054.1997.tb00027.x
10.1111/j.1399-3054.2004.00421.x
10.1111/j.1399-3054.2008.01170.x
10.1111/j.1399-3054.2009.01237.x
10.1111/j.1399-3054.2012.01586.x
10.1111/j.1438-8677.1994.tb00411.x
10.1111/j.1438-8677.2012.00581.x
10.1111/j.1438-8677.2012.00614.x
10.1111/j.1439-0329.2004.00375.x
10.1111/j.1439-037X.1986.tb00044.x
10.1111/j.1439-037X.1988.tb00676.x
10.1111/j.1439-037X.2008.00349.x
10.1111/j.1439-037X.2012.00533.x
10.1111/j.1461-0248.2006.00959.x
10.1111/j.1461-0248.2009.01303.x
10.1111/j.1461-0248.2012.01827.x
10.1111/j.1462-2920.2005.00868.x
10.1111/j.1462-2920.2007.01512.x
10.1111/j.1462-2920.2009.02082.x
10.1111/j.1469-8137.1979.tb02674.x
10.1111/j.1469-8137.1982.tb04484.x
10.1111/j.1469-8137.1983.tb02692.x
10.1111/j.1469-8137.1986.tb00658.x
10.1111/j.1469-8137.1987.tb00171.x
10.1111/j.1469-8137.1988.tb00253.x
10.1111/j.1469-8137.1991.tb01027.x
10.1111/j.1469-8137.1991.tb04914.x
10.1111/j.1469-8137.1992.tb01064.x
10.1111/j.1469-8137.1992.tb01798.x
10.1111/j.1469-8137.1993.tb03742.x
10.1111/j.1469-8137.1993.tb03855.x
10.1111/j.1469-8137.1993.tb03872.x
10.1111/j.1469-8137.1993.tb03932.x
10.1111/j.1469-8137.1994.tb02971.x
10.1111/j.1469-8137.1994.tb03959.x
10.1111/j.1469-8137.1994.tb07534.x
10.1111/j.1469-8137.1995.tb03033.x
10.1111/j.1469-8137.1995.tb03084.x
10.1111/j.1469-8137.1996.tb01850.x
10.1111/j.1469-8137.1996.tb01861.x
10.1111/j.1469-8137.1996.tb01877.x
10.1111/j.1469-8137.1996.tb01910.x
10.1111/j.1469-8137.1996.tb04518.x
10.1111/j.1469-8137.1996.tb04637.x
10.1111/j.1469-8137.2004.01037.x
10.1111/j.1469-8137.2004.01049.x
10.1111/j.1469-8137.2004.01074.x
10.1111/j.1469-8137.2004.01190.x
10.1111/j.1469-8137.2005.01440.x
10.1111/j.1469-8137.2005.01455.x
10.1111/j.1469-8137.2005.01481.x
10.1111/j.1469-8137.2006.01730.x
10.1111/j.1469-8137.2006.01778.x
10.1111/j.1469-8137.2006.01831.x
10.1111/j.1469-8137.2006.01961.x
10.1111/j.1469-8137.2007.01973.x
10.1111/j.1469-8137.2007.02150.x
10.1111/j.1469-8137.2007.02155.x
10.1111/j.1469-8137.2008.02491.x
10.1111/j.1469-8137.2009.03009.x
10.1111/j.1469-8137.2010.03274.x
10.1111/j.1469-8137.2010.03485.x
10.1111/j.1469-8137.2010.03494.x
10.1111/j.1469-8137.2010.03634.x
10.1111/j.1469-8137.2011.03776.x
10.1111/j.1526-100X.2006.00199.x
10.1111/j.1526-100X.2012.00894.x
10.1111/j.1526-100X.2012.00915.x
10.1111/j.1529-8817.2003.00734.x
10.1111/j.1529-8817.2003.00788.x
10.1111/j.1558-5646.2012.01718.x
10.1111/j.1574-6941.2009.00740.x
10.1111/j.1574-6941.2009.00795.x
10.1111/j.1574-6941.2010.01003.x

10.1111/j.1574-6941.2012.01350.x
10.1111/j.1574-6968.2009.01503.x
10.1111/j.1654-109X.2010.01079.x
10.1111/j.1744-7348.1989.tb03382.x
10.1111/j.1744-7348.2012.00563.x
10.1111/j.1752-4571.2011.00218.x
10.1111/jac.12175
10.1111/jac.12185
10.1111/jac.12335
10.1111/jam.15021
10.1111/jam.15326
10.1111/jvs.12944
10.1111/jvs.13082
10.1111/lam.12084
10.1111/mec.12484
10.1111/mec.13938
10.1111/mec.14536
10.1111/mec.15160
10.1111/mec.15773
10.1111/mec.15900
10.1111/mec.16095
10.1111/mec.16125
10.1111/mec.16242
10.1111/mec.16278
10.1111/nph.12011
10.1111/nph.12273
10.1111/nph.12421
10.1111/nph.13103
10.1111/nph.13122
10.1111/nph.13224
10.1111/nph.13360
10.1111/nph.13709
10.1111/nph.13961
10.1111/nph.14083
10.1111/nph.14314
10.1111/nph.14603
10.1111/nph.15112
10.1111/nph.15208
10.1111/nph.15295
10.1111/nph.15352
10.1111/nph.15542
10.1111/nph.15740
10.1111/nph.15806
10.1111/nph.15819
10.1111/nph.15917
10.1111/nph.16299
10.1111/nph.16359
10.1111/nph.16641
10.1111/nph.16749
10.1111/nph.16841
10.1111/nph.16954
10.1111/nph.17065
10.1111/nph.17077
10.1111/nph.17256
10.1111/nph.17449
10.1111/nph.17479
10.1111/nph.17572
10.1111/nph.17595
10.1111/nph.17623
10.1111/nph.17661
10.1111/nph.17668
10.1111/nph.17755
10.1111/nph.17817
10.1111/nph.17986
10.1111/nph.18126
10.1111/oik.01552
10.1111/oik.02351
10.1111/oik.07610
10.1111/oik.08451
10.1111/pce.12082
10.1111/pce.12183
10.1111/pce.12631
10.1111/pce.12807
10.1111/pce.13551
10.1111/pce.14053
10.1111/pce.14228
10.1111/plb.12948
10.1111/plb.13039
10.1111/plb.13123
10.1111/plb.13164
10.1111/ppa.12483
10.1111/ppl.12336
10.1111/ppl.13454
10.1111/ppl.13490
10.1111/ppl.13562
10.1111/ppl.13570
10.1111/rec.12287
10.1111/rec.12302
10.1111/rec.12548
10.1111/rec.12639
10.1111/rec.13455
10.1111/rec.13469
10.1111/rec.13473
10.1111/rec.13549
10.1111/sum.12069
10.1111/tpj.15550
10.1111/wre.12454
10.1126/sciadv.abe9256
10.1126/science.1224304
10.1128/AEM.00241-21
10.1128/AEM.00251-21
10.1128/AEM.01523-21
10.1128/AEM.02083-14
10.1128/AEM.02142-08
10.1128/AEM.03015-19
10.1128/AEM.04040-14
10.1128/AEM.59.1.129-133.1993
10.1128/AEM.59.8.2642-2647.1993
10.1128/AEM.61.2.456-460.1995
10.1128/AEM.70.11.6643-6649.2004
10.1128/AEM.70.12.7413-7417.2004
10.1128/AEM.70.5.2692-2701.2004
10.1128/AEM.71.11.7279-7284.2005
10.1134/S1021443710040102
10.1134/S1021443712060143
10.1134/S1021443716010155
10.1134/S1021443716040178
10.1134/S1021443720050076
10.1134/S102144372102014X
10.1134/S102144372105006X
10.1134/S1021443721060200
10.1134/S1067413618030050
10.1139/b01-099
10.1139/B04-122
10.1139/b05-084
10.1139/b05-108
10.1139/B06-039
10.1139/B07-104
10.1139/B09-062
10.1139/B11-009
10.1139/B11-028
10.1139/B2012-054
10.1139/b73-201
10.1139/b80-063
10.1139/b89-005
10.1139/b90-057
10.1139/b91-224
10.1139/b93-137
10.1139/b95-023
10.1139/b95-123
10.1139/b97-870
10.1139/b97-875
10.1139/cjb-2013-0169
10.1139/cjb-2014-0249
10.1139/cjb-2015-0093
10.1139/cjb-2015-0210
10.1139/cjb-2015-0238
10.1139/cjb-2017-0137
10.1139/cjb-2020-0162
10.1139/cjb-2020-0181
10.1139/cjfr-2014-0009
10.1139/cjfr-2016-0037
10.1139/cjfr-30-3-360
10.1139/cjfr-31-2-224
10.1139/cjfr-31-4-711
10.1139/cjm-2015-0703
10.1139/cjm-2015-0732
10.1139/cjm-2016-0597
10.1139/facets-2020-0104
10.1139/W03-073
10.1139/W07-119
10.1139/W09-031
10.1139/W2012-080
10.1139/x01-034
10.1139/X03-072
10.1139/X04-083
10.1139/X05-062
10.1139/X06-212
10.1139/X06-213
10.1139/X11-176
10.1139/x82-021
10.1139/x83-128
10.1139/x90-171
10.1139/x92-249
10.1139/x93-174
10.1139/x94-225
10.1139/x98-186
10.1155/2014/923610
10.1163/002825988X00396
10.1163/156854108786161427
10.1186/1471-2229-11-75
10.1186/s12870-018-1292-7
10.1186/s12870-018-1317-2
10.1186/s12870-018-1492-1
10.1186/s12870-021-02945-3
10.1186/s12870-021-02949-z
10.1186/s12870-021-03113-3
10.1186/s12870-021-03125-z
10.1186/s12870-021-03237-6
10.1186/s12898-018-0197-5
10.1186/s13007-020-00700-7
10.1186/s13568-020-01172-7
10.1186/s13717-021-00287-4
10.1186/s40529-014-0070-6
10.1186/s40529-021-00328-3
10.1186/s40529-022-00335-y
10.1186/s40538-017-0102-z
10.1186/s40538-020-00186-4
10.1186/s40538-021-00208-9
10.1186/s40538-021-00226-7
10.1186/s40663-019-0174-2
10.1186/s43008-020-00050-y
10.1186/s43088-021-00166-z
10.1264/jsme2.ME13093
10.12657/denbio.080.009
10.12657/denbio.083.003
10.12871/00021857201844
10.13080/z-a.2016.103.007
10.1353/psc.2006.0033
10.1371/journal.pbio.0040140
10.1371/journal.pone.0027195
10.1371/journal.pone.0028426
10.1371/journal.pone.0035275
10.1371/journal.pone.0038662
10.1371/journal.pone.0048946
10.1371/journal.pone.0061188
10.1371/journal.pone.0076447
10.1371/journal.pone.0080535
10.1371/journal.pone.0080568
10.1371/journal.pone.0103859
10.1371/journal.pone.0128841
10.1371/journal.pone.0132347
10.1371/journal.pone.0142356
10.1371/journal.pone.0145726
10.1371/journal.pone.0145793
10.1371/journal.pone.0149606
10.1371/journal.pone.0172382
10.1371/journal.pone.0184158
10.1371/journal.pone.0188220
10.1371/journal.pone.0196408
10.1371/journal.pone.0219300
10.1371/journal.pone.0231367
10.1371/journal.pone.0231497
10.1371/journal.pone.0234546
10.1371/journal.pone.0244910
10.1371/journal.pone.0248207
10.1371/journal.pone.0253878
10.1371/journal.pone.0254227
10.1371/journal.pone.0272180
10.14393/BJ-v37n0a2021-53697
10.14456/ITJEMAST.2019.39
10.14456/ITJEMAST.2019.42

10.14456/ITJEMAST.2019.49
10.14456/ITJEMAST.2019.59
10.14719/pst.2021.8.4.1240
10.1515/abcsb-2015-0024
10.1515/eces-2016-0035
10.15244/pjoes/125768
10.15244/pjoes/78624
10.15244/pjoes/94012
10.1525/elementa.2021.000059
10.15414/jmbfs.4644
10.15517/am.v32i2.42066
10.1556/018.68.2017.4.4
10.1556/CRC.35.2007.2.59
10.15666/aeer/1501_401413
10.15666/aeer/1601_335357
10.15666/aeer/1703_62416253
10.15666/aeer/1704_99659978
10.15666/aeer/1706_1371313727
10.15666/aeer/1706_1478714804
10.15666/aeer/1802_37413755
10.15666/aeer/1901_699713
10.15666/aeer/1903_21512169
10.15741/revbio.08.e982
10.15835/nbha44110224
10.15835/nbha44210543
10.15835/nbha45110424
10.15835/nbha45110709
10.15835/nbha45110766
10.15835/nbha46110983
10.15835/nbha47111249
10.15835/nbha47411604
10.15835/nbha48311963
10.15835/nbha49112209
10.15835/nbha49211987
10.15835/nbha49311924
10.1590/0001-3765202120201559
10.1590/01000683rbcs20150046
10.1590/0102-33062015abb0190
10.1590/0102-33062020abb0095
10.1590/0103-8478cr20190533
10.1590/01047760202026012671
10.1590/1678-4499.0061
10.1590/1678-4685-GMB-2020-0424
10.1590/1807-1929/agriambi.v18n09p927-933
10.1590/1807-1929/agriambi.v19n6p548-552
10.1590/1807-1929/agriambi.v19n9p898-902
10.1590/1807-1929/agriambi.v22n11p747-752
10.1590/1983-21252018v31n307rc
10.1590/1983-21252019v32n305rc
10.1590/S0100-06832003000300003
10.1590/S0100-06832003000500019
10.1590/S0100-06832006000400007
10.1590/S0100-06832011000400038
10.1590/S0100-204X2001001200001
10.1590/S0100-204X2004000500010
10.1590/S0100-204X2006000200013
10.1590/S0100-67622013000500005
10.1590/S0100-67622014000300008
10.1590/S0100-83582009000500011
10.1590/S0100-83582010000100014
10.1590/S0100-83582010000300018
10.1590/S0100-83582014000400013
10.1590/S0103-90162005000400013
10.1590/S1806-66902010000400008
10.1590/S1806-66902012000300001
10.1614/IPSM-D-10-00004.1
10.1614/IPSM-D-12-00014.1
10.1614/P2002-150
10.1614/WS-D-16-00058.1
10.1674/0003-0031-167.2.213
10.17221/116/2011-PSE
10.17221/198/2020-JFS
10.17221/204/2009-PSE
10.17221/206/2020-PSE
10.17221/2209-PSE
10.17221/23/2011-PSE
10.17221/54/2010-PSE
10.17221/59/2011-PSE
10.17221/594/2020-PSE
10.17221/61/2009-PSE
10.17221/785/2018-PSE
10.17557/tjfc.562627
10.17557/tjfc.564087
10.17660/eJHS.2020/85.1.6
10.17957/IJAB/15.0369
10.17957/IJAB/15.0383
10.17957/IJAB/15.1235
10.17957/IJAB/15.1512
10.18016/ksutarimdoga.vi.882089
10.18052/www.scipress.com/ILNS.76.13
10.18805/1r.v0i0.7298
10.18805/1r.v39i2.9531
10.18805/lr.v0iOF.3770
10.18805/LR-454
10.1890/0012-9658(2003)084[0129:SFATEO]2.0.CO;2
10.1890/0012-9658(2003)084[1895:NEAMAA]2.0.CO;2
10.1890/0012-9658(2006)87[2278:CANAAE]2.0.CO;2
10.1890/02-0413
10.1890/03-5425
10.1890/06-1051
10.1890/07-0370.1
10.1890/07-1394.1
10.1890/07-2080.1
10.1890/07-2144.1
10.1890/08-0419.1
10.1890/08-2139.1
10.1890/09-0204.1
10.1890/09-0336.1
10.1890/09-2366.1
10.1890/10-0459.1
10.1890/1051-0761(2003)013[0565:MDAGPA]2.0.CO;2
10.1890/13-0869.1
10.1890/14-2361.1
10.1890/16-0001.1
10.1890/ES11-00136.1
10.1890/ES12-00344.1
10.20417/nzjecol.45.23
10.20937/RICA.53719
10.2111/08-006.1
10.2112/JCOASTRES-D-20-00078.1
10.21162/PAKJAS/16.2425
10.21273/HORTSCI.19.4.809
10.21273/HORTSCI.26.11.1406
10.21273/HORTSCI.33.7.1217
10.21273/HORTSCI.46.5.799
10.21273/HORTSCI.48.5.568
10.21273/HORTSCI.51.3.212
10.21273/HORTSCI11256-16
10.21273/HORTSCI13587-18
10.21273/HORTSCI13961-19
10.21273/HORTSCI14888-20
10.21273/JASHS.119.6.1255
10.21273/JASHS05084-21
10.2134/agronj13.0589
10.2134/agronj1993.00021962008500020006x
10.2134/agronj1996.00021962008800020007x
10.2134/agronj2007.0142
10.2134/agronj2012.0069
10.2134/agronj2016.10.0597
10.2134/jeq1994.00472425002300050010x
10.2134/jeq1998.00472425002700050030x
10.2134/jeq2003.2001
10.2134/jeq2004.0411
10.2134/jeq2012.0311
10.2134/jeq2016.04.0125
10.2136/sssaj1989.03615995005300040015x
10.2136/sssaj1995.03615995005900040020x
10.2136/sssaj1998.03615995006200050023x
10.2136/sssaj2011.0135
10.2136/sssaj2012.0220
10.2136/sssaj2013.05.0179
10.21608/ejss.2019.17512.1312
10.2179/0008-7475.86.1.1
10.21829/abm128.2021.1870
10.22146/agritech.58541
10.22201/ib.20078706e.2019.90.2868
10.22201/ib.20078706e.2021.92.3238
10.22438/jeb/41/2(SI)/JEB-14
10.2298/BOTSERB2002211T
10.2298/GENSR1902429G
10.2306/scienceasia1513-1874.2009.35.388
10.2307/1352570
10.2307/177065
10.2307/2269614
10.2307/2389946
10.2307/2389991
10.2307/2390007
10.2307/2390177
10.2307/2445936
10.2307/3558450
10.2307/3558453
10.2307/3759836
10.2307/3760634
10.2307/3761295
10.2307/3761860
10.23855/preslia.2021.363
10.23986/afsci.4994
10.23986/afsci.4997
10.23986/afsci.5728
10.23986/afsci.9552
10.24326/asphc.2018.5.7
10.24326/asphc.2021.2.5
10.24326/asphc.2021.4.2
10.24326/asphc.2021.5.7
10.2478/eces-2021-0020
10.2478/s11535-012-0017-4
10.2478/s11756-006-0182-x
10.2478/s11756-009-0001-2
10.2478/s11756-009-0072-0
10.2503/jjshs.75.26
10.2503/jjshs1.81.257
10.25165/j.ijabe.20181106.4019
10.25165/j.ijabe.20191206.4950
10.25252/SE/2021/162265
10.25252/SE/2021/162291
10.26525/jtfs2019.31.4.398
10.26651/allelo.j/2019-46-2-1214
10.2980/19-1-3478
10.30848/PJB2019-6(12)
10.30848/PJB2021-1(41)

10.3159/TORREY-D-15-00062.1
10.3159/TORREY-D-17-00036.1
10.3159/TORREY-D-20-00049.1
10.32404/rean.v7i1.4042
10.32604/phyton.2021.014376
10.32604/phyton.2021.015476
10.32615/ps.2019.048
10.3329/bjb.v50i0.56447
10.3329/bjb.v50i3.55824
10.3354/meps13614
10.3368/er.34.3.209
10.3389/feart.2015.00089
10.3389/fenvs.2021.701653
10.3389/fevo.2020.00125
10.3389/fevo.2021.613119
10.3389/fevo.2021.625519
10.3389/ffgc.2020.00077
10.3389/ffgc.2020.593243
10.3389/ffgc.2020.609216
10.3389/ffgc.2020.620436
10.3389/ffgc.2021.599946
10.3389/ffgc.2021.685827
10.3389/fmicb.2014.00516
10.3389/fmicb.2014.00682
10.3389/fmicb.2015.00335
10.3389/fmicb.2016.01089
10.3389/fmicb.2016.01994
10.3389/fmicb.2016.02063
10.3389/fmicb.2017.02320
10.3389/fmicb.2018.00091
10.3389/fmicb.2018.00418
10.3389/fmicb.2018.00652
10.3389/fmicb.2018.01254
10.3389/fmicb.2018.01339
10.3389/fmicb.2018.01769
10.3389/fmicb.2018.03156
10.3389/fmicb.2019.01807
10.3389/fmicb.2019.02251
10.3389/fmicb.2019.02591
10.3389/fmicb.2020.00341
10.3389/fmicb.2020.00516
10.3389/fmicb.2020.00616
10.3389/fmicb.2020.01428
10.3389/fmicb.2020.526898
10.3389/fmicb.2020.539669
10.3389/fmicb.2020.597745
10.3389/fmicb.2021.522449
10.3389/fmicb.2021.597442
10.3389/fmicb.2021.636009
10.3389/fmicb.2021.667566
10.3389/fmicb.2021.678250
10.3389/fmicb.2021.680267
10.3389/fmicb.2021.686812
10.3389/fmicb.2021.729244
10.3389/fpls.2015.00477
10.3389/fpls.2016.00644
10.3389/fpls.2016.01237
10.3389/fpls.2017.00131
10.3389/fpls.2017.00183
10.3389/fpls.2017.00440
10.3389/fpls.2017.00517
10.3389/fpls.2017.00906
10.3389/fpls.2017.01056
10.3389/fpls.2017.01263
10.3389/fpls.2017.01739
10.3389/fpls.2018.00154
10.3389/fpls.2018.01156
10.3389/fpls.2018.01363
10.3389/fpls.2018.01480
10.3389/fpls.2018.01605
10.3389/fpls.2018.01631
10.3389/fpls.2018.01726
10.3389/fpls.2018.01728
10.3389/fpls.2018.01842
10.3389/fpls.2018.01906
10.3389/fpls.2018.01937
10.3389/fpls.2019.00843
10.3389/fpls.2019.00863
10.3389/fpls.2019.00897
10.3389/fpls.2020.00348
10.3389/fpls.2020.516818
10.3389/fpls.2020.582574
10.3389/fpls.2020.587414
10.3389/fpls.2020.596929
10.3389/fpls.2020.600792
10.3389/fpls.2020.612299
10.3389/fpls.2020.622209
10.3389/fpls.2020.627345
10.3389/fpls.2021.614162
10.3389/fpls.2021.626709
10.3389/fpls.2021.634960
10.3389/fpls.2021.640379
10.3389/fpls.2021.642094
10.3389/fpls.2021.646173
10.3389/fpls.2021.647372
10.3389/fpls.2021.655060
10.3389/fpls.2021.659694
10.3389/fpls.2021.669143
10.3389/fpls.2021.712622
10.3389/fpls.2021.721614
10.3389/fpls.2021.740524
10.3389/fpls.2021.742628
10.3389/fpls.2021.756368
10.3389/fsufs.2020.00131
10.3389/fsufs.2021.596269
10.3390/agriculture10110491
10.3390/agriculture10110520
10.3390/agriculture11040310
10.3390/agriculture11040361
10.3390/agriculture11050419
10.3390/agriculture11080745
10.3390/agriculture8080117
10.3390/agronomy10010071
10.3390/agronomy10020170
10.3390/agronomy10020181
10.3390/agronomy10111657
10.3390/agronomy11020380
10.3390/agronomy11040748
10.3390/agronomy11040767
10.3390/agronomy11050888
10.3390/agronomy11061143
10.3390/agronomy11081602
10.3390/agronomy11101932
10.3390/agronomy11112183
10.3390/agronomy11112322
10.3390/agronomy11112348
10.3390/agronomy11112355
10.3390/agronomy5020188
10.3390/agronomy8010008
10.3390/agronomy8100223
10.3390/agronomy9010041
10.3390/agronomy9090545
10.3390/agronomy9100572
10.3390/agronomy9100637
10.3390/biology8040093
10.3390/cells10081944
10.3390/d13110525
10.3390/f10020186
10.3390/f10121098
10.3390/f11050488
10.3390/f11101117
10.3390/f12010026
10.3390/f12010039
10.3390/f12030332
10.3390/f12050611
10.3390/f12060669
10.3390/f12101410
10.3390/f6041256
10.3390/f8090310
10.3390/f9030099
10.3390/horticulturae5040079
10.3390/horticulturae6030045
10.3390/horticulturae7080217
10.3390/horticulturae7080228
10.3390/horticulturae7100395
10.3390/horticulturae7100397
10.3390/ijms20030788
10.3390/ijms21051748
10.3390/ijms21217987
10.3390/ijms222111651
10.3390/jof6020087
10.3390/jof7040296
10.3390/jof7050361
10.3390/jof7060402
10.3390/jof7060458
10.3390/jof7070531
10.3390/jof7080671
10.3390/jof7080675
10.3390/jof7090686
10.3390/jof7090702
10.3390/jof7090765
10.3390/jof7100791
10.3390/jof7100867
10.3390/jof7110892
10.3390/life11020177
10.3390/metabo11070428
10.3390/microorganisms7090289
10.3390/microorganisms7110505
10.3390/microorganisms7120706
10.3390/microorganisms8030327
10.3390/microorganisms8111695
10.3390/microorganisms8111828
10.3390/microorganisms9010150
10.3390/microorganisms9020229
10.3390/microorganisms9020437
10.3390/microorganisms9030518
10.3390/microorganisms9061273
10.3390/microorganisms9061274
10.3390/microorganisms9061333
10.3390/microorganisms9071366
10.3390/microorganisms9091843
10.3390/microorganisms9112261
10.3390/pathogens10070797
10.3390/plants10020349
10.3390/plants10030583
10.3390/plants10040657
10.3390/plants10050937
10.3390/plants10050950
10.3390/plants10050976
10.3390/plants10071357
10.3390/plants10091756
10.3390/plants10091790
10.3390/plants10102024
10.3390/plants10102196

10.3390/plants10112242
10.3390/plants10112474
10.3390/plants10112501
10.3390/plants7030063
10.3390/plants8120579
10.3390/plants9010042
10.3390/plants9010080
10.3390/plants9010108
10.3390/plants9020148
10.3390/plants9111430
10.3390/plants9111499
10.3390/pr9101810
10.3390/su12156190
10.3390/su13031226
10.3390/su13063244
10.3390/su13126869
10.3390/su13168804
10.3390/su131910866
10.3390/su131911007
10.3390/su132111950
10.3390/su71215799
10.3390/w13091306
10.35495/ajab.2019.12.553
10.36783/18069657rbcs20200163
10.3732/ajb.0800068
10.3732/ajb.1000177
10.3732/ajb.1100497
10.3732/ajb.1600142
10.3732/ajb.94.8.1309
10.3832/ifor1643-008
10.3832/ifor1911-009
10.3832/ifor2725-011
10.3852/08-024R
10.3852/mycologia.97.6.1177
10.3906/biy-0811-3
10.3906/bot-1008-32
10.3906/tar-0908-12
10.3906/tar-1406-121
10.3906/tar-1503-57
10.3989/gya.0572191
10.4067/S0717-66432012000200009
10.4067/S0718-58392013000100005
10.4067/S0718-95162012005000009
10.4067/S0718-95162012005000036
10.4141/CJPS10077
10.4141/CJPS2012-279
10.4141/P02-003
10.4141/P02-199
10.46490/BF424
10.47163/agrociencia.v55i3.2419
10.47371/mycosci.2020.12.002
10.5073/JABFQ.2016.089.011
10.5194/bg-11-321-2014
10.5194/bg-14-4815-2017
10.5194/bg-16-3637-2019
10.5194/bg-18-4143-2021
10.5194/bg-4-769-2007
10.5194/gmd-14-735-2021
10.5423/PPJ.OA.04.2021.0067
10.5424/sjar/20110904-069-11
10.5424/sjar/20110904-307-10
10.5424/sjar/2014123-4815
10.5433/1679-0359.2013v34n4p1587
10.5433/1679-0359.2015v36n3p1253
10.5433/1679-0359.2021v42n6Supl2p3617
10.5511/plantbiotechnology.27.339
10.5513/JCEA01/22.3.3165
10.5558/tfc72293-3
10.5735/085.045.0602
10.5897/AJAR11.101
10.5897/AJMR11.1278
10.5897/JMPR11.1448
10.5902/1980509832052
10.5935/1806-6690.20160051
10.5943/mycosphere/7/6/14
10.5958/0974-0112.2015.00006.7
10.5958/0974-0112.2015.00103.6
10.5958/0974-0112.2016.00050.5
10.5958/j.0976-0571.37.3.049
10.7717/peerj.11115
10.7717/peerj.11792
10.7717/peerj.12076
10.7717/peerj.2083
10.7717/peerj.4425
10.7717/peerj.4478
10.7717/peerj.7650
10.7717/peerj.8991
10.9755/ejfa.2015-11-1044
10.9755/ejfa.2018.v30.i3.1642

Reference list for the supplementary methods

Aria M, Cuccurullo C. 2017. bibliometrix: An R-tool for comprehensive science mapping analysis. *Journal of Informetrics* **11**(4): 959-975.

Bebber DP. 2021. The gap between atmospheric nitrogen deposition experiments and reality. *Science of The Total Environment* **801**: 149774.

Rillig MC, Ryo M, Lehmann A. 2021. Classifying human influences on terrestrial ecosystems. *Global Change Biology* **27**(11): 2273-2278.

Smith SE, Read D 2008. Introduction. In: Smith SE, Read D eds. *Mycorrhizal Symbiosis (Third Edition)*. London: Academic Press, 1-9.
